# Analysis of the Spectrum of Neutral Atomic Bromine (Br I)

**DOI:** 10.6028/jres.067A.051

**Published:** 1963-12-01

**Authors:** Jack L. Tech

## Abstract

The spectrum of the neutral bromine atom, Br I, has been newly investigated by using electrodeless discharge tubes as light sources. The observations have led to a list of wavelengths and estimated intensities for 1253 spectral lines in the range 1067 to 24100 Å. The number of known energy levels has been increased to 123 even and 128 odd levels, as compared with the 27 even and 33 odd levels previously known. All predicted energy levels of the 4*s*^2^4*p*^4^*ns, up, nd, nf* electron configurations from 0 to ~93250 K have been discovered. The observations in the vacuum ultraviolet establish that the positions of all the levels lying above those of the ground configuration as given in the compilation *Atomic Energy Levels*, Vol. II (1952) should be increased by 6.7 K. All but 26 faint lines of Br I have been classified. A total of 67 levels has been ascribed to the 4*s*^2^ 4p* nf configurations. It is demonstrated that the *nf* configurations exhibit almost pure pair coupling. The very regular (^3^P_2_)*nf*[5]°_11/2_ series yields for the principal ionization energy of Br I the value 95284.8 K.

## 1. Introduction

It has been part of the recent program of this laboratory to obtain improved descriptions and analyses of the first and second spectra of the heavier halogens. Work on the Br II and Cl I spectra is currently in progress, and the results for II and I II have already been published [1, 2].^1^ It is the purpose of the present paper to report the results for the first spectrum of bromine, Br I.

Iodine and bromine are very frequently used in electrodeless metal-halide lamps [3] serving as light sources for the study of rare-earth and other metallic spectra. Since the spectrum of both the metal and the halogen will appear when such a lamp is excited, it is essential that the user have available a complete and accurate description of the spectrum of the halogen in order to separate the halogen lines from those of the metal under investigation.

The most thorough study of Br I hitherto available is that contained in the excellent paper on the structure of the arc spectrum of bromine published 30 years ago by C. C. Kiess and T. L. de Bruin [4]. That paper gives, also, a summary of the investigations of Br I carried out prior to 1930. The Br I analysis by Kiess and de Bruin appears with minor revisions in the compilation “Atomic Energy Levels” [5], and when we refer to the analysis given by those authors we shall mean the form as presented in AEL.

The most significant contributions to the study of Br I to appear since 1930 are those by P. Lacroute [6], who studied the Zeeman effect in this spectrum and made some wavelength measurements in the Schumann region; and by Tolansky and Trivedi [7], who made a rather extensive study of hyperfine structure (hfs) in Br I. In the course of the latters’ analysis of the structure of 64 lines in the range 4390 to 8700 Å it was found that a number of lines exhibited hfs patterns that could not be satisfactorily explained on the basis of the interpretation of these lines as offered by Kiess and de Bruin. Without going into details, we merely state that all the anomalies encountered by Tolansky and Trivedi have been eliminated by the extended analysis of this spectrum given in the present work.

This considerably improved analysis results largely from the use of more refined apparatus, photographic emulsions, and light sources than were formerly available. The analysis rests chiefly on the 1035 spectral lines that we have recorded for Br I by using electrodeless discharge sources in the photographic air region. In comparison, Kiess and de Bruin’s line list contained only 330 lines, which is reduced to 274 when the 56 lines listed by them but not confirmed in our work are rejected.

The analysis of Br I as given in AEL has undergone a number of changes as a result of clues furnished by the greatly augmented line list. These changes can be summarized briefly as follows:

**Table:** 

	Number	
	Even	Odd
		
Original levels	39	39
Rejected	12	6
*J* or designation changed	7	17
Total real	27	33

The new wavelengths have permitted us to find 123 even and 128 odd levels for Br I compared with the 27 even and 33 odd levels listed in AEL.

## 2. Wavelength Material

(a) Vacuum region: We have observed the bromine spectrum below 2000 Å by use of a vacuum spectrograph having as dispersing element a concave grating of two-meter radius and ruled with 30,000 lines per inch directly on Pyrex. The plate factor at the normal (1920 Å) is 4.26 Å/mm, and the plateholder covers the range 0 to 2570 Å in the first order.

The light source was an end-on glass discharge tube having two side-arms set off by stopcocks and containing reservoirs of bromine and iodine. The discharge tube was affixed to the slit housing of the spectrograph by means of an ◯-ring seal, so that the slit opened directly to the discharge. The bromine vapor was retarded from entering the main spectrograph chamber by continuous pumping through a trap that entered the discharge tube just before the slit. The bromine vapor pressure in the discharge, excited in the field of 2450 Mc/s radiation from a microwave oscillator, was partly controlled by regulating the temperature at the bromine reservoir. Helium was also admitted into the discharge. The auxiliary iodine reservoir was used to provide standards [1] to supplement the impurity standards of hydrogen, helium, carbon, nitrogen, oxygen, and chlorine [8, 9]. We used EK 103a–O UV and SWR plates and measured the 1000 to 1280 Å region in the second order and the 1000 to 1650 Å region in the first order.

Table A1 in the appendix lists the 124 spectral lines in the vacuum region that we have attributed to Br I. The observed wavelengths in the first column of this table represent the weighted means of only two measurements in the second order for the range 1067 to 1120 Å and of three or more measurements in the first and second orders for the range 1120 to 1633 Å. We have also made use of measurements by W. C. Martin and C. H. Corliss of certain bromine lines that occurred on their iodine spectrograms.

The new wavelengths have made it possible to establish the ground Br I energy separation $4p^{5}{\;}^{2}\;P_{3/2}^{{}^{\circ}}{-}^{2}\;P_{1/2}^{{}^{\circ}}$ as 3685.2 K with an estimated error of ±0.3 K.^2^ Since none of the higher Br I energy levels make transitions to the ground term that yield lines in the air region, it is necessary to fix the energies of these higher levels with respect to the ground ${\;}^{2}P_{3/2}^{{}^{\circ}}$ level by use of the vacuum observations only. Our new vacuum wavelengths establish the absolute positions of all levels above the ground level to an estimated accuracy of ± 0.3 K and require that the corresponding values for Br I levels in AEL be increased by 6.7 K.

Since the relative positions of the higher Br I levels have been accurately fixed by observations in the air region, we can calculate the Br I lines in the vacuum region on an accurate and uniform scale once we have made the energy connection between the ground and excited levels through our measured vacuum wavelengths. These calculated wavelengths are given in column 2 of table A1; they agree to a better extent with the measured wavelengths over the entire range than to the ±0.01 Å we would have ventured to estimate solely on the basis of examination of the agreement of wavelengths derived from the different exposures, and especially in recognition of the inadequate standards in certain parts of the region.

(b) Air region: A preliminary list of Br I wavelengths in the photographic air region was given earlier by Tech and Corliss [10]. One will find in that paper a thorough description of the experimental procedure and the preparation of the light sources, which we summarize briefly.

**Figure 1:**
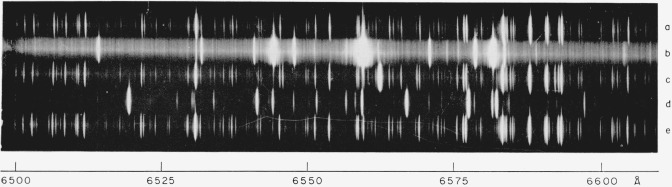
The bromine spectrum in the vicinity of *H*α (6563 Å). Exposure (b) was made with an electrodeless discharge tube containing bromine vapor excited by a microwave diathermy unit. Exposure (d) is of bromine excited by a high voltage ring discharge apparatus that enhanced Br II. The other exposures are of thorium, which served as secondary standards. H*α* occurs only in the middle thorium exposure (c).

Workers have encountered in the past two major difficulties in obtaining a light source suitable for the investigation of the bromine spectrum: (i) bromine is so chemically active a material that it attacks hot internal electrodes; and (ii) water vapor in the bromine causes the appearance of troublesome extraneous bands. Both these difficulties were eliminated in the present work by using electrodeless discharge lamps, excited by a Raytheon Microtherm microwave generator at 2450 Mc/s. For our first series of observations, we used lamps containing BeBr_2_, but for the more recent series we used lamps containing pure bromine vapor at a pressure of the order of several millimeters of mercury and dried by means of several passes through P_2_O_5_ as described in [10]. The absence of water vapor in our lamps is attested to by figure (1), displaying the bromine spectrum in the vicinity of H*α*: (6563 Å). It will be noticed that Ha does not appear at all in the bromine exposure. On the other hand, this figure shows clearly the highly objectionable Br_2_ background that occurs on spectrograms of long exposure and that reduces the effective resolving power. Furthermore, the presence of such bands often makes it difficult to decide whether certain faint lines are of atomic or molecular origin.

The spectrograms were made in the first and, wherever possible, second orders with concave gratings having 7,500, 15,000, and 30,000, lines per inch, and mounted in parallel light (Wadsworth mounting), giving first order plate factors of 10, 5, and 2.4 Å/mm respectively. All the spectrograms bore exposures to an electrodeless lamp containing thorium or iron, or to the iron arc, to serve as secondary standards in the determination of the bromine wavelengths. The plates were reduced either by linear interpolation and a correction curve, or by putting the micrometer readings of the lines on punched cards and carrying out the reduction by use of an IBM 704 computer, which adjusted all wavelengths to fit a fourth degree equation determined by least squares.

Table A2 contains the wavelengths, intensities, wave numbers, and classifications of 1035 Br I lines observed in the range 3325 to 12810 Å. Except for the weaker lines in the region 4264 to 5721 Å, and except for very faint lines over the whole range, the wavelengths in the table represent the weighted means of from two to eight measurements. Because of the inherent broadness of many of the lines, partly as a result of unresolved hyperfine structure, and because the weaker lines were only measured once or twice, the accuracy of the wavelengths is not as great as one would wish. The estimated error of the wavelengths according to spectral region is:

**Table:** 

λ	Error
3325–4300 Å	± 0.005 Å
4300–7400 Å	± 0.02 Å
7400–11000 Å	± 0.03 Å
11000–12900 Å	± 0.06 Å

Lines in table A2 whose wavelengths are connected by braces are individually measured components of resolved or partially resolved hyperfine structure. Most of the Br I lines exhibiting such wide, resolved hfs patterns result from transitions involving the 4*p*^4^(^1^D_2_)5*s* ^2^D_5/2_, ^2^D_3/2_ levels or the 4*p*^4^(^3^P_2_)5*s* ^4^P_5/2_ level, a clue that was very useful in the analysis.

The character of certain lines is indicated by inserting the following symbols after the intensity of the line:
calmost resolved hyperfine structuredunresolved double linehhazy, diffuseHvery hazy, very diffusesshaded to shorter wavelengthswwideWvery wide.

The numbers in column 2 of the table are visual estimates of the intensities on an approximately linear scale where the faintest line is assigned an intensity 1. It must be emphasized that these relative intensities are valid only over small wavelength ranges, and no attempt has been made to adjust the values to take into account the varying sensitivity of the photographic emulsions.

Lines of wavelength greater than 11316 Å were measured also on the infrared recordings described in the next section. For comparison, the intensities derived from these recordings are listed in parentheses following the photographic intensities for these lines in table A2. The symbol (*m*) indicates that the line was masked on the recorder charts either by a standard line or by a bromine line of another order.

Table A2 incorporates several improvements over our earlier list of wavelengths. Additional chlorine lines were deleted by consulting the unpublished chlorine line list of Corliss. Chlorine accounted for nearly all the impurity lines found on our spectrograms. Furthermore, several faint lines, measured only once, especially in the region 4725 to 4825 Å have been ascribed to Br_2_ and removed from the list. The line at 4358.33 Å has been attributed to Hg and probably originated in the scattered light entering the slit from fluorescent illumination in our source room.

More importantly, additional plates have been measured in parts of the spectrum above 7400 Å. This has not only yielded a number of new lines in this region, but also has allowed us to correct a small systematic error of uncertain origin that was present in some of the earlier measurements in this region. The new wavelengths from 7400 to 9700 Å differ, if at all, by only about +0.01 Å from the earlier values. Above 9700 Å the correction averages somewhat less than +0.04 Å.

Finally, W. F. Meggers and R. Zalubas have generously provided us with some Br I wavelengths that they had measured on their Yb and Th spectrograms, respectively. These measurements have also been averaged into our new mean wavelengths.

(c) Lead sulfide region: The infrared description of Br I was extended to 24100 Å by the use of recording infrared spectrometers. The wavelengths of 94 new lines were measured by the author from radiometric records in the form of recorder charts generously supplied expressly for this investigation by C. J. Humphreys at the Naval Ordnance Laboratory, Corona, and by E. D. Tidwell at the Bureau. In both laboratories electrodeless discharge lamps, containing pure bromine and excited by 2450- megacycle microwave diathermy units, served as light source. The records made at Corona were obtained by scanning the spectrum with the highresolution grating spectrometer designed and built at the National Bureau of Standards. This apparatus has been described by Humphreys and Kostkowski [11]. The bromine wavelengths were measured on these records by linear interpolation between krypton standards that were introduced by reflection from back of the various filters used to sort out the higher order bromine lines. This procedure does not yield the most precise wavelengths, but it was entirely satisfactory for our purposes. For nearly all lines, the departure of the measured wavelengths from those calculated by the combination principle was less than 1 Å. All lines (except 19317 Å and 21093 Å) of intensity 40 and above in the region beyond 19000 Å were also measured on the tracings made by Tidwell, who used the infrared spectrometer at NBS. These wavelengths could be measured with precision by interpolation between the white-light fringes of a Fabry-Perot interferometer that were recorded simultaneously with the bromine spectrum. This technique, as well as the spectrometer apparatus, has been described by Plyler, Blaine, and Tidwell [12].

With the exception of a single chlorine line,^3^ every line established from these records can be classified as a transition between Br I levels whose values have been accurately determined from observations in the photographic air region. Precise wavelengths for these lines can therefore be calculated by use of the combination principle. It is these calculated wavelengths that are listed in table A3, which includes four lines above 12000 Å that were also observed photographically.

The relative intensities given in the second column of this table express, in tenths of an inch, the heights of the recorded line profiles above the continuum. One of the tracings furnished by Humphreys was selected to provide the standard scale to which the intensities of lines measured on other tracings would be referred. As the spectrum was being scanned, lines of intensity greater than 85 were attenuated by manual adjustment of a resistance network introduced in the input of the amplifier. The measured heights of these lines were multiplied by the known attenuation factors corresponding to the various resistance box dial settings. Since the amplifier is linear within the limits of loading, and the response of the detector is almost flat within the range covered, the relative intensities are considered to be very reliable over intervals of several hundred angstroms, but not over the entire region covered, inasmuch as no correction was made for the spectral response of the detector or angular distribution of energy from the grating.

In table 1 are displayed the wave numbers and intensities (in parentheses) of certain important multiplets observed in the radiometric region. Observed multiplets that helped to establish the important (^3^P)6*s* ^4^P levels are shown in table 2. In both these tables, transitions allowed by the selection rules, but unobserved, are indicated by horizontal bars.

## 3. Term Structure of Br I

The unexcited state of the neutral bromine atom is characterized by the electron configuration 4*s*^2^ 4*p*^5^, which yields an inverted ^2^P term of odd parity. From the vacuum ultraviolet observations discussed above, the position of 4*s*^2^4*p*^5^ ${\;}^{2}P_{1/2}^{{}^{\circ}}$ is found to be 3685 K, relative to ${\;}^{2}P_{3/2}^{{}^{\circ}}$ taken as zero. The 4*s*4*p*^6^ configuration gives one even level, ^2^S_1/2_, while all other known Br I levels arise from excited configurations of the type 4*s*^2^ 4*p*^4^ *nl*, where *l* stands for the letters *s, p*, *d, f* indicating the orbital angular momentum quantum number, and *n* is the principal quantum number. It is convenient to refer to 4*s*^2^ 4*p*^4^ *nl* configurations simply as *nl* configurations. The terms that are expected theoretically for each of these configurations are displayed in table 3, built in the *LS* scheme. The *nl* levels are based on, and can be derived from, the parent ^3^P_2,1,0_^1^D_2_ and ^l^S_0_ terms of the 4*p*^4^ configuration of the ion, Br^+^. The observed distribution of the levels in each of the Br I *nl* configurations indicates that the coupling energies of the 4*p*^4^ core dominate the structure of the Br I levels. With just a few exceptions the coordination of Br I levels to specific components of the limiting 4*p*^4^ terms is unambiguous. When discussing Br I levels in text, this limit is stated explicitly. However, in our tables of observed lines, the levels involved in the classification of the lines are written in an abbreviated notation, which affixes no prime, one prime, and two primes to the symbol for *l*-value to designate that the level is based on the ^3^P_2_, ^3^P_1_, and ^3^P_0_ parent, respectively. For levels based on ^1^D_2_ and ^1^S_0_, the parent is stated explicitly.

The positions of the Br II 4*p*^4^ levels have been accurately determined by Martin and Tech [14], who established the 4*p*^4 3^P_2, 1, 0_ levels from new observations of the vacuum ultraviolet spectrum and the ^1^D_2_ and ^1^S_0_ levels from observations in the air region of the “forbidden” magnetic dipole transitions, 4*p*^4 3^P_2_–4*p*^4 1^D_2_ and 4*p*^4 3^P_1_ – 4*p*^4 1^S_0_. Since the distribution of these parent levels manifests itself in the structure of all the Br I configurations, the positions of the observed parent levels are repeated here in table 4, together with a slightly different fit to intermediate coupling theory from that given earlier.

The spin-orbit interaction energies in the 4*p*^4^ configuration may be obtained by reversing the sign of *ζ_p_* in the matrices for *p*^2^ given in *The Theory of Atomic Spectra* [15, p. 268]. The diagonal electrostatic energies to be added are^4^

$$\begin{array}{lll} {\;}^{3}P & : & 6F_{0}—15F_{2}\\ {\;}^{1}D & : & 6F_{0}—9F_{2}\\ {\;}^{1}S & : & 6F_{0} \end{array}$$

where F_0_=F_0_(4*p*, 4*p*) and $F_{2}=\frac{1}{25}F^{2}(4p,4p)$. The choice of coupling parameters in the previous calculation of the distribution of Br II 4*p*^4^ levels [14] resulted in the fit shown in the third column of table 4. Since these discrepancies reappear to some extent in the calculation of Br I 4*p*^4^5*s* levels presented below, it has seemed worthwhile to recalculate the parents in order to demonstrate that, with just a small change in parameters, the calculation of Br II 4*p*^4 1^S_0_ can be improved without appreciably affecting the fit for the ^3^P_2,1,0_ levels. The new fit, shown in the last column of table 4, is a strict least squares fit to the four levels other than ^1^D_2_. It predicts the total energy spread of the configuration exactly, and emphasizes that the larger discrepancy for 4*p*^4 1^D_2_ may actually be due to a perturbation by a higher level, since the discrepancy is in the right direction.

Tables 5 and 6 contain all the energy levels of the neutral bromine atom that we have been able to find by using the new wavelengths. The tables are arranged so as to display clearly the level parentage, configuration, and series. For each level are given the energy (*E*) relative to 4*p*^5^ ${\;}^{2}P_{3/2}^{{}^{\circ}}$ taken as zero, the absolute energy value (*T*) measured from the principal ionization limit, i.e., from Br II 4*p*^4 3^P_2_, and the effective quantum number (n*) calculated according to n*=√(R/T), where *R* is the Rydberg constant for bromine, 109736.56 K. The designations of the levels, usually in both the *LS* scheme and the *J_c_l* scheme, are given just to the left of the first member of each series and hold for all other levels of the series. In all except the *nf* configurations one might say that the coupling is “midway between” the *LS, J_c_j*, and *J_c_l* coupling schemes. The almost pure pair-coupling in the *nf* configurations permits positive assignment of *J_c_l* symbols to the *nf* levels. All other levels are consistently referred to in terms of the adopted *LS* symbols, for reasons of tradition, of the general familiarity of this notation, and of the lack of any really satisfactory solution to the vexing problem of notation for intermediate couplings.

**Figure 2:**
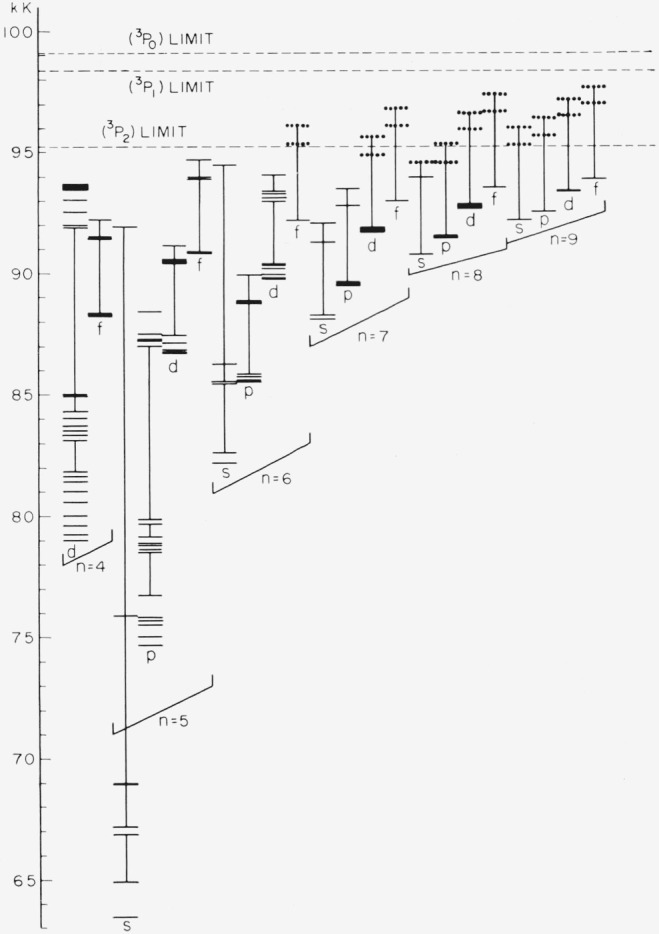
The distribution of *Br I* energy levels for n = 4 to n = 9, as discussed in the text.

Figure 2 shows the distribution of *ns, up, nd, nf* levels through *n*=9. The small horizontal bars in each *nl* column represent the position of one *or more* levels. The scale of the diagram does not allow an individual representation for each level. The vertical lines in each column separate groups of levels having different parents, the lowest group of levels in each column being based on the (^3^P_2_) parent. It should be remembered that when a level group appears to be intersected by a vertical line (by virtue of being met from above and from below by such a line), then there is a change of parent in either going to the next higher or the next lower group. The separation of the level groups for each *nl* clearly reflects the energy differences of the parent limits shown by the dashed lines in the figure. Thus, except for absolute energy position, the levels of many *nl* sets have the same appearance in this figure: three groups of levels held apart by the parent differences. This “building-block” character is well observed at *4f*, 5*f*, *6p*, and *7s*, for example, and can be expected to hold at all high *n.* The dotted level positions represent some predicted, but unobserved, levels based on (^3^P_1,0_). It is seen that the only unobserved levels based on parents other than (^3^P_2_) and lying below the (^3^P_2_) limit are those of (^3^P_1_) 8*p*, (^3^P_1_)7*d*, and (^3^P_0_)8*s*. The other dotted positions fall above the (^3^P_2_) limit, and the atoms excited to these levels can be expected to autoionize.

It is convenient at this point to discuss the extent to which significance may be attached to the symbols used in this paper to describe the energy levels. The *LS* symbols are based on an examination of the intensities in the transition arrays, with symbols being assigned in such a way that the intensity relations most nearly conform to the well- known *LS* rules. The arrays used were those containing levels of low principal quantum number *n*, the assignments being retained along series without regard to intensities. *LS* symbols assigned to levels in this way will not necessarily survive if the actual energy matrices in the *LS* scheme are diagonalized and the eigenvectors found, because when the coupling strongly departs from the *LS* scheme, the intensities then being governed more by, say, the *J_c_l* rules, the *LS* symbols to which one is led depend more on *J_c_* and K and will not with assurance correspond to the major *LS* contribution to the composition of the levels. A preliminary rough diagonalization of the Br I 5*p* energy matrices suggests, for example, that the “percentage-method” would require the $\left({\;}^{3}P_{2}\right)5p{\;}^{4}D_{5/2}^{{}^{\circ}}$ and $\left({\;}^{3}P_{1}\right)5p{\;}^{2}D_{5/2}^{{}^{\circ}}$ levels to have their designations interchanged, whereas the intensities would not suggest this. In any case, the departure from *LS* coupling is so great that *L* and *S* are not good quantum numbers anyway, making meaningless any involved procedure for naming the levels. In the absence of reliable eigenvectors, the “intensity-method” seems to be the most attractive. The *np LS* assignments in Br I turn out to correspond exactly to those in FI [16], but are quite different from those adopted for the 11 *np* levels by Kiess and Corliss [1], who assigned *LS* symbols by forming a one-to-one best correspondence of Laudé *g*-factors to observed *g*-factors. For small departures from pure *LS* coupling, all three methods should result in the same designations.

Ideally, one should like to designate observed levels in the notation of the coupling scheme in which the energy matrices are already most diagonal, that is, in which there is the least nondiagonal contribution to the energies (eigenvalues). In this sense, the Br I *nf* configurations are very nearly pair-coupled, and *J_c_l* symbols should certainly be used for the *nf* levels. For the sake of consistency, *J_c_l* symbols have also been assigned to the other Br I levels, as alternatives to the *LS* symbols. This is justifiable on the grounds that for *ns* the choice of *J_c_l* or *J_c_j* notation is arbitrary owing to the identity of the matrices built in the two schemes, while for *np* and *nd* the notation could be equally well *J_c_l* or *J_c_j* since the coupling is intermediate. In these cases only exact calculation would allow us to decide in favor of one or the other scheme, and this would still be difficult for *nd* because of the sizeable perturbations that afflict the *nd* configurations. The *J_c_l* designations for the Br I *np* levels have been chosen on the basis of approximate calculations by using the diagonal *J_c_l* energy expressions. Diagonal expressions are less valid for *nd*, so that only general considerations of level positions could be invoked in the assignment of *J_c_l* notation, the primary emphasis being placed on the intensities of (^3^P_2_)4*d–* (^3^P_2_)*nf* transitions for the (^3^P_2_)4*d* designations and 5*p–*4*d*, 5*p–*5*d* transitions for the other *nd* designations.

The main features of *J_c_l* coupling and the theoretical treatment of electronic configurations exhibiting this type of coupling are discussed at length by Minnhagen [17] in his fine paper on Ar II. The theoretical treatment is based upon the general formulas of Möller [18], who calculated the *p*^4^*l* matrices of electrostatic energy in the *J_c_l* scheme. The corresponding matrices of spin-orbit interaction have recently been provided by Källén and appear in the appendix of reference [19].

A pair-, or *J_c_l* coupled configuration is characterized by (i) a domination by the core coupling energies, and (ii) an electrostatic interaction of the outer electron with the core that is stronger than the spin coupling of this electron. The second requirement means, in the case of the 4*p*^4^*nl* configurations of Br I, that the contribution of F^2^(4*p*, *nl*) to the level energies must be greater than that of *ζ_nl_* and the G*^k^*(4*p*, *nl*). The notation used for a level in a pair- coupled configuration is that suggested by Racah [20], in which the level is designated as ()*nl* [*K*]*_J_*, where the parent is placed in the parentheses, and where *K*, the intermediate quantum number, is the quantized resultant of the coupling of the total angular momentum *J_c_* of the parent ion with the orbital angular momentum of the external electron. The coupling of *K* with the spin of this electron gives the *J*-value of the level. Each *K*-value thus has two *J*-values associated with it, *J*=*K*±½. For Br I, *J_c_* can take the values 2, 2, 1, 0, and 0, corresponding to the five levels of the ground configuration 4*p*^4^ in the ion. The selection rules governing electric dipole transitions between *J_c_l* coupled levels are

$$\Delta J_{c}=0;\;\Delta K=0,\pm 1;\;\Delta l=\pm 1;\;\text{and}\;\Delta J=0,\;\pm 1.$$

The last two are, of course, independent of coupling, but the first two are easily violated when departures from pure pair coupling occur, because the energy matrices are not diagonal in *J_c_* or *K*.

Owing to the departures in Br I from any pure coupling scheme, the single level eigenfunctions of any such scheme are mixed. *L*, *S, J_c_*, and *K* are not very good quantum numbers, and the only restrictions on transitions seem to be those on parity and Δ*J.*

## 4. Ionization Limits

A very sharp determination of the principal ionization limit of the neutral bromine atom is possible now that the ${(}^{3}P_{2})nf{\left[5\right]}_{11/2}^{{}^{\circ}}$ series has been observed. This series is found not only empirically to provide the best value for the limit; it is also the series one might expect on theoretical grounds to provide such a best value. The theoretical criterion one should use in the selection of the least perturbed series for the limit determination is that the effect of nondiagonal matrix elements connecting the levels to other levels of common *l* and J value shall be very small or zero. In general, this criterion restricts consideration to series of levels with high *l* and *J* values.

Since no level with *J*=11/2 can occur in the *np* configurations, there will be no perturbation of the ${(}^{3}P_{2})nf{\left[5\right]}_{11/2}^{{}^{\circ}}$ series by levels of those configurations. Indeed, the only nondiagonal element that makes a connection to this series is the ζ_4_*_p_* element between ${(}^{3}P_{2}){\left[5\right]}_{11/2}^{{}^{\circ}}$ and ${(}^{1}D_{2}){\left[5\right]}_{11/2}^{{}^{\circ}}$, but the latter *nf* series is well above the (^3^P_2_) ionization limit, the lowest member of the series, ${(}^{1}D_{2})4f{\left[5\right]}_{11/2}^{{}^{\circ}}$, being already more than 5000 K above the (^3^P_2_) limit.

The actual determination of the limit from the ${(}^{3}P_{2})nf{\left[5\right]}_{11/2}^{{}^{\circ}}$ series has been carried out graphically. The enective (principal) quantum number, n*, of a series member is defined by the relation *T=R/*(n*)^2^, where *T* is the position of the level measured from the series limit and *R* is the Rydberg constant, which for bromine equals 109736.56 K. If the series is Ritzian, then n*=n–α–βT_n_, where *n* is the running principal quantum number and *α* and *β* are constants for a given series. The position of the series limit is taken as that value for which the plot of (n—n*) versus *T_n_* yields the best straight line. The quantity (n–n*) defines the quantum defect, *δ=a+βT_n_.* In practice it is usually more convenient to plot *T_n_* against (*C–δ*), where *C* is some integer for the series such that (*C–δ*) is the part of n* to the right of the decimal.

**Figure 3:**
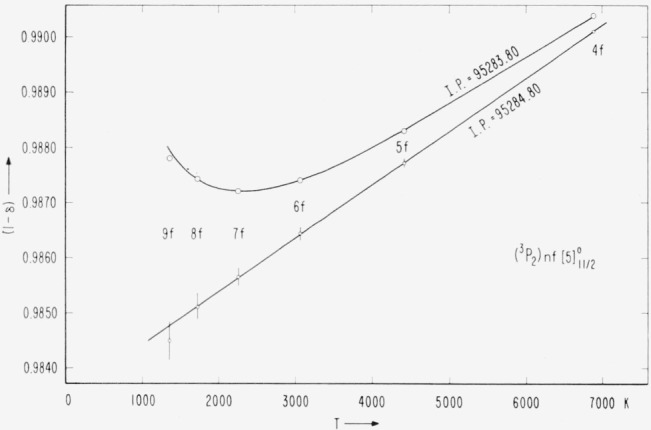
The $\left({\;}^{3}P_{2}\right)nf{\left[5\right]}_{11/2}^{{}^{\circ}}$ series plotted for two different values of the limit. The upper curve results from changing the value of the Br I ionization potential by only 1.00 K from the value adopted for Br I on the basis of the lower plot. The vertical bars in the lower plot show the effect on (1–*δ*) of a change of ± 0.1 K in the limit.

Figure 3 shows that a very nearly straight line results if the 4*p*^4^(^3^P_2_) limit is taken to be 95284.80 K above the 4*p*^5^ ${\;}^{2}P_{3/2}^{{}^{\circ}}$ ground level of Br I. The vertical lines through the points represent the deviation of (1 – *δ*) resulting from a change in the limit of ±0.10 K. If the adopted series limit is reduced by only one kayser, we obtain the unsatisfactory and unreasonable behavior of this series depicted by the second plot in figure 3.

With the adopted *IP*= 95284.80 K, we have made a least squares calculation of the constants *α* and *β* by using the first five series members. How well these constants reproduce the series members can be seen from table 7. The position of $\left({\;}^{3}P_{2}\right)9f{\left[5\right]}_{11/2}^{{}^{\circ}}$ would have to be raised by 0.09 K in order to bring its point in figure 3 onto the line. This is certainly within the experimental error, since the level has been established on the basis of two measurements of a wide, hazy line at 7495.46 Å. A change of 0.05 Å in this wavelength would bring the corresponding level into line.

The value of 95284.80 K for the principal ionization energy is well supported by other series. The $\left({\;}^{3}P_{2}\right)nf{\left[5\right]}_{9/2}^{{}^{\circ}}$ series gives an identical limit, although the point for 9*f* again falls about 0.1 K below the line, and again the spectral line establishing the level is wide and faint (7783.44 Å). With this limit, the plots of the higher members of the (^3^*P*_2_)*nd* ^4^F_9/2_ and $\left({\;}^{3}P_{2}\right)np{\;}^{4}D_{7/2}^{{}^{\circ}}$ series are also very nearly linear, while the greater deviation from linearity in the first few members of each of these series is a result of the changing coupling conditions along the series.

It may be pointed out that, although the apparent depression of $\left({\;}^{3}P_{2}\right)9f{\left[5\right]}_{11/2}^{{}^{\circ}}$ and $\left({\;}^{3}P_{2}\right)9f{\left[5\right]}_{9/2}^{{}^{\circ}}$ is within the experimental uncertainty, the depression of these levels might more interestingly be a result of an inter-ionic Stark effect. The conditions at 9*f* are ripe for such an effect, and the character of the observed lines from these levels are certainly suggestive of Stark broadening. Such an interpretation is strengthened by the fact that the lines $\left({\;}^{3}P_{2}\right)4d\;4F_{9/2}-\left({\;}^{3}P_{2}\right)7f{\left[5\right]}_{11/2}^{{}^{\circ}}$ and $\left({\;}^{3}P_{2}\right)4d\;4F_{7/2}-\left({\;}^{3}P_{2}\right)7f{\left[5\right]}_{9/2}^{{}^{\circ}}$, as well as 9 out of 13 of the observed (^3^P_2_)4*d–*(^3^P_2_)8*f* lines have been recorded as hazy or wide. The regularity of the $\left({\;}^{3}P_{2}\right)nf{\left[5\right]}_{11/2}^{{}^{\circ}}$ series used to determine the ionization limit leaves no doubt, however, that the determination is an excellent one, accurate to probably ±0.15 K relative to the levels used in the determination, even allowing a small Stark effect on the *n* = 7, 8 members. Such an effect would have no more influence than the normal experimental error on the limit determination.

It seems reasonable therefore to adopt 95284.8 ± 0.5 K as the position of Br II 4*p*^4 3^P_2_ above the ground level of Br I. We have included in the stated error the estimated accuracy of the absolute positions of the higher levels. With the conversion factor 12395×10^−8^ ev/K as adopted in AEL, we obtain 11.811 ev for the principal ionization energy of Br I. If we use a more recent value [25] for the conversion factor, 8066.03 ±0.14 K/ev, we obtain 11.8131 ±0.0003 ev for this ionization energy.

Although several Br I series having three members and based on the higher (^3^P_1,0_) are known, none of them is unperturbed. More accurate values for the positions of the higher limits can be found by combilling the above (^3^P_2_) limit with the positions of the Br II 4*p*^4^ levels as given by Martin and Tech [14]. It is estimated that these Br II levels are accurate to ±0.5K. The five limits are then as follows:

$$\begin{array}{l} \left({\;}^{3}P_{2}\right)\;:\;95284.8\\ \left({\;}^{3}P_{1}\right)\;:\;98421.2\\ \left({\;}^{3}P_{0}\right)\;:\;99122.3\\ \left({\;}^{1}D_{2}\right)\;:\;107373.9\\ \left({\;}^{1}S_{0}\right)\;:\;123151.9 \end{array}$$

These are the limits that were used in the calculations of n* for the Br I levels.

The (^3^P_2_) limit found in this investigation is 265 K lower than that given in AEL, Vol. II. It is noteworthy that Catalán and Rico predicted a value of 95300 K for this limit on the basis of their study of the Ga I–Rb I sequence [21].

## 5. Levels of Even Parity

(a) 4*p*^4^*ns:* Except for (^3^P_0_) 8*s*^2^P_½_, which is calculated to fall around 94650 K, every (^3^P) *ns* level through 11*s* that is predicted to lie below the (^3^P_2_) limit is now known. The (^3^P)5*s* group and the two (^3^P_2_)7*s* levels were known before. With the exception of (^3^P_2_)8*s*^4^P_5/2_, assigned to the 6*d* configuration in AEL, all other *ns* levels are new. The (^3^P)7*s* ^2^P_3/2, 1/2_ levels listed in AEL are spurious.

The most important of the new (^3^P)*ns* levels are those belonging to (^3^P)6*s*. The strongest lines from these levels fall in the lead-sulfide and vacuum regions, but it has been possible to determine all the levels accurately from weaker lines observed in the photographic air region. Catalán and Rico [21], who made a graphical study of the Ga I–Rb I sequence by means of term values and the constants *α* and *β* appearing in the expression defining the quantum defect, predicted the positions of the then unknown (^3^P_2_)6*s*^4^P_5/2_ and (^3^P_1_)6*s*^2^P_3/2_ levels as 82300 K and 85649 K, respectively, which are remarkably close to their observed positions 82236 K and 85435 K. Catalán and Rico also came to the conclusion that (^3^P_2_)5*s*^2^P_3/2_ at 67184 K was considerably perturbed, its “unperturbed” position being 67969 K. However, the results of certain calculations, presented below, do not support such a conclusion. It seems that the differing coupling conditions in the elements of the sequence studied by Catalán and Rico are responsible for the appearance that the 67184 level was perturbed.

In both the 5*s* and 6*s* configurations the ^2^D term based on the (^1^D_2_) parent falls below the (^3^P_2_) limit. The term belonging to 5*s* is discussed below. The (^1^D_2_)6*s* ^2^D_3/2_ level, 94470.7 K, has been established on the basis of only one line, identified as 4*p*^5 2^P*°*_½_−(^1^D_2_)6*s*^2^D_3/2_, at 1101.498 Å in the vacuum ultraviolet. This line was long regarded by us as belonging to Br II, but its intensity and the fact that it establishes the (^1^D_2_)6*s* ^2^D_3/2_ level within just a few kaysers of its predicted position (table 11), make us believe that our identification is almost certainly correct. One then wonders whether the unclassified line at 5139 Å might not be $\left({\;}^{3}P_{2}\right)5p{\;}^{4}P_{3/2}^{{}^{\circ}}-\left({\;}^{1}D_{2}\right)6s^{2}D_{5/2}$, placing the latter level at 94460.97 K.

The only *ns* configuration in which the ^2^S_½_ level based on the (^1^S_0_) parent falls below the (^3^P_2_) parent is 5*s.* The results of calculations presented below give ~91940 K for the position of (^1^S_0_)5*s* ^2^S_½_. There are three eligible levels in this vicinity that have *J* = 1/2, at 91691, 91824, and 91938 K. (For intensity reasons and on the basis of calculations, the level at 92090 K has been assigned to (^3^P_0_)7*s* ^2^P_½_.) Since the 91938 level agrees so well with the predicted position, we have interpreted this level as the (^1^S_0_)5*s* ^2^S_½_. The other two levels have been assigned to the (^3^P_2_)7*d* configuration. It is felt that this interpretation of the two lower levels also leads to the most consistent *nd* series. An observed *g*-factor for the level at 91938 K will be required in order convince us that this interpretation is correct, however.

The (^3^P)5*s* levels were all known before, but a curious circumstance led Kiess and de Bruin to adopt incorrect (^1^D_2_^1^S_0_)5*s* and (^1^D_2_^1^S_0_)5*p* levels. This caused some confusion in the preparation of AEL about the correct order of the (^1^D_2_) and (^1^S_0_) limits in Br II. The 5*s* levels in question, adjusted to our energy scale, are:

**Table:** 

	This work	Ref. [1]
		
(^1^D_2_) ^2^D_5/2_	75890.3 (−18.2)	$\begin{array}{cc} \begin{array}{l} 77330.8\\ \left(+18.2\right)\\ 77312.6\\ 75908.6 \end{array}] & \Delta =1422.2\;K \end{array}$
(^1^D_2_) ^2^D_3/2_	75908.5	
(^1^S_0_)^2^S_1/2_	91938.0	

It is seen that Kiess and de Bruin’s ^2^S_1/2_ level is real and corresponds to our ^2^D_3/2_. Although their ^2^D term is inverted with respect to ours, it has the *same* separation, 18.2 K. This led to a qualitatively correct interpretation of many lines in Kiess and de Bruin’s work, even though most of their (^1^D_2_)5*s*, 5*p* levels are spurious. Both levels of their (^1^S_0_)5*p* ^2^P° term are real but are actually based on the ^3^P parent. Since Kiess and de Bruin established the (^1^D_2_)5*p* levels on the basis of strong lines having the 18.2 K separation of their spurious (^1^D_2_)5*s* levels, all (except ${\;}^{2}P_{3/2}^{{}^{\circ}}$) of their (^1^D_2_)5*p* levels are spurious as well, and are all (except ${\;}^{2}P_{1/2}^{{}^{\circ}}$) raised above real levels by an amount corresponding to the difference Δ = 1422.2 K. The reason that this fortuitous system could have appeared so convincing—the transition array is displayed in reference [4]—is that Br I is a prime example of a spectrum in which chance numerical relationships occur among the levels and give support to gross errors in the analysis. It is through such relationships that additional errors can easily be generated; in this case they also account for some of the incorrect *J*-values given by Kiess and de Bruin.^5^

The energy levels of the Br I 4*p*^4^5s configuration have been calculated by diagonalizing the second- order energy matrices built in the *LS* scheme. The matrices of spin-orbit interaction are obtained from those of *p*^2^*s* on page 268 of TAS by reversing the sign of the spin-orbit integral associated with the core, *ζ_p_* The diagonal electrostatic energies to be added to the spin-orbit matrices are as follows:

$$\begin{array}{l} {\;}^{4}P\;:\;F_{0}-15F_{2}-3G_{1}\\ {\;}^{2}P\;:\;F_{0}-15F_{2}\\ {\;}^{2}D\;:\;F_{0}-9F_{2}-2G_{1}\\ {\;}^{2}S\;:\;F_{0}-2G_{1} \end{array}$$

where F_2_= (1/25)F^2^(4*p*, 4*p*), G_1_=(1/3)G^1^(4*p*, 5*s*), and F_0_ = 6F_0_(4*p*, 4*p*)+4F_0_(4*p*, 5*s*).

In the first diagonalization we adopted the values ζ_4_*_p_* and F_2_ found above for the 4*p*^4^ configuration of parent levels. In order to compensate approximately for the discrepancy in the calculated position of the (^1^D_2_) parent, we have added an electrostatic correction term *C* = −150 K to the diagonal energy expression above for the ^2^D term, built on (^1^D_2_). A least squares fit, holding *C* constant, was then made to the 5*s*-levels other than ^2^D_5/2_,_3/2_, since this term is known to be perturbed. The calculation yielded G_1_ = 733.8. The results of the fit are given in table 8 and show that the ^2^D levels have apparently been pushed down by about 300 K. It is for this reason that any attempt to determine physically meaningful parameters from the diagonal-sum rule necessarily leads to failure. In fact, this method yields an imaginary value for ζ_4_*_p_* and a negative G_1_.

In order to obtain better eigenvectors for a calculation of *g*-factors, we attempted to improve the parametric representation of the 5*s* levels by making a strict least squares fit to the levels other than ^2^D_5/2,3/2_ and by allowing all parameters (except *C*) to vary. The main effect on the parameters was a reduction of the value of *ζ*_4_*_p_*. The fit is shown in table 8. By means of the eigenfunction expansion coefficients (table 9) obtained in this calculation, the theoretical *g*-factors can be calculated. These are compared in table 10 with the observed *g*-factors. The *g*-factors for these 5*s* levels are derived from measurements of the Zeeman patterns we obtained by exposing to an electrodeless lamp placed in a field of about 37000 oersteds. Since our exposure times were not long for these preliminary exposures, only the stronger Br I lines were recorded. These included most of the lines in the 5*s*-5*p* transition array, however. Also averaged with our own measurements were values generously furnished us by W. F. Meggers, who had measured some Br I Zeeman patterns occurring on his ytterbium spectrograms. The final observed *g*-factors should be accurate to better than 0.015. The agreement between observed and calculated values is seen to be very good. The observed *g*-sums also agree well with the theoretical sums. It should be noticed that the *LS* levels ^2^P_3/2_ and ^4^P_3/2_ are thoroughly mixed.

It is gratifying that two effects predictable on the basis of the perturbation of the (^1^D_2_)5*s* ^2^D levels are actually observed. The first concerns the group of moderately strong lines in the region 7975 to 8035 Å. These lines all show resolved hyperfine structure and are (^1^D_2_)5*s* ^2^D–(^3^P_2_)4*f* transitions, which point to apparent violations of the selection rule on the orbital angular momentum of the external electron, Δ*l* = ±1. A reasonable interpretation of these lines is that (^1^D_2_)5s ^2^D contains an admixture of (^3^P_2_)4*d* eigenfunctions. Probably each of the (^1^*D*_2_)5*s* ^2^D levels is mixed with both of the two possible (^3^P_2_)4*d* levels of the same *J*-value, but the 5*s* ^2^D_5/2_×4*d* ^4^D_5/2_ and 5*s* ^2^D_3/2_×4*d* ^4^D_3/2_ interactions are perhaps the stronger because of the proximity of the levels. This would explain both the Δ*l*=3 transitions and the fact that the (^1^D_2_)5*s* ^2^D levels have apparently been pushed downward. The situation is actually more complicated than this, because the strong (^1^D_2_)5*s* ^2^D –(^3^P_2_)4*f*[2]° combinations are caused partly by the additional (^1^D_2_)5*p* ^2^D°× (^3^P_2_)4*f*[2]° interaction.

The (^3^P)5*s* levels also combine weakly with (^3^P)*nf* levels, partly because of small 5*s×*4*d* interactions and also through the (^1^D_2_)5*s* ^2^D levels.

The other effect of the (^1^D_2_)5*s ×* (^3^P_2_)4*d* interaction is a reciprocal one to the above and concerns the unexpected intensity of the (^3^P_2_)4*d –* (^1^D_2_)5*p* transitions, particularly the line at 10840 Å, resulting from the transition $\left({\;}^{3}P_{2}\right)4d^{4}D_{5/2}–\left({\;}^{1}D_{2}\right)5p^{2}D_{5/2}^{{}^{\circ}}$.

Already at 5*s* the separation of the level groups according to parentage is obvious. For *n*≧6 the dominance of *ζ*_4_*_p_* should permit the calculation and identification of the *ns* levels by use of simplified matrices for *J_c_j* or *J_c_l* coupling. Since these are identical in the two schemes for *l*=0, the coupling might be called *J_c_s* coupling. The complete *J_c_s* matrices for *p*^4^*s* have been given by Minnhagen [19].^6^

As was the case in II, the relative magnitudes of the *J_c_s* matrix elements permit a simplification such that the *p*^4^*s* levels in Br I may be calculated by use of the following approximate expressions:

**Figure:**
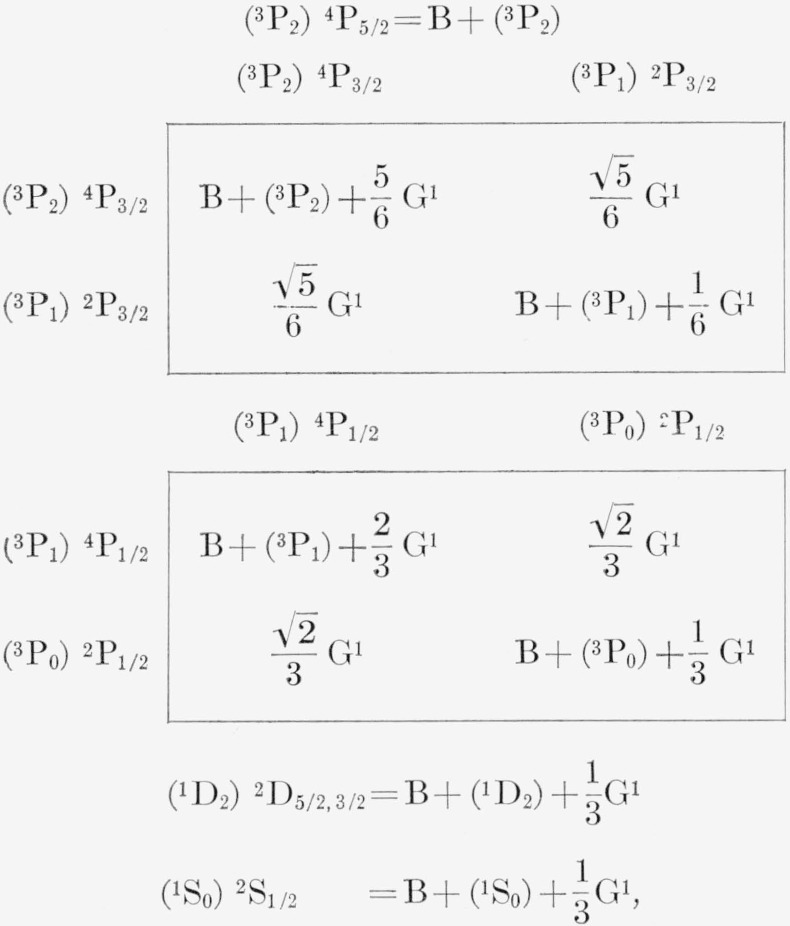


where the parent symbols stand for the observed values: (^3^P_2_)=0, (^3^P_1_) = 3136, (^3^P_0_)=3838, (^1^D_2_) = 12089, and (^1^S_0_) =27867 K. *LS* symbols are given the Br I *p*^4^*s* levels in the company of *J_c_s* energy expressions, but the *J_c_l* correspondence can be found in table 6.

The *LS* eigenvectors for (^3^P)5*s* show that the *J*=1/2 levels are relatively pure in the *LS* scheme while the *J*=3/2 are thoroughly mixed. As might be expected, the calculation of (^3^P) 5*s* from the above *J_c_s* expressions gives just the opposite results, i.e., the *J*=1/2 levels are thoroughly mixed in the *J_c_s* scheme while the *J*=3/2 levels are pure. This same behavior was found by Minnhagen in II. The large electrostatic interaction of the *J*=1/2 levels is also present for *n*≥6 and reveals itself through a larger nondiagonal contribution to the energy than for the (^3^P) *J*=3/2 levels. The comparison of observed 6*s* and *7s* levels with those calculated from the *J_c_s* expressions is given in table 11. Since the agreement is excellent for a rather large range of G^1^, and since small perturbations are present at both 6*s* and 7*s*, it makes little sense to debate the exact value of G^1^ in this approximation. A strict least squares fit gives $G_{1}=\frac{1}{3}G^{1}$ as 172 K and 62 K for 6*s* and 7*s*, respectively. In the preparation of table 11, however, we have assumed that G_1_ varies approximately as (n*)^−3^ in accordance with theory and have taken G_1_(4*p*, 6*s*) = 190 K and G_1_(4*p*, 7*s*) =78 K, both based on the value of G_1_(4*p*, 5*s*) found above. The nondiagonal contributions in kaysers to the (^3^P)6*s*, 7*s* energies for P, ^2^P are as follows:

**Figure:**
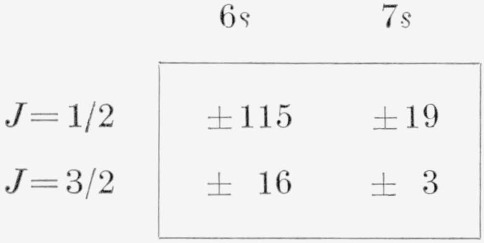


where the upper sign is always taken with the higher level of a given *J*. It is seen that even at 7*s* a positive confirmation of the level assignments can be effected by use of diagonal expressions only.

**Figure 4:**
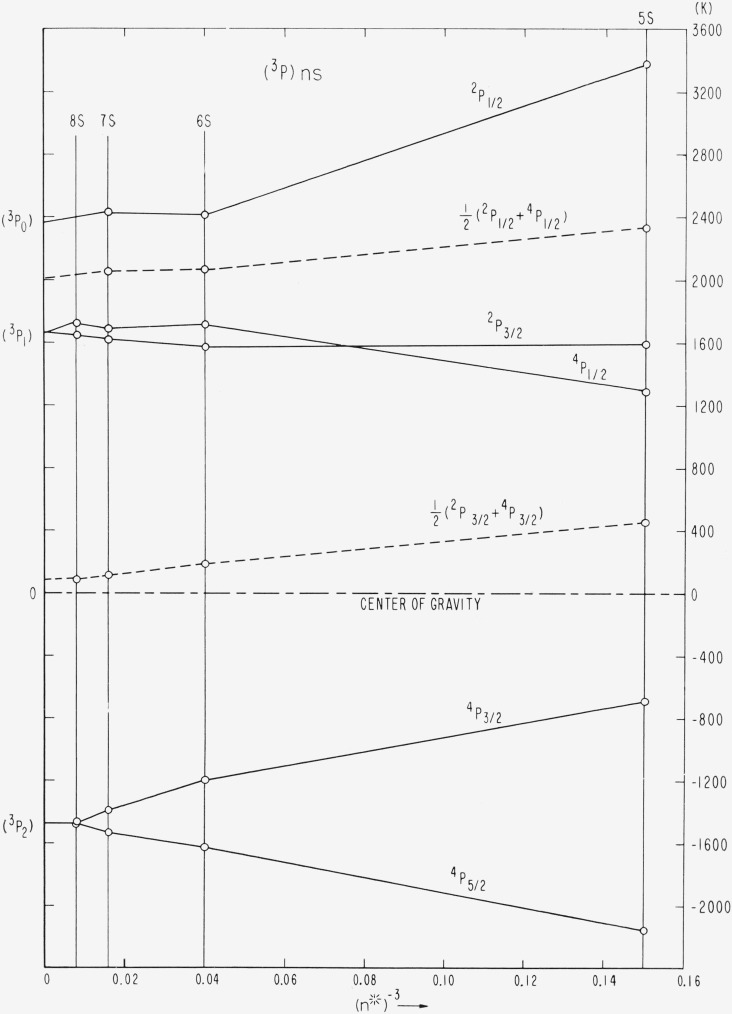
Observed levels of 4p^4^(^3^*P*)ns, referred to center of gravity, and plotted against (n*)^−3^ to show the convergence toward the *Br II* 4p^4 3^*P*_2,1,0_ limits.

Coupling diagrams of the type found in TAS, chapter 11, cannot be constructed for a *p*^4^*s* configuration because the number of parameters necessary to determine the level intervals is greater than two. However, since the splitting of Br II (^3^P) is small compared to the distance from (^3^P) to (^1^D_2_) or (^1^S_0_), the Br I (^3^P)*ns* levels can be described to a satisfactory approximation as a function of the ratio *G*_1_(4*p,ns*)/*ζ_p_.* This is accomplished by omiting in the energy matrices the elements associated with (^1^D_2_) and (^1^S_0_). A coupling diagram showing the structure of *p*^4^(^3^P)*ns* in the transition from *LS* to *J_c_j*- or *J_c_l*-coupling has been constructed by Edlén [22, p. 132]. An equivalent diagram for the observed Br I levels is presented in figure 4 and strikingly agrees with the theoretical behavior of these levels.

Since G_1_ varies approximately as (n*)**^−^**^3^ and *ζ_p_* remains essentially constant along the series, we have plotted in figure 4 the *ns* levels, referred to the center of gravity of each group, against (n*)**^−^**^3^. The mean values ½(^4^P_½_+^2^P½) and ½(^4^P_3/2_+^2^P_3/2_) are also plotted and found to give two approximately straight parallel lines, as expected. The slight irregularities are caused partly by small perturbations of the levels. The observed crossover of the ^4^P_½_ and ^2^P_3/2_ levels is in accordance with theory. It should be noticed that the plot yields an unambiguous coordination of the levels to specific levels of the (^3^P) limit.

**Figure 5:**
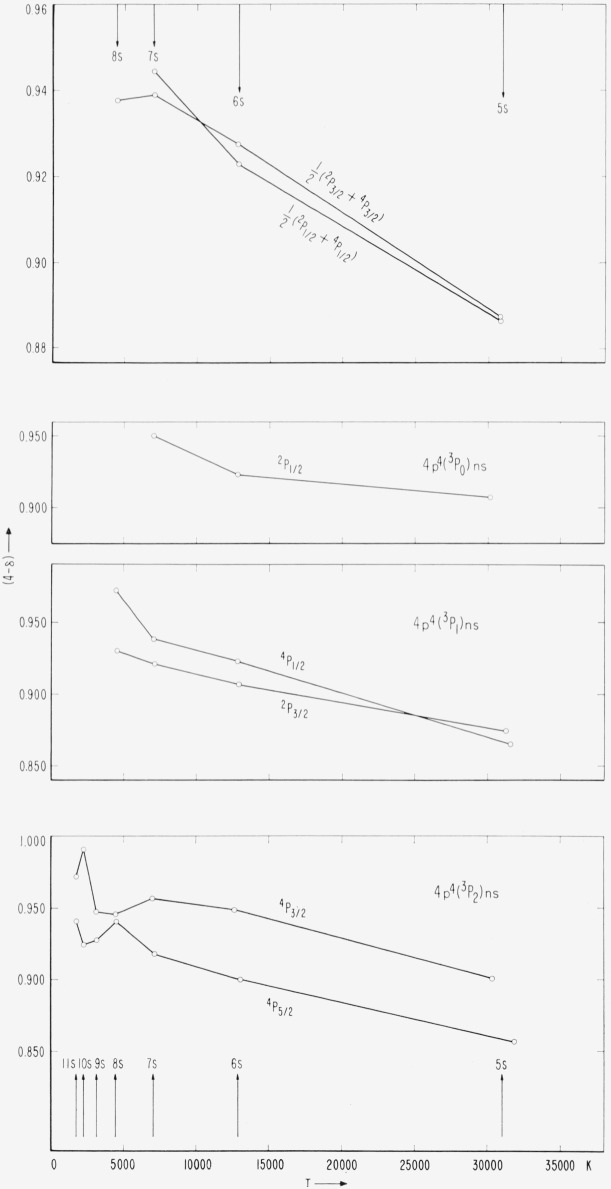
Diagram showing the observed *Br I* ns series.

From a comparative study of the plots in figures 4 and 5 one can obtain a qualitative picture of the perturbations present in the (^3^P)*ns* series, mostly caused by *ns* × *nd* interactions. The displacements of the perturbed levels are all small, being of the order of 20 to 50 K.

The (^3^P_0_) 6*s* ^2^P_½_ level seems to be about 75 K too low, but the cause is not clear, unless this displacement results from the influence of the *J*=½ levels in the (^3^P_2_)5*d* configuration that lie 1000 to 1500 K higher. The (^1^D_2_)4*d*^2^P_½_, ^2^S_½_ levels are probably responsible for the perturbation of (^3^P_1_)8*s* ^4^P_½_, which is about 50 K too high. The higher (^3^P_2_) *ns* series members all suffer slight perturbations owing to *ns* × *nd* interactions. While the plots in figure 5 seem to be highly irregular at the end corresponding to high *n*, it should be remembered that *T* is there relatively small, and n* is therefore quite sensitive to changes in *T*, as is evident from the expression

$$\Delta n*\sim \frac{R^{1/2}}{2T^{3/2}}\Delta T.$$

(b) 4*p*^4^*nd*: Sorting out and interpreting the Br I *nd* energy levels is perhaps the most intricate problem in the analysis of this spectrum. Abundant perturbations and an intermediate type of coupling severely complicate the analysis. We have in the analysis attempted to make the best possible use of the observations. The interpretation of the Br I *nd* levels as presented in table 6 seems to be the most reasonable derivable from general arguments, observed line intensities, application of the Ritz formula, approximate calculations, and the like. A more detailed study of the *nd* configurations based on accurate calculations including interconfigurational interaction might well necessitate the revision of some of the assignments, particularly of those levels having low *J*-values. Reconsideration of the *nd J*=½ levels might even bring the interpretation of (^1^S_0_) 5*s* ^2^S_½_ into question, as pointed out above.

The separation of the *nd* levels of each configuration into groups according to the parent limits is quite apparent. The (^3^P_1,0_)4*d*, *5d*, 6*d* levels all lie below the (^3^P_2_) limit and have been found. The (^3^P_1_)7*d* levels are predicted to fall below the (^3^P_2_) limit but are so close (~ 94900 K) to this limit that they have not been discovered. All (^3^P_2_)*nd* series are complete through 8*d*; each series thus contains five members. The (^3^P_2_)*nd*^4^F_9/2_ series is known through 12*d.* Of the *nd* levels based on the high (^1^D_2_) and (^1^S_0_) parents, only the (^1^D_2_)4*d* group falls below the (^3^P_2_) limit. All levels in this group have been identified at least tentatively.

The grouping of the *nd* levels according to parent suggests that the coupling of these configurations is intermediate between the *J_c_l* and *J_c_j* schemes. The diagonal energy expressions for *p*^4^*d* in these two schemes have been calculated by Möller and Källén, respectively, and can be found in the paper by Minnhagen [19]. A comparison of the *J_c_l* expressions with the observed levels reveals that even the observed ordering of *K*-pairs with respect to energy is not reproduced by the diagonal *J_c_l* expressions on the assumption that the dominant contribution to the energy is due to F^2^(4*p*, *nd*).

It is felt that the reason for this lies, at least in the case of 4*d*, in the magnitude of the G^1^ integral, whose effect on the energies is probably greater than that of F^2^, implying a large departure from *J_c_l* coupling, which requires a small G*^k^* contribution. The large G^1^ contribution probably accounts in part for the high position of the (^3^P_2_)4*d*[2]_5/2_,_3/2_ levels. It may be that the levels (^3^P_2_)4*d*[3]_5/2_ and [2]_5/2_ should have their designations interchanged. The interaction of the 4*d* levels having *J*=5/2, 3/2 with the (^1^D_2_)5*s* ^2^D_5/2,3/2_ levels contributes to the high position of the 4*d* pair just mentioned, but this effect is probably less significant than the size of the G^1^ integral.

The large departures from any pure coupling scheme in the *nd* configurations, as well as sizeable perturbations, render the assignment of designations to the levels a rather difficult problem. Both the *LS* and *J_c_l* symbols assigned these levels in table 6 are based on the intensities of the 4*d–nf*, 5*p–*4*d*, and 5*p–*5*d* combinations. There still remain several inconsistencies; in particular the combining properties of some levels vary irregularly along a series. Only a very detailed consideration of perturbations, together with accurate calculations, can aid in the solution of this problem. Even a cursory examination of the effective quantum numbers of the *nd* levels reveals marked irregularities along series. Apart from small interaction with some *ns* levels, the perturbations are primarily a result of the overlapping or proximity of groups of levels with different *n* and based on different parents. The most striking case of this is the (^3^P_1_)5*d×* (^3^P_2_)6*d* interaction, which causes the (^3^P_1_)5*d* group of levels to be slightly higher than expected. It is very possible that the (^3^P_2_)6*d* ^4^F_7/2_ and (^3^P_1_)5*d* ^2^F_7/2_ levels should have their designations interchanged. A (^3^P_1_)6*d×* (^3^P_2_)9*d* interaction has apparently pushed the (^3^P_1_)6*d* group down. The separation of these latter groups is difficult, however, and the assignments should be regarded as tentative. Their further interaction with the slightly higher (^1^D_2_)4*d* levels is also probable.

The (^1^D_2_)4*d* ^2^G_9/2,7/2_ levels have been established with certainty (see table 6). The other seven levels of the (^1^D_2_)4*d* group have received their designations primarily on the basis of their position being such that they do not fit well into any (^3^P) groups. (Furthermore, we had just seven levels left over after unraveling the (^3^P)*nd* levels.) The order of the (^1^D_2_)4*d* levels bears a striking resemblance to the corresponding group in Kr II, but is in sharp disagreement with the *J_c_l* diagonal expressions (assuming dominant F^2^), which predict the *J* =½ levels to be the lowest.

**Figure 6:**
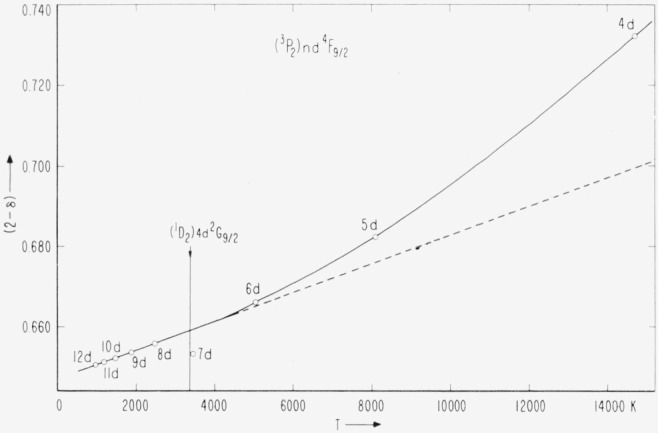
Diagram showing the very regular (^3^*P*_2_)nd ^4^*F*_9/2_ series. The (^1^D_2_)4d ^2^G_9/2_ level was found from its influence on the 7*d* member of this series.

The most important *nd* series showing any degree of regularity is the (*^3^P*_2_)*nd* ^4^F_9/2_ series, for which (2 – *δ*) is plotted against *T* in figure 6. The nonlinearity of this plot is interpreted not as a failure to have the correct ionization potential, but as the effect of a gradual change in coupling along the series. The members from 9*d* through 12*d* are almost exactly linear. A change in the assumed ionization potential would cause the line connecting these levels to curve up or down. A least squares calculation of the Ritz constants *α* and *β* from these levels gives *α* = 1.35276, *β* = − 3.442 × 10^−6^, from which the entire series has been calculated. The results, given in table 12, reveal not only by how much the early series members lie too high because of the different coupling conditions, but also by how much the 7*d* and 8*d* levels have been pushed down and up, respectively, by the (^1^D_2_)4*d* ^2^G_9/2_ level between them.

These slight deviations of the points representing (^3^P_2_)7*d*, 8*d* ^4^F_9/2_ indicate with certainty the presence of the nearby perturbing level, which can be only (^1^D_2_)4*d* ^2^G_9/2_. Until this perturbation was recognized, the only strong Br I lines still remaining unclassified were those at 6096 and 6133 A. That the line at 6096 Å results from the transition $\left({\;}^{3}P_{2}\right)5p{\;}^{4}D_{7/2}^{{}^{\circ}}-\left({\;}^{1}D_{2}\right)4d{\;}^{2}G_{9/2}$ is unquestionably correct, because (i) it fortunately happens to be one of the two unclassified lines whose hfs were reported by Tolansky and Trivedi [7], its observed structure fitting fairly well the splitting of $\left({\;}^{3}P_{2}\right)5p^{4}D_{7/2}^{{}^{\circ}}$ derived from other lines, and (ii) it yields a value 91922 K for (^1^D_2_)4*d* ^2^G_9/2_, which is the proper position to account for the perturbation of the *nd* ^4^F_9/2_ series.

The other line, at 6133 A, is interpreted as the transition $\left({\;}^{3}P_{2}\right)5p{\;}^{4}D_{5/2}^{{}^{\circ}}-{(}^{1}D_{2})4d{\;}^{2}G_{7/2}$. One additional line has been observed in the radiometric region for each of the ^2^G levels. These are combinations with ${(}^{1}D_{2})5p{\;}^{2}F_{7/2,5/2}^{{}^{\circ}}$ and serve as further evidence for the reality of the ^2^G levels.

(c) 4*s*4*p*^6^: The 4*s*4*p*^6^ configuration yields only one level, ^2^S_½_. Bromine is the only halogen for which this level has been found with certainty. A calculation based upon the irregular-doublet law predicts the ^2^S_½_ term to lie near 87000 K. Of the nine even levels with *J* = 1/2 that were found in the range 80000 – 90000 K, only eight can arise from configurations other than 4*s*4*p*^6^. These can all be unambiguously identified on the basis of position and intensity considerations. The remaining level, at 84825.60 K, must be 4*s*4*p*^6 2^S_½_. This identification explains the high intensity of the ultraviolet lines at 1232 Å, resulting from the transition 4*s*^2^4*p*^5^ ${\;}^{2}P_{½}^{{}^{\circ}}-84825$, and at 1179 Å, 4*s*^2^4*p*^5^ ${\;}^{2}P_{3/2}^{{}^{\circ}}-84825$: the ground ^2^P° term is the only known term to which the 4*s*4*p*^6 2^S_½_ can make transitions in a one-electron jump.

In addition to the ultraviolet lines and a faint, wide line at 12369 A, the only other lines from ^2^S_½_ that have been observed are two moderately strong doublets at 10184 Å and 11094 Å. The doublet character of each of these lines is assumed to result from the wide hfs splitting of ^2^S_½_, which is expected, since the unpaired 4*s* electron in the 4*s*4*p*^6^ configuration should couple strongly with the nucleus. The classifications of these lines are as follows:

10184Å5p4P3/2°−sp62S1211094Å5p4P1/2°−sp62S12

The components of 11094 Å appear single with our resolution but there is slight shading in the two components of 10184 Å. This results from the hfs splitting of 5*p* ${\;}^{4}P_{3/2}^{{}^{\circ}}$, which according to Tolansky and Trivedi has a structure (0.127, 0.086, 0.043) in K. To get an estimate of the splitting of the ^2^S_½_ level, we note that the separations of the components of 10184 Å and 11094 Å are 0.45 and 0.59 K, respectively. The large difference between these two values might at first seem disturbing. But a simple sketch of the appropriate transitions, taking into account the known hyperfine structure of ${\;}^{4}P_{3/2}^{{}^{\circ}}$ (four *F*-levels) and adopting any reasonable value for the unknown structure of ${\;}^{4}P_{½}^{{}^{\circ}}$ (two *F*-levels), reveals that one would expect the components of 10184 Å to be about 0.1 K closer together than the components of 11094 A. Furthermore, it is apparent from such a sketch that the measured separation of the centers of gravity of the 11094 Å components very nearly represents the true hfs splitting of the ^2^S_½_. We therefore adopt 0.59 K as the splitting of the ^2^S_½_ level.

A calculation of the theoretical structure of this level is revealing. Following Slater [23], we can write the expectation value of the nuclear interaction contribution to the total Hamiltonian as

$$\langle H^{\prime }\rangle =\frac{\alpha^{2}}{3}g_{N}R^{2}(0)K\;\text{rydbergs}.$$

(This formula holds only for configurations containing a single unpaired *s*-electron.) 7 Here *α* is the fine structure constant (1/137), *g_N_* is the weighted average bromine nuclear *g*-factor (= 7.93×10^−4^), *R*(0) is the value of the normalized *s*-eigenfunction at the origin, and

K=F(F+1)−I(I+1)−J(J+1).

Since *J*=1/2 and the nuclear spin *I* = 3/2, the energy separation of the two *F*-levels will be

$$\Delta E=\frac{4\alpha^{2}}{3}g_{N}R^{2}(0)\;\;\text{rydbergs}.$$

We have calculated *R*(0) from recently published [24] Hartree-Fock radial wave functions for the bromine 4*s*^2^4*p*^5^ configuration. The 4*s*-orbital of this configuration will not be very different from that of the 4*s*4*p*^6^ configuration. We find *R*(0) = 15.6. Inserting numerical values in the above equation, we finally obtain

$$\begin{array}{ll} E=1.37\times 10^{-5} & \text{rydbergs}\\ \;=1.50 & \text{kaysers}. \end{array}$$

(A relativity correction would increase this value by about 14%.) The above result is to be compared with the observed value 0.59 K. Some of the discrepancy between these values must arise from configuration interaction. Such an interaction is undoubtedly present, because the two lines under discussion are examples of so-called two-electron jumps. The occurrence of these lines at all indicates that the *sp*^6^ configuration is mixed with a configuration that would make the transition in a one-electron jump. The interaction involved here is probably with one of the *J*=1/2 levels of the 4*d* configuration, possibly 4*d* ^4^P_1/2_. One would expect the level with which the *sp*^6 2^S_1/2_ is mixed to share some of the latter’s hyperfine structure. Each of the 4*d* levels with *J* = 1/2 was checked for hfs, but none could definitely be established from our observations.

## 6. Levels of Odd Parity

(a) 4*p^4^np:* The analysis of the system of odd levels of Br I was enormously simplified by the occurrence of almost pure *J_c_l*-coupling in the *nf* configurations. Upon the identification of a few of the *nf* levels, the rest could easily be calculated and identified. We could then safely regard all odd levels left over as belonging to excited *up* configurations.

With the exception of the (^3^P_1_)8*p* group, which is predicted to center around 94675 K, all *np* levels for *n*=5, 6, 7, 8, 9 that fall below the (^3^P_2_) limit have been found. Two levels belonging to (^3^P_2_)10*p* have been identified also. Of the levels based on the (^1^D_2_, ^1^*S*_0_) parents, only the (^1^*D*_2_)5*p* levels are below the (^3^P_2_) limit. As in the *ns* and *nd* configurations, the *np* levels fall into groups that reflect the structure of the Br II *p***^4^** parents. The coupling of the higher *np* configurations is between the *J_c_l* and *J_c_j* schemes, but apparently closer to *J_c_l.* Happily, configuration interaction of the *np* set of levels with the *nf* set is extremely small. The very different coupling exhibited by these two sets of odd levels may partly account for this minimal interaction.

The matrices of spin-orbit interaction for a *p*^4^*p* configuration have been calculated in an *LS* basis and are presented in the following paper in this issue of the Journal of Research. A study of the Br I *np* configurations by means of these matrices has not yet been completed. The results of the calculations will appear later, together with the results of Zeeman observations in progress.

It may be mentioned that the electrostatic [18] and spin-orbit [19] elements of the *p*^4^*l* matrices for pair-coupling have just been published, as well as the *p^4^l* matrices for *J_c_j*-coupling [19], calculated by Källén. The assignment of *K*-values to the Br I *np* levels is based on intensities and approximate calculations with the *J_c_l* diagonal energy expressions given in reference [19]. The *LS* symbols are based on the intensities of *5p*—5*s*, 6*s*, 4*d* transitions.

The Br I 5*p* configuration is rather isolated and except for a few small interactions is regarded as a pure configuration. It should therefore be possible to represent quite accurately in parametric form the distribution of the 5*p* levels. It was for this reason, in fact, that the *p*^4^*p* matrices in intermediate coupling were calculated. Since the (^1^S_0_) 5*p* ^2^P° term is above the (^3^P_2_) limit and has not been observed, it is not possible to use the diagonal sum rule in the calculation of the parameters. The parameters may, however, be estimated in a variety of ways based on diagonal energy expressions in the several coupling schemes for levels of high *J*-value, on approximately calculated positions for (^1^S_0_) 5*p* ^2^P°, and on level differences. The approximate parameters found in this way can be inserted in the energy matrices and improved by iteration.

The most interesting interaction occurring with the 5*p* configuration is perhaps the $\left({\;}^{1}D_{2}\right)5p{\;}^{2}D_{5/2,3/2}^{{}^{\circ}}\times \left({\;}^{3}P_{2}\right)4f{\left[2\right]}_{5/2,3/2}^{{}^{\circ}}$ interaction. The (^3^P_1_)6*p*^2^D° term lies somewhat higher but may also be involved. Accurate calculations given below show that the two 4*f* levels have been pushed down by 13 K and 21 K for *J*=5/2 and *J*=3/2, respectively. This partly accounts for the surprisingly strong intensity of (^1^D_2_)5*s* ^2^D −(^3^P_2_)4*f*[2]° transitions, as pointed out above.

The $\left({\;}^{3}P_{2}\right)5p{\;}^{4}D_{3/2}^{{}^{\circ}}={\left[1\right]}_{3/2}^{{}^{\circ}}$ level seems to lie abnormally high, judging from the diagonal *J_c_l* energy expressions. This same behavior was noticed in the case of I I by Minnhagen [19], who calculated the 11 6*p* levels by use of the F^2^(5*p*, 6*p*) parameter only. It was suggested that configuration interaction might be responsible for the high position of the level in question. As evidence for such an interaction, Minnhagen cited the fact that the intensity of the I I line classified as $\left({\;}^{3}P_{2}\right)6s{\left[2\right]}_{5/2}-\left({\;}^{3}P_{2}\right)6p{\left[1\right]}_{3/2}^{{}^{\circ}}\left(=8044\text{ Å}\right)$ is much stronger than that of the line $\left({\;}^{3}P_{2}\right)6s{\left[2\right]}_{5/2}-\left({\;}^{3}P_{2}\right)6p{\left[3\right]}_{7/2}^{{}^{\circ}}(=9058\;Å)$ whereas the latter line should be stronger for all unperturbed couplings. The only J=3/2 level below I I (^3^P_2_)6*p* is the ground level 5*p*^5^ ${\;}^{2}P_{3/2}^{{}^{\circ}}$, which renders perturbation difficult to accept. It seems reasonable that the intensity ratio of I I lines just mentioned is, rather, a result of the fact that 8044 Å falls at the wavelength of peak sensitivity of EK–“N” photographic plates, while 9058 Å was undoubtedly photographed on “Q” or *“*Z*”* plates, which are less sensitive than “N” plates by about a factor of 10. In Br I, the corresponding fines both occur in the “N” region, and the relative intensities are in the expected order. It is felt that the high position of the level in question, both in I I and Br I, is to be explained by the magnitude of the G^0^ (*np*, *n'p*) integral, which contributes sizeably to the Br I 5*p* and 1 I 6*p* energies and should not be neglected. The *J_c_l p*^4^*p* diagonal energy expressions for (^3^P_2_)*np* [2]°, [1]° are, except for an additive constant:

$$\begin{array}{l} {\left[2\right]}_{5/2}^{{}^{\circ}}-\frac{7}{50}F^{2}\;+\frac{18}{600}G^{2}+\frac{1}{6}\zeta_{n^{\prime }p}\\ {\left[2\right]}_{3/2}^{{}^{\circ}}-\frac{7}{50}F^{2}+\frac{45}{24}G^{0}+\frac{153}{600}G^{2}-\frac{1}{4}\zeta_{n^{\prime }p}\\ {\left[1\right]}_{3/2}^{{}^{\circ}}+\frac{7}{50}F^{2}+\frac{5}{24}G^{0}+\frac{17}{600}G^{2}-\frac{1}{4}\zeta_{n^{\prime }p}\\ {\left[1\right]}_{1/2}^{{}^{\circ}}+\frac{7}{50}F^{2}+\frac{20}{24}G^{0}+\frac{50}{600}G^{2}+\frac{1}{2}\zeta_{n^{\prime }p} \end{array}$$

where all integrals refer to the (*np, n'p*) interaction. Approximate Br I 5*p* parameters, derived as described above, indicate that the effect of G^0^ on ${\left[2\right]}_{3/2}^{{}^{\circ}}$ brings its diagonal energy very close to that of ${\left[1\right]}_{3/2}^{{}^{\circ}}$. Since the two levels are connected by G^0^, G^2^, and ζ*_n_*_′_*_p_*, they repel each other in such a way, neglecting the action of the other levels in the configuration, as to make ${\left[1\right]}_{3/2}^{{}^{\circ}}$ appear high and the *K*=2 levels appear as a closer pair. The ${\left[1\right]}_{1/2}^{{}^{\circ}}$ level is pushed down by all 5*p J*=1/2 levels above it. This same type of effect is probably operating also in the case of the high Br I (^3^P_2_)4*d*[2] pair, as mentioned previously.

**Figure 7:**
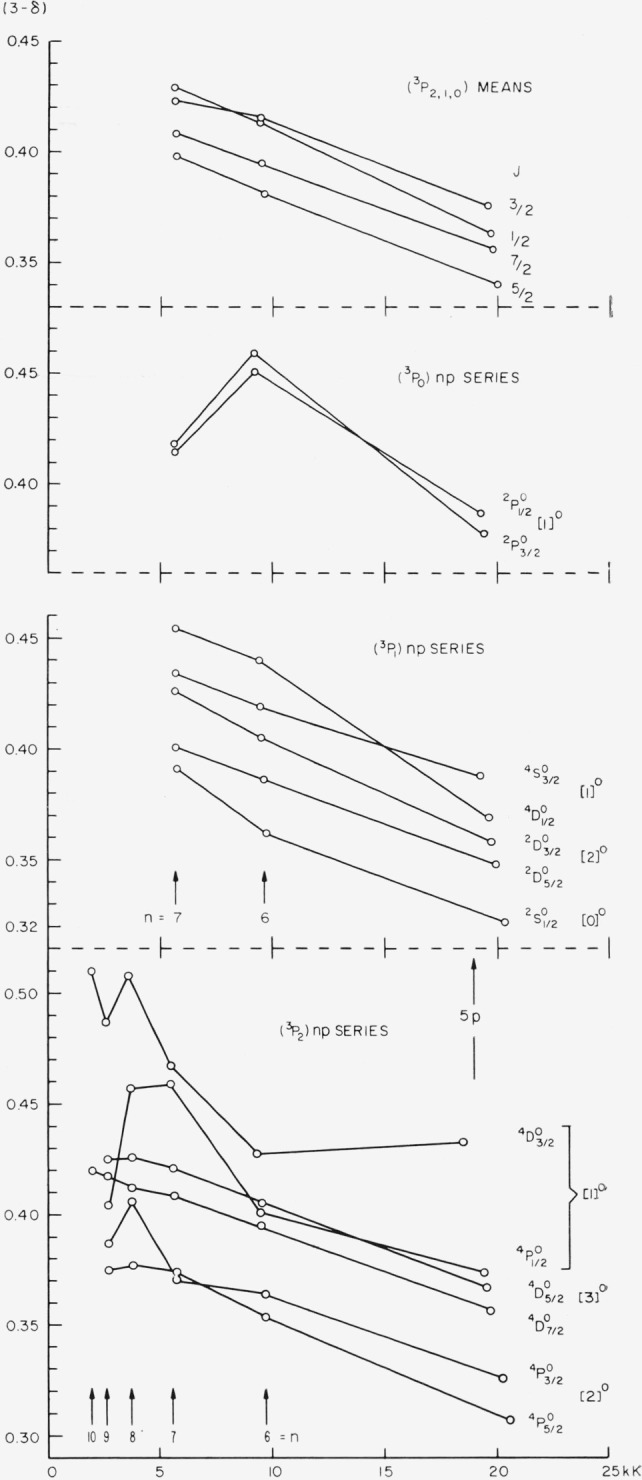
The (^3^*P*)np series: (3 — *δ*) plotted against *T*.

A qualitative picture of the run of Br I *np* levels and perturbations can be obtained from figure 7, in which the quantum defect is plotted against absolute level value. The points representing the (^3^P_2_)*np* ^4^D_7/2_ series are very nearly linear for *n*=6, 7, 9, 10. The slight depression at *n*= 8 is due to the interaction with $\left({\;}^{3}P_{1}\right)4f{\left[3\right]}_{7/2}^{{}^{\circ}}$. An application of the Ritz formula with *α* = 2.57410, *β*= +3.190×10^−6^ derived by least squares from (^3^P_2_) 7, 9, 10*p* $4D_{7/2}^{{}^{\circ}}$, reproduces these levels almost exactly and shows that the level at 8*p* is depressed by about 2.6 K, its “unperturbed” position being 91540.9 K. The same Ritz formula predicts (^3^P_2_)5*p* $4D_{7/2}^{{}^{\circ}}$ to fall 114 K higher than observed, the departure being a result of the regular coupling change along the series.

The great deviation from linearity exhibited in figure 7 by the early members of the (^3^P_2_)*np* ${\;}^{4}D_{3/2}^{{}^{\circ}}$, ${\;}^{4}P_{1/2}^{{}^{\circ}}$ and (^3^P_0_)*np* ${\;}^{2}P_{3/2,1/2}^{{}^{\circ}}$ series is probably not entirely due to perturbations, since the plots of mean term energies for each *J*-value are much more regular. However, the possibility of a configuration interaction affecting these levels in such a way as approximately to cancel out in the means should not be overlooked. It seems fairly certain that there is a (^3^P_0_) 6*p* × (^3^P_2_) 7*p* interaction, and the (^3^P_2_)6*p* ${\;}^{4}D_{3/2}^{{}^{\circ}}$, ${\;}^{4}P_{1/2}^{{}^{\circ}}$ levels may be influenced by (^1^D_2_)5*p* ${\;}^{2}P_{3/2,1/2}^{{}^{\circ}}$. The (^3^P_2_)7*p*, 8*p*, ${\;}^{4}P_{3/2}^{{}^{\circ}}$ levels seem to have been pushed down and up, respectively, through interaction with (^3^P_0_)6*p* ${\;}^{2}P_{3/2}^{{}^{\circ}}$, which lies between them.

The most obvious wholesale mutual interactions in the *np* configurations affect the close (^3^P_1_)7*p* and (^3^P_2_)9*p* groups, the levels of the first group being higher than expected and those of the second group (except 9*p* ${\;}^{4}D_{7/2}^{{}^{\circ}}$) lower.

(b) 4*p*^4^*nf:* All the 67 levels ascribed to *nf* configurations in table 5 are new. The pronounced *J_c_l* coupling prevailing in these configurations permits the levels to be calculated very exactly by the theoretical expressions of electrostatic energy given by Moller [18]. The *J_c_l* designations of the levels as presented in table 5 are therefore, with one or two exceptions discussed below, definite. The pair structure of these levels is very marked, as can be seen by examining the pair splittings in table 5. All *nf* levels based on limits other than (^3^P_2_) and predicted to lie below the (^3^P_2_) limit have been found. These are the 16 levels of the (^3^P_1,0_)4*f*, 5*f* groups. All (^1^*D*>_2_, ^1^*S*_0_)*nf* levels lie above the (^3^P_2_) limit.

Most of the observed transitions involving the Br I *nf* levels occur in the (^3^P_2_)4*d*−(^3^P)*nf* arrays. As mentioned above, 5*s–nf* transitions are also observed, primarily as a result of a 5*s*×4*d* interaction, but nearly all other lines involving *nf* levels would be difficult to observe since they fall in a very unfavorable region of the spectrum, the far infrared and radiometric regions. The (^3^P_2_)4*d*—(^3^*P*_2_)*nf* arrays are similar for each value of *n* as regards the intensity relationships of the various lines in the array, but the intensity of any particular transition decreases as a rule with increasing *n.* In general, for transitions between two pair-coupled configurations the lines will be stronger when *l*, *K*, and *J* change by unity in the same direction, or, with a given Δ*l*(*= ±* 1), when Δ*J*=Δ*K*(= ± 1, 0). As usual, also, the stronger lines involve the higher *J*-values. The representative (^3^P_2_)4*d*−(^3^P_2_)5*f* array is shown in table 13.

In this table, the adopted *K*-values of the (^3^P_2_)4*d* levels are found together with the corresponding *LS* designations. The only rigorous selection rule here seems to be that on *J.* It will be noticed, however, that three transitions from 5*f* in this table that would be allowed by the selection rule on *J*, but not by the selection rule on *K* in pure *J_c_l* coupling have not been observed. The same is true in the case of these three transitions for other *nf*, except that $\left({\;}^{3}P_{2}\right)4d{\left[3\right]}_{7/2}-\left({\;}^{3}P_{2}\right)4f{\left[5\right]}_{9/2}^{{}^{\circ}}$ occurs weakly, owing to the “mixing” of the two (^3^P_2_)4*d* levels having *J*=7/2. On the other hand, for the 5*f* case in table 13 as well as for other *nf*, there appear strongly two lines that do “violate” the Δ*K*=0, ±1 rule. These are the $\left({\;}^{3}P_{2}\right)4d^{4}D_{3/2}-\left({\;}^{3}P_{2}\right)nf{\left[3\right]}_{5/2}^{{}^{\circ}}$ and $\left({\;}^{3}P_{2}\right)4d^{4}F_{5/2}-\left({\;}^{3}P_{2}\right)nf{\left[4\right]}_{7/2}^{{}^{\circ}}$ transitions. For no value of *n* have the two $\left({\;}^{3}P_{2}\right)4d^{4}F_{5/2,3/2}-\left({\;}^{3}P_{2}\right)nf{\left[2\right]}_{3/2}^{{}^{\circ}}$ lines been observed. The perturbations that affect the (^3^P_2_)4*d* levels having *J*=5/2, 3/2 and the difficulty at present of appraising the quantum significance of the *K*-values assigned these levels, as mentioned in another section, render premature any detailed correlation of intensity characteristics in the arrays under discussion.

**Figure 8:**
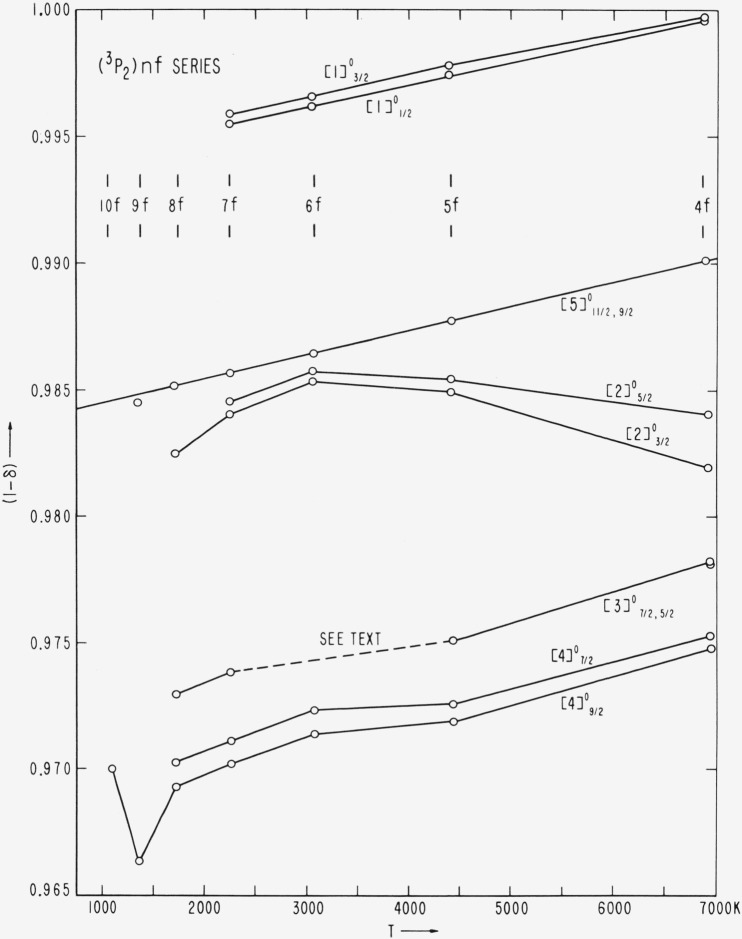
The (^3^*P*_2_)nf series plotted on a very expanded (1−δ) scale to exhibit small perturbations.

The several perturbations that afflict the (^3^P_2_)*nf* series are vividly revealed by a plot of quantum defects against relative term values for these series (fig. 8). In examining this figure, one should bear in mind that the ordinate scale is greatly expanded, and the irregularities in the plots are caused by level perturbations of only a few kaysers and less. An idea of the vertical scale in kaysers can be gained for each value of *n* by noting the separation of the various pairs. For example, at *n*=7, the vertical separation between $7f{\left[4\right]}_{9/2}^{{}^{\circ}}$ and $7f{\left[4\right]}_{7/2}^{{}^{\circ}}$ corresponds to only 0.57 K, an amount that is probably greater than that by which either level is perturbed. The perturbations in the Br I *nf* configurations are most conveniently discussed in terms of the *J_c_l* nondiagonal matrix components. We shall therefore consider first the energy matrices and the theoretical calculation of the *nf* energy level distribution.

The matrices of electrostatic energy are diagonal in *J.* The diagonal elements of the matrices for *p*^4^*f* have the form $E=E\;\left(\text{parent level}\right)-2F^{0}-2{\bar{f}}_{2}^{\prime }F^{2}+2{\bar{g}}_{2}^{\prime }G^{2}+2{\bar{g}}_{4}^{\prime }G^{4},$ where ${\bar{f}}_{2}^{\prime }$, ${\bar{g}}_{2}^{\prime }$, ${\bar{g}}_{4}^{\prime }$ are numerical coefficients whose values can be found in Moller’s paper, and F^2^, G^2^, G^4^ are the familiar integrals defined in TAS. Levels of a given *K*-pair have a common ${\bar{f}}_{2}^{\prime }$ coefficient; the pair splitting with respect to ***J*** arises from different ${\bar{g}}_{2}^{\prime }$ and ${\bar{g}}_{4}^{\prime }$ coefficients. Small pair splittings, therefore, imply small G^2^ and G^4^ integrals. In the Br I *nf* configurations, the contribution to the level energies by the F^2^ integral is much greater than that by the G^2^ and G^4^ integrals. Nondiagonal F^2^ is found only between levels with equal *K*-value and based on the different parents, ^4^S_0_− ^1^D_2_, ^3^P_0_−^3^P_2_, and ^3^P_1_−^3^P_2_. Also appearing off the diagonal are G^2^ and G^4^, connecting levels whose *K*-values are equal or are different by ±1. In order that *K* be a good quantum number, it is thus essential that G^2^ and G^4^ be small compared with F^2^.

In the actual calculation of the *nf* level distribution, it is convenient and suitable to make certain approximations. All (^1^S_0,_^1^D_2_)*nf* levels fall above the principal ionization limit and none was observed. But since these levels are distant and are connected to the levels based on (^3^P) only through nondiagonal G^2^ and G^4^ (and ζ_4_*_p_*), the effect of omitting these levels from the energy matrices will cause negligible error in the calculated (^3^P)*nf* energies. Furthermore, greater accuracy in the calculated distribution of these (^3^P)*nf* levels (18 levels for each *n*) can be expected if we employ the observed positions of the Br II (^3^P) levels rather than adopting the parametric form for these parents. We thus replace the term *E* (parent level) — 2F^0^ in the above diagonal expression by the quantities: *B*, *B*+3136.4, and *B+* 3837.5, for levels based on (^3^P_2_), (^3^P_1_), and (^3^P_0_), respectively, where *B* is a constant for any given *n.* This procedure automatically accounts for nondiagonal ζ_4_*_p_*, which may then be disregarded.

We thus have 18 levels for each *n* to be represented in parametric form by four parameters, B, F^2^, G^2^, and G^4^. The determination of meaningful parameters cannot be accomplished by a simple application of the diagonal sum rule, because every observed Br I *nf* level, with the exception of members of the (^3^*F*_2_)*nf*[*5*]*°* series, suffers interaction with other configurations. The extent of this interaction can be reasonably well estimated only in the case of the $\left({\;}^{3}P_{2}\right)nf{\left[4\right]}_{9/2}^{{}^{\circ}}$ levels, because almost the entire interconfigurational contribution to these must come from $\left({\;}^{3}P_{1}\right)nf{\left[4\right]}_{9/2}^{{}^{\circ}}$ levels, there being no possibility of a *J*= 9/2 in the *np* configurations. After G^4^ has been estimated as described below, F^2^ can be found from the (3P2)nf[5]11/2°−[4]9/2°=16F2−136G4 diagonal interval, provided the nondiagonal intraconfigurational contribution to ${\left[4\right]}_{9/2}^{{}^{\circ}}$, as well as the interconfigurational contribution, have first been removed from the energies as observed. The first of these contributions can be determined by performing a preliminary diagonalization of the *J* = 9/2 matrix with a good estimate of F^2^. The second contribution can be obtained if a reasonable “unperturbed” $\left({\;}^{3}P_{2}\right)nf{\left[4\right]}_{9/2}^{{}^{\circ}}$ series can be deduced. If an interaction parameter (see ref. [22], p. 141) γ = 23 K is assumed between the $\left({\;}^{3}P_{2}\right)nf{\left[4\right]}_{9/2}^{{}^{\circ}}$ and $\left({\;}^{3}P_{1}\right)nf{\left[4\right]}_{9/2}^{{}^{\circ}}$ series, the former series becomes satisfactorily Kitzian, and after an additional “smoothing” of the series, unperturbed level energies can be estimated to sufficient accuracy, and then F^2^ can be derived.

Since nondiagonal contribution to the (^3^P_2_)*nf*[*5*]*°* pair is negligible, the G^4^ parameter can be obtained directly from the observed separation of this pair, whose diagonal energy expressions, including the spin-orbit interaction energy of the *f*-electron, are

$$\begin{array}{l} {\left[5\right]}_{11/2}^{{}^{\circ}}:B+\frac{1}{15}F^{2}\;+\frac{3}{2}\zeta_{f}\\ {\left[5\right]}_{9/2}^{{}^{\circ}}:B+\frac{1}{15}F^{2}+\frac{11}{54}G^{4}-\frac{9}{5}\zeta_{f}. \end{array}$$

The fact that for *n*≧5, the ${\left[5\right]}_{9/2}^{{}^{\circ}}$ falls higher than ${\left[5\right]}_{11/2}^{{}^{\circ}}$ confirms the dominance of $\frac{11}{54}G^{4}$ over $\frac{33}{10}\zeta_{f}$ and partially justifies the omission of *ζ_f_* in our calculations. The G^4^ parameter for 4*f* has been extrapolated from those for higher *n*, since the observed coincidence of the 4*f* pair, suggesting an equality of $\frac{11}{54}G^{4}$ and $\frac{33}{10}\zeta_{f}$ does not permit a direct calculation of G^4^.

If the $\left({\;}^{3}P_{2}\right)nf\left[1\right]{}^{\circ}$ levels were unperturbed, G^2^ could be calculated fairly accurately from the pair splitting, $\frac{9}{50}G^{2}$. Even though this series as plotted in figure 8 appears Ritzian, there is some evidence that the series members have all been perturbed upwards in such a way that linearity of the plot was preserved. Nevertheless, G^2^ was crudely taken as given by the pair splittings for *n*≧5 and extrapolated for *n* =4, since any other procedure meets with equivalent uncertainty. The remaining parameter, B, was adjusted so as to predict the $\left({\;}^{3}P_{2}\right)nf{\left[5\right]}_{11/2}^{{}^{\circ}}$ levels exactly.

The levels as calculated by use of the parameters found above are compared with the observed positions in table 14 for 4*f*, 5*f* and in table 15 for 6*f*, 7*f*. The B and F^2^ parameters are given in table 16, along with the almost constant products F^2^×*n*^3^. The agreement between observed and calculated energies is seen to be very good, and it is probably significant that the departures in almost every case agree with the direction of perturbation one might expect on the basis of the plots in figure 8. The calculations predict the observed ordering of *K*-pairs with respect to energy for all except the perturbed (^3^P_2_)4*f*[3]° and (^3^P_1_)4*f*[3]° pairs.

The perturbations of the Br I *nf* levels arise from *nf×np* as well as *nf×n'f* interactions. Considering first the latter type, we remember that since the coefficients of the Slater integrals in the *nf* energy matrices are based entirely upon the angular parts of the eigenfunctions, these same coefficients are valid in the discussion of *nf×n′f* interaction. Since this interaction is expected to be greater if a nondiagonal ${\bar{f}}_{2}^{\prime }$ is involved, we should expect that the strongest interactions would be of the types (^3^P_1_)*nf*[K]°× (^3^P_2_)*n*′*f*[K′]°, where *K=K′*=2, 3, or 4, and (^3^P_0_)*nf*[3]°× (^3^P_2_)*n*′*f*[3]°. Indeed, the plots in figure 8 show quite clearly that (^3^P^1^)4*f* falls just between (^3^P_2_)5*f* and 6*f* and pushes (^3^P_2_)4*f*,5*f* levels down and. (^3^P_2_)6*f*,7*f* … levels up, for *K*= 2, 3, 4. Also, the (^3^P_0_)4*f*[3]°×(^3^P_2_)6*f*[3]° interaction is, comparatively, very strong, owing to their almost identical unperturbed positions, as indicated by the calculations. It is possible that the designations of these levels should be interchanged. The points corresponding to (^3^P_2_)6*f*[3]° fall outside the range of figure 8 and have been omitted. Another striking feature of this figure is that it suggests, correctly, that $\left({\;}^{3}P_{1}\right)5f{\left[4\right]}_{9/2}^{{}^{\circ}}$ falls between $\left({\;}^{3}P_{2}\right)9f,10f{\left[4\right]}_{9/2}^{{}^{\circ}}$.

Superposed on the above interactions are those of the type *nf×np*, which also seem to be stronger if the interacting levels have the same *K*-value.

The (^1^D_2_)5*p* group appears to interact with *nf* levels having the appropriate *K*- and *J*-values, the largest such interaction being $\left({\;}^{1}D_{2}\right)5p{\;}^{2}D_{5/2,}{\;}_{3/2}\times \left({\;}^{3}P_{2}\right)4f{\left[2\right]}_{5/2,3/2}^{{}^{\circ}}$, owing to the proximity of the levels. The several interactions affecting (^3^P_2_)*nf*[3]° pairs make the levels for *n****≧***5 coincide within the experimental error. Since it is not possible to decide with certainty from which level of the pair a particular line arises, when that line is classifiable by either of the levels, the two levels are represented by a single energy in table 5.

The calculations suggest that the observed (^3^P_2_) *nf*[1]° pair is uniformly high. Since a *K*= 1 is not possible from the (^3^P_1_) and (^3^P_0_) parents, we should expect the observed [1]° series to be almost Ritzian in the absence of interaction with *np* levels. Now the series *is* almost Ritzian, so that if the series is perturbed, it must be of a complicated sort, involving several *np* levels, since a single interaction with $\left({\;}^{1}D_{2}\right)5p{\;}^{2}P_{3/2}^{{}^{\circ}}{,}_{1/2}$ would not account for the preservation of the rather strict linearity found in figure 8.

The $\left({\;}^{3}P_{2}\right)8p{\;}^{4}D_{7/2}^{{}^{\circ}}\times \left({\;}^{3}P_{1}\right)4f{\left[3\right]}_{7/2}^{{}^{\circ}}$ interaction, complicated by additional influences on these levels, should also be mentioned, since it is possible that the two designations should be interchanged. It is not entirely clear what causes the (^3^P_1_)4*f*,5*f*[3]° levels to be observed so “much” higher than their calculated positions.

## 7. Conclusion

Since the Br I line list has now been essentially exhausted—only 26 of 1253 lines remain unclassified—it is clear that any significant additions to the analysis of Br I will demand a thorough, new observation of the spectrum, made with a much stronger source for reasonable exposure times in the red, and one that produces Br_2_ emission of much less intensity than the source used in this investigation. Even without making accurate calculations, one can predict very closely, barring strong interactions, the positions of the remaining unknown *ns, np, nd*, and *nf* levels that fall below the principal ionization limit. This can be done almost by inspection of tables 5 and 6. One can, then, make educated guesses as to the wavelength region where the stronger lines involving the unknown levels will lie. Of course many of the new lines that would be included in any augmented line list would be accounted for by transitions predicted in the present square array, and might help us in a possible revision of the more insecure level designations mentioned above. A study of the Zeeman effect in Br I is possibly the only and certainly the best method of resolving a few difficulties of interpretation, such as that of the *J*= 1/2 levels lying in the vicinity of (^1^S_0_)5*s*^2^S_1/2_ as discussed above.

Perhaps the most demanding study that could be initiated on Br I is an extensive analysis of the *nd* configurations and their coupling and perturbations. If some of the levels still missing from the (^3^P_2_)9*d*-1 1*d* configurations could be found, a more definite decision could possibly be reached as to whether some levels presently assigned to (^3^P_1_)6*d* might not more properly belong to (^3^P_2_)9*d*.

Of the 26 lines in our list that have defied classification, one has intensity 50, one intensity 15 *w*, and the rest have intensities 10 and less. It is probable that as many as one-half of these lines actually do not arise from neutral atomic bromine. For example, since the six unclassified lines of wavelengths less than 4100 A would have to result from transitions to the even (^3^P)5*s* levels in order for the upper levels to lie below the (^3^P_2_) limit, and since no new odd levels predictable on this basis fall in acceptable positions, it is doubtful that these six lines belong to Br I.

There seems to be little hope of finding any Br I levels above the (^3^P_2_) limit unless autoionization from these levels is somehow avoided. Although we have predicted and looked in our list for many lines that we thought most likely to appear from such levels, none could be established definitely.

The unknown *ng* levels falling below the (^3^P_2_) limit are predicted to lie in the range 90890 to 95285. Their stronger combinations will generally be with *nf* levels. Since these latter levels are in the range 88300 to 95285 K combinations with *ng* will produce lines that fall in the infrared beyond the region accessible by photography.

## Figures and Tables

**Table 1:** Wavenumbers and intensities of selected multiplets observed in the radiometric region

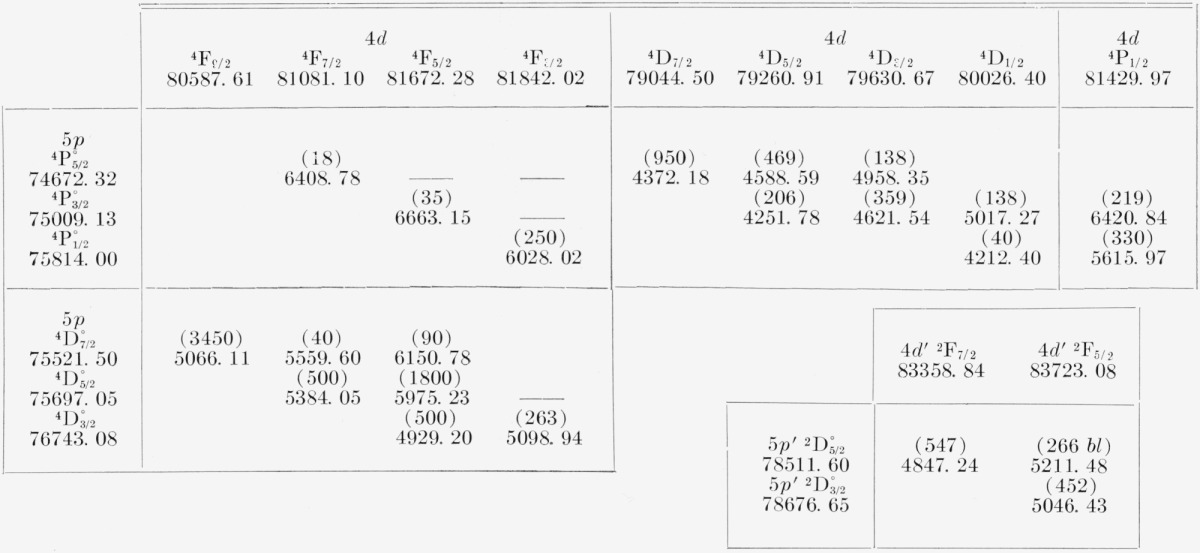

**Table 2:** Some observed multiplets involving the 6*s* ^4^*P* levels

	^4^P_5/2_ 82236.17	6 *s* ^4^P_3/2_ 82661.57	^4^P_1/2_ 85576.93

5*p* ${\;}^{4}P_{5/2}^{{}^{\circ}}$	(1700)	(20)	
74672.32	7563.85	7989.25	
${\;}^{4}P_{3/2}^{{}^{\circ}}$	(750)	(110)	(500*d*)^a^
75009.13	7227.04	7652.44	10567.80
${\;}^{4}P_{1/2}^{{}^{\circ}}$		(338)	
75814.00		6847.57	——

5*p* ${\;}^{4}D_{7/2}^{{}^{\circ}}$	(1250)		
75521.50	6714.67		
${\;}^{4}D_{5/2}^{{}^{\circ}}$		(1800)	
75697.05	——	6964.52	
${\;}^{4}D_{3/2}^{{}^{\circ}}$			(10 *w*)^a^
76743.08	——	——	8833.85
${\;}^{4}D_{1/2}^{{}^{\circ}}$			(55)
78865.72			6711.21

5*p* ${\;}^{4}S_{3/2}^{{}^{\circ}}$			(24)
79178.33			6398.60

a These two lines were observed photographically.

**Table 3:** Predicted, terms of *Br I*

Configuration	Predicted terms				
	
4*s*^2^ 4*p*^5^	^2^P°				
4*s* 4*p*^6^	^2^S				
	
	*ns* *n* ≥5	*np* *n* ≥5	*nd* *n* ≥4	*nf* *n*≥ 4	*ng* *n*≥5
					
4*s*^2^ 4*p*^4^(^3^P)*nl*	$\{\begin{array}{c} {\;}^{4}P\\ {\;}^{2}P \end{array}$	^4^(SPD)°	^4^(PDF)	^4^(DFG)°	^4^(FGH)
		^2^(SPD)°	^2^(PDF)	^2^(DFG)°	^2^(FGH)
4*s*^2^ 4*p*^4^(^1^D)*nl*	^2^D	^2^(PDF)°	^2^(SPDFG)	^2^(PDFGH)°	^2^(DFGHI)
4*s*^2^ 4*p*^4^(^1^S)*nl*	^2^S	^2^P°	^2^D	^2^F°	^2^G

**Table 4:** Observed and calculated, positions for the energy levels of the *4p^4^* configuration in *Br II* Energies and coupling parameters are stated in kaysers

Level	Observed position	Obs.-Calc.	Obs.-Calc.
			
		F_2_= 1690	F_2_= 1698.1
		*ζ*_4_*_p_*=2800	*ζ*_4_*_p_*= 2795.0
^3^P_2_	0.0	0	0
^3^P_1_	3136.4	+6	+13
^3^P_0_	3837.5	−18	−14
^1^D_2_	12089.1	−111	−153
^1^S_0_	27867.1	+112	0

**Table 5:** Odd parity levels of *Br I*

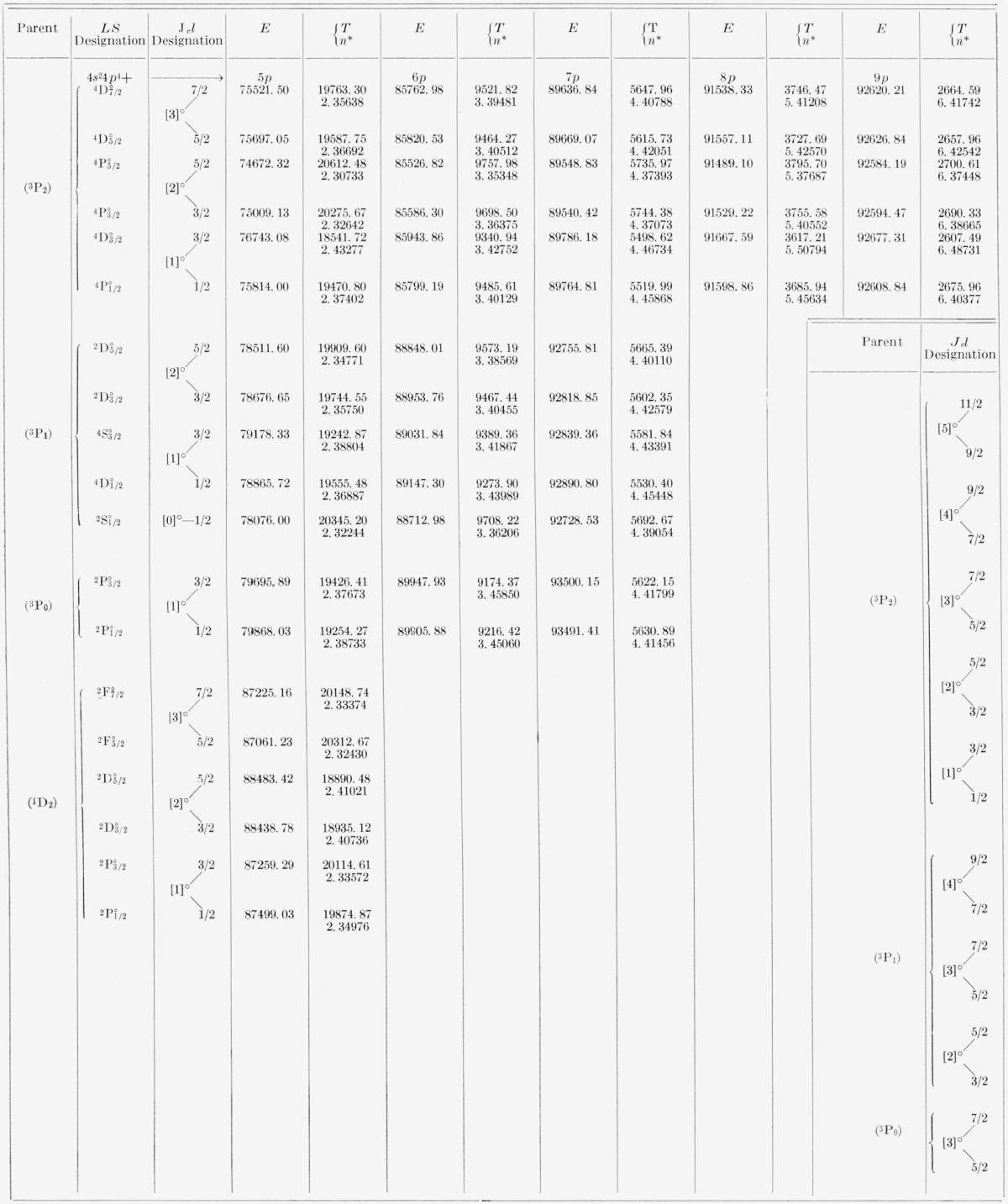
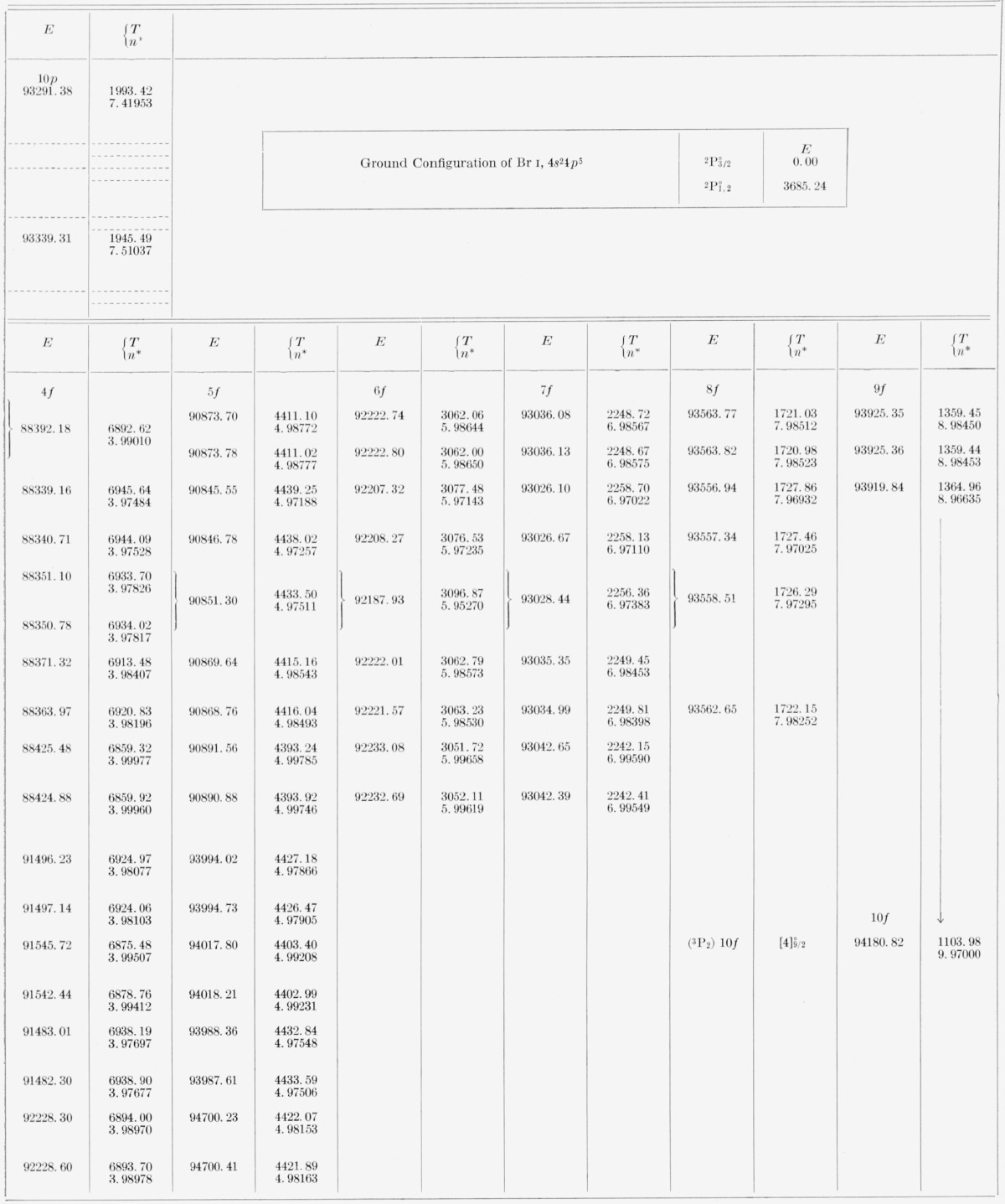

**Table 6:** Even parity levels of *Br I*

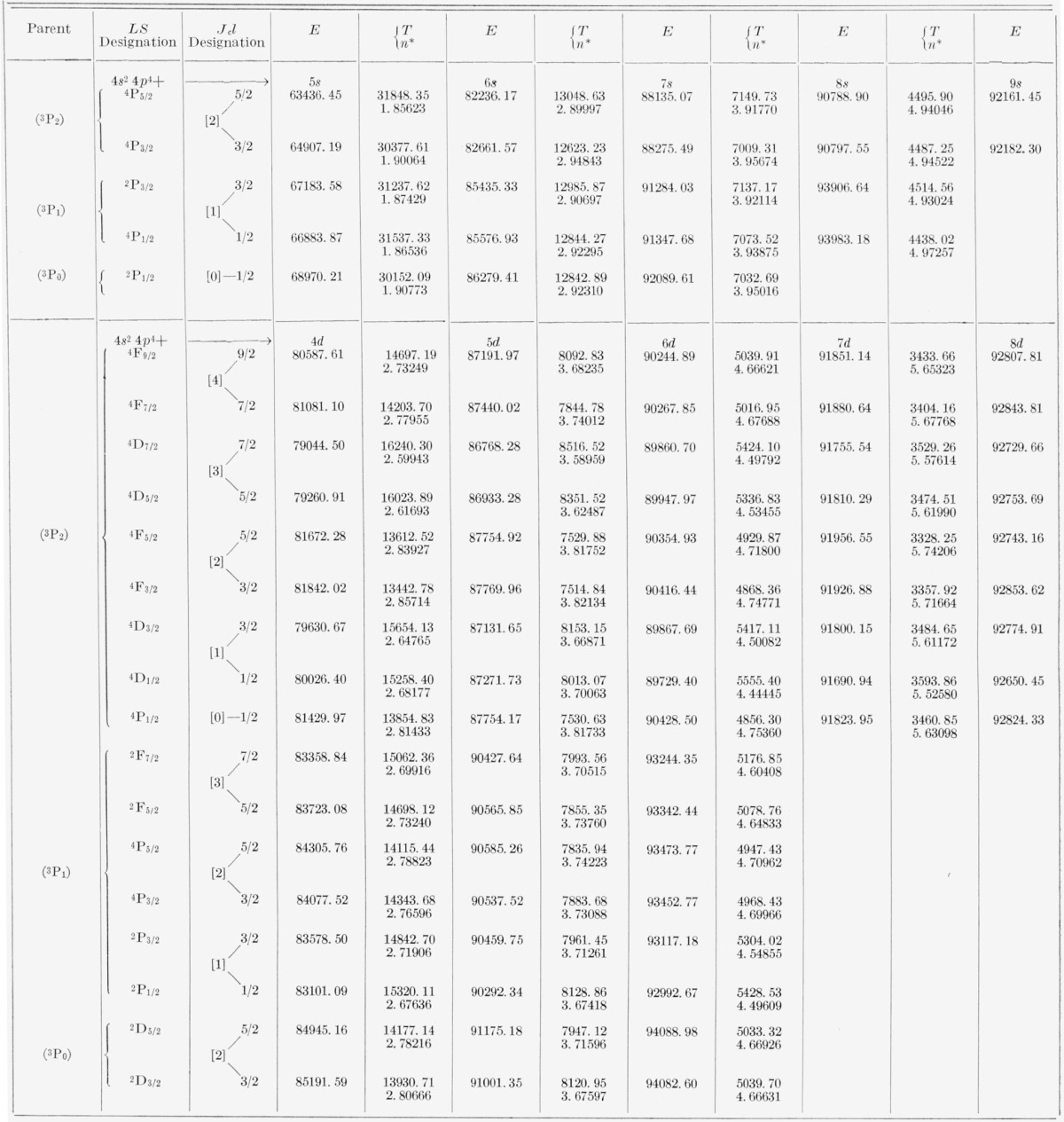
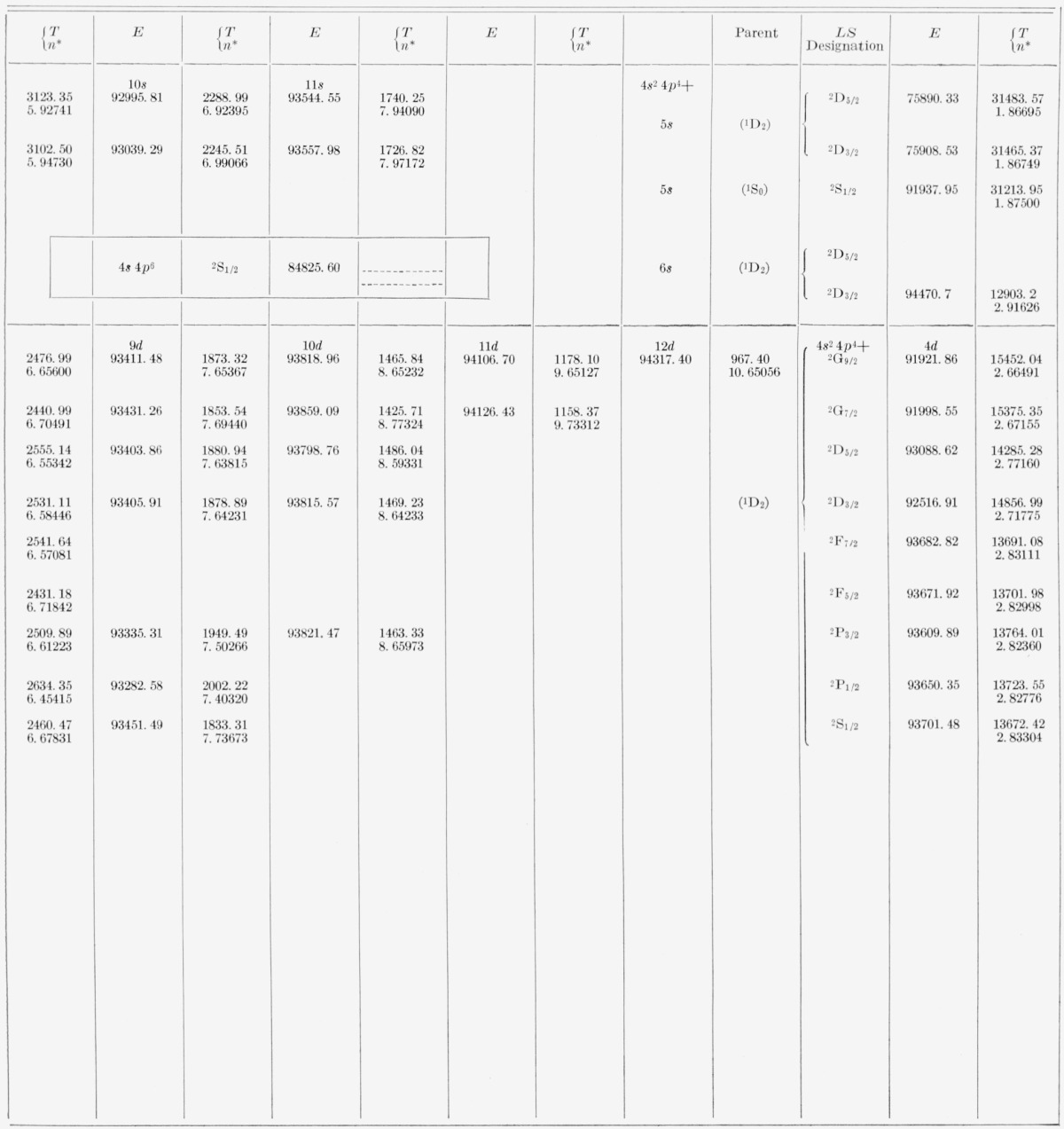

**Table 7:** Observed *Br I* $\left({\;}^{3}P_{2}\right)\text{nf}{\left[5\right]}_{11/2}^{{}^{\circ}}$ series members compared with positions calculated by use of the Ritz formula *α* = 0.016505 *β* =−9.585×10^−7^

Config.	Observed	Calc.	Obs.-calc.
			
4*f*	88392.18	88392.19	−0.01
5*f*	90873.70	90873.70	0.00
6*f*	92222.74	92222.73	+0.01
7*f*	93036.08	93036.07	+0.01
8*f*	93563.77	93563.78	−0.01
9*f*	93925.35	93925.44	−0.09

**Table 8:** Observed and calculated *Br I 4p^4^5s* energy levels All energies and parameters are stated in kaysers.

Level desig.	Observed position	F_2_(4*p*,4*p*) = 1698.1 G_1_(4*p*,5*s*) =733.8 *ζ*_4_*_p_*= 2795.0		F_2_(4*p*,4*p*) = 1698.0 G_1_(4*p*,5*_s_*) = 748.5 *ζ*_4_*_p_*=2712.0	
		Calc.	Obs-Calc.	Calc.	Obs-Calc.
					
(^3^P_2_) 5*s* ^4^P_5/2_	63436	63389	+ 47	63439	−3
(^3^P_2_) 5*s* ^4^P_3/2_	64907	64848	+ 59	64905	+ 2
(^3^P_1_) 5*s* ^4^P_1/2_	66884	66938	−54	66888	−4
(^3^P_1_)5*s* ^2^P_3/2_	67184	67185	−1	67173	+ 11
(^3^P_0_) 5*s* ^2^P_1/2_	68970	68984	−14	68977	−7
(^1^D_2_)5*s* ^2^D_5/2_	75890	76185	−295	76172	−282
(^1^D_2_)5*s* ^2^D_3/2_	75909	76239	−330	76225	−316
(^1^S_0_) 5*s* ^2^S_1/2_	91938	91975	−37	91938	0

**Table 9:** Eigenvectors of the *Br I* 4*p*^4^5*s* levels, calculated with *F*_2_(4*p*, 4*p*) = 1698.0, *G*_1_(4*p*, 5*s*) = 748.5, ζ_4p_= 2712

	(^1^D_2_) ^2^D_5/2_	(^3^P_2_) ^4^P_5/2_	
(^1^D_2_) ^2^D_5/2_	0.9883	0.1524	
(^3^P_2_) ^4^P_5/2_	−0.1524	0.9883	

	(^1^D_2_) ^2^D_3/2_	(^3^P_2_) ^4^P_3/2_	(^3^P_1_) ^2^P_3/2_
(^1^D_2_) ^2^D_3/2_	0.9837	0.1581	−0.0851
(^3^P_2_) ^4^P_3/2_	−0.0585	0.7302	0.6807
(^3^P_1_) ^2^P_3/2_	0.1698	−0.6646	0.7276

	(^3^P_2_) ^4^P_1/2_	(^3^P_0_) ^2^P_1/2_	(^1^S_0_) ^2^S_1/2_
(^3^P_1_) ^4^P_1/2_	0.9794	0.1561	0.1283
(^3^P_0_) ^3^P_1/2_	−0.1693	0.9805	0.0994
(^1^S_0_) ^2^S_1/2_	−0.1102	−0.1191	0.9867

The eigenvector of each level is given in the column under the adopted designation of the level. The designations on the extreme left refer to pure LS states. The elements of the eigenvectors are the expansion coefficients that occur in the corresponding eigenfunction. For example, the eigenfunction of the level called ^2^D_5/2_ is a linear combination of those for ^2^D_5/2_ and ^4^P_5/2_ and is given by
$\psi \left({“}^{2}D_{5/2}”\right)=0.9883\psi \left({\;}^{2}D_{5/2}\right)-0.1524\psi \left({\;}^{4}P_{5/2}\right).$ The ^2^D_5/2_ level thus has a purity of 97.7 percent in this approximation.

**Table 10:** Observed and calculated *g*-factors for the *Br I 4p^4^5s* levels

Level design.	Energy	*g*		
		Obs.	Calc.	Landé
				
	*K*			
(^3^P_1_) ^4^P_1/2_	66884	2.608	2.601	2.667
(^3^P_0_)^2^P_1/2_	68970	0.734	0.734	0.667
(^1^S_0_)^2^S_1/2_	91938	…………	(1.998)	(2.000)
		———	———	———
	*g*-sum =	3.342	3.335	3.333
				
(^3^P_2_) ^4^P_3/2_	64907	1.532	1.533	1.733
(^3^P_1_) ^2^P_3/2_	67184	1.522	1.515	1.333
(^1^D_2_) ^2^D_3/2_	75909	0.826	0.819	0.800
		———	———	———
	*g*-sum=	3.880	3.867	3.867
				
(^3^P_2_) ^4^P_5/2_	63436	1.595	1.591	1.600
(^1^D_2_) ^2^D_5/2_	75890	1.210	1.209	1.200
		———	———	———
	*g*-sum =	2.805	2.800	2.800

**Table 11:** Observed *Br I* 4*p*^4^6*s*, 7*s* energy levels compared with calculations using the simplified *J_c_s* matrices All energies and parameters are stated in kaysers.

Level design.	Observed position	Calc.	Obs.-Calc.	Parameters
				
(^3^P_2_)6*s* ^4^P_5/2_	82236	82199	+37	
(^3^P_2_) 6*s* ^4^P_3/2_	82662	82658	+ 4	
(^3^P_1_)6*s* ^4^P_1/2_	85577	85601	−24	B = 82199
(^3^P_1_) 6*s* ^2^P_3/2_	85435	85447	−12	G_1_ = 190
(^3^P_0_)6*s* ^2^P_1/2_	86279	86342	−63	
(^1^D_2_)6*s* ^2^D_3/2_	94471	94478	−7	

(^3^P_2_)7*s* ^4^P_5/2_	88135	88100	+35	
(^3^P_2_)7*s* ^4^P_3/2_	88275	88292	−17	B = 88100
(^3^P_1_)7*s* ^4^P_1/2_	91348	91373	−25	G_1_ = 78
(^3^P_1_)7*s* ^2^P_3/2_	91284	91278	+6	
(^3^P_0_)7*s* ^2^P_1/2_	92090	92035	+55	

**Table 12:** Observed energy levels of the 4*p*^4^(^3^*P*_2_)*nd* ^4^*F*_9/2_ series compared with calculations using the Ritz formula, as described in the text. All energies are stated in kaysers. *α*=1.35276 *β*= −3.442×10^−6^

Config.	Observed	Calculated	Obs.-calc.
			
4*d*	80587.61	80221.57	+ 366.04
5*d*	87191.97	87160.44	+ 31.53
6*d*	90244.89	90241.40	+ 3.49
*7d*	91851.14	91858.17	−7.03

4*p*^4^(^1^D_2_)4*d* ^2^G_9/2_ = 91921.86			

8*d*	92807.81	92807.63	+ 0.18
9*d*	93411.48	93411.49	−0.01
10*d*	93818.96	93818.95	+ 0.01
11*d*	94106.70	94106.70	0.00
12*d*	94317.40	94317.40	0.00

**Table 13:** Wavenumbers and intensities of the observed *(^3^P_2_)4d—(^3^P_2_)5f* transitions in *Br I*

4*d*→	[4] ^4^F_9/2_ 80587.61	[4] ^4^F_7/2_ 81081.10	[3] ^4^D_7/2_ 79044.50	[3] ^4^D_5/2_ 79260.91	[2] ^4^F_5/2_ 81672.28	[2] ^4^F_3/2_ 81842.02	[1] ^4^D_3/2_ 79630.67	[1] ^4^D_1/2_ 80026.40	[0] ^4^P_1/2_ 81429.97
5*f* ↓									
[5]°_11/2_	(600)								
90873.70	10286.09								
[5]°_9/2_		(300)							
90873.78		9792.68	—						

[4]°_9/2_	(100)		(500)						
90845.55	10257.95	—	11801.03						
[4 ]°_7/2_		(75)	(100)	(200)	(200)				
90846.78		9765.68	11802.27	11585.86	9174.50				

${\left[3\right]}_{7/2,5/2}^{{}^{\circ}}$	(15)	(15)	(300)	(100)	(250)	(20)	(300)		
90851.30	10263.69	9770.21	11806.78	11590.39	9179.02	9009.31	11220.60		

${\left[2\right]}_{5/2}^{{}^{\circ}}$			(75)	(250)	(100)	(40)	(20)		
90869.64		—	11825.12	11608.72	9197.37	9027.62	11238.92		
${\left[2\right]}_{3/2}^{{}^{\circ}}$				(75)			(150)	(200)	
90868.76				11607.87	—	—	11238.09	10842.35	—

${\left[1\right]}_{3/2}^{{}^{\circ}}$				(50)	(2 *w*)	(10)	(90)	(80*w*)	(250)
90891.56				11630.57	9219.28	9049.58	11260.85	10865.15	9461.59
${\left[1\right]}_{1/2}^{{}^{\circ}}$							(90)	(80)	(100)
90890.88						—	11260.20	10864.48	9460.95

**Table 14:** Comparison between observed and calculated *(^3^P) 4f* and *5f* levels

Level design.	4*f*		5*f*	
	Calc.	Obs.	Calc.	Obs.
				
(^3^P_2_) ${\left[5\right]}_{11/2}^{{}^{\circ}}$	88392.2	88392.2	90873.7	90873.7
${\left[5\right]}_{9/2}^{{}^{\circ}}$	392.3	392.2	873.8	873.8
${\left[4\right]}_{9/2}^{{}^{\circ}}$	339.3	339.2	846.4	845.6
${\left[4\right]}_{7/2}^{{}^{\circ}}$	340.4	340.7	847.3	846.8
${\left[3\right]}_{7/2}^{{}^{\circ}}$	347.9	351.1	851.1	} 851.3
${\left[3\right]}_{5/2}^{{}^{\circ}}$	348.5	350.8	851.5	
${\left[2\right]}_{5/2}^{{}^{\circ}}$	383.6	371.3	869.7	869.6
${\left[2\right]}_{3/2}^{{}^{\circ}}$	383.4	364.0	869.5	868.8
${\left[1\right]}_{3/2}^{{}^{\circ}}$	422.3	425.5	889.6	891.6
${\left[1\right]}_{1/2}^{{}^{\circ}}$	421.5	424.9	888.9	890.9
(^3^P_1_) ${\left[4\right]}_{9/2}^{{}^{\circ}}$	91497.7	91496.2	93994.0	93994.0
${\left[4\right]}_{7/2}^{{}^{\circ}}$	498.4	497.1	994.5	994.7
${\left[3\right]}_{7/2}^{{}^{\circ}}$	539.3	545.7	94015.6	94017.8
${\left[3\right]}_{5/2}^{{}^{\circ}}$	539.9	542.4	016.1	018.2
${\left[2\right]}_{5/2}^{{}^{\circ}}$	484.4	483.0	93987.3	93998.4
${\left[2\right]}_{3/2}^{{}^{\circ}}$	483.7	482.3	986.7	987.6
(^3^P_0_) ${\left[3\right]}_{7/2}^{{}^{\circ}}$	92209.5	92228.3	94700.7	94700.2
${\left[3\right]}_{5/2}^{{}^{\circ}}$	209.9	228.6	701.0	700.4

**Table 15:** Comparison between observed and calculated (^3^*P*_2_) 6*f* and 7*f* levels

Level design.	6*f*		7*f*	
	Calc.	Obs.	Calc.	Obs.
				
(^3^P_2_) ${\left[5\right]}_{11/2}^{{}^{\circ}}$	92222.7	92222.7	93036.1	93036.1
${\left[5\right]}_{9/2}^{{}^{\circ}}$	222.8	222.8	036.1	036.1
${\left[4\right]}_{9/2}^{{}^{\circ}}$	206.9	207.3	026.1	026.1
${\left[4\right]}_{7/2}^{{}^{\circ}}$	207.4	208.3	026.4	026.7
${\left[3\right]}_{7/2}^{{}^{\circ}}$	209.6	} 187.9	027.8	} 028.4
${\left[3\right]}_{5/2}^{{}^{\circ}}$	209.9		028.0	
${\left[2\right]}_{5/2}^{{}^{\circ}}$	220.5	222.0	034.7	035.4
${\left[2\right]}_{3/2}^{{}^{\circ}}$	220.4	221.6	034.6	035.0
${\left[1\right]}_{3/2}^{{}^{\circ}}$	232.0	233.1	041.9	042.6
${\left[1\right]}_{1/2}^{{}^{\circ}}$	231.6	232.7	041.7	042.4

**Table 16:** The *B* and *F*^2^ parameters for 4*f*, 5*f*, 6*f*, and 7*f* configurations, calculated as explained in the text

Conf.	B	F^2^	F^2^× *n*^3^
			
4*f*	88371.24	314.1	201×10^2^
5*f*	90862.84	162.9	204
6*f*	92216.42	94.8	205
7*f*	93032.09	59.9	205

## Footnotes

1 Figures in brackets indicato the literature references at the end of this paper.

2 Throughout this paper we use the name kayser, with symbol K, for the unit of wave number cm^−1^.

3 15730.1 Å. This is the only impurity line found on the tracings; it occurred with intensity 5 on our scale. According to the recent work of Humphreys and Paul [13], it is the second strongest Cl line in the region studied. The strongest Cl line, at 15869.7 Å, was masked by a Br line of another order.

4 These electrostatic expressions differ by a constant amount from those given on p. 199 in TAS, which are not entirely correct. See footnote on page 102 of reference [22].

5 The difficulty rests on the fact that three pairs of real odd levels in Br I have almost the same separation, *equal to the above difference* Δ. They are

$$\begin{array}{l} \left({\;}^{3}P_{2}\right)7p{\;}^{4}D_{3/2}^{{}^{\circ}}\;89786.2-\left({\;}^{3}P_{2}\right)4f{\left[2\right]}_{3/2}^{{}^{\circ}}88364.0=1422.2K\\ \left({\;}^{1}D_{2}\right)5p{\;}^{2}D_{5/2}^{{}^{\circ}}\;88483.4-\left({\;}^{1}D_{2}\right)5p{\;}^{2}F_{5/2}^{{}^{\circ}}87061.2=1422.2K\\ \left({\;}^{3}P_{0}\right)6p{\;}^{2}P_{1/2}^{{}^{\circ}}\;89905.9-\left({\;}^{1}D_{2}\right)5p{\;}^{2}D_{5/2}^{{}^{\circ}}88483.4=1422.5K \end{array}$$

Owing to the appearance of the 88483.4 level in two of the pairs, the line at 7950.2 Å falls at a “junction” in Kiess and de Bruin’s system, so that it could be, and indeed was, classified twice by them. The entire puzzle is compounded by the fact that the real pairs of levels,

$$\begin{array}{l} \left({\;}^{3}P_{0}\right)5s{\;}^{2}P_{1/2}68970.2-\left({\;}^{3}P_{1}\right)5s{\;}^{2}P_{3/2}67183.6=1786.6\;K\\ \left({\;}^{3}P_{1}\right)6p{\;}^{2}D_{5/2}^{{}^{\circ}}88848.0-\left({\;}^{1}D_{2}\right)5p{\;}^{2}F_{5/2}^{{}^{\circ}}87061.2=1786.8\;K, \end{array}$$

have separations almost equal to Δ= 1422.2 added to the separation

$$\left({\;}^{3}P_{1}\right)6p{\;}^{2}D_{5/2}^{{}^{\circ}}88848.0-\left({\;}^{1}D_{2}\right)5p{\;}^{2}D_{5/2}^{{}^{\circ}}88483.4=364.6.$$

6 Two typographical errors appear in those matrices: In the *J*=3/2 matrix the off-diagonal $\left(-\zeta_{p}/\sqrt{2}\right)$ element should connect the levels based on the (^3^P_2_) — (^1^D_2_) parents rather than (^3^P_1_) — (^1^D_2_), which should be zero. Further, the coefficient of G^1^ in the diagonal element for the J = 1/2 level based on (^3^P_1_) should be 2/3 rather than 1/3.

7 It should also be mentioned that a factor of 2/3 is missing from this equation in Slater’s work, Vol. II, p. 266.

## 9. Appendix

**Table A1:** Wavelengths of *Br I* in the vacuum ultraviolet

Observed wave-length	Calc. In wave-length	Intensity	Observed wave number	Classification
				
Å			*K*	
1067.559	0.556	10	93671.6	4*p*^5^ ${\;}^{2}P_{3/2}^{{}^{\circ}}$ *—* (^1^D_2_)4*d* ^2^F_5/2_
1067.805	.802	15	93650.1	4*p*^5^ ${\;}^{2}P_{3/2}^{{}^{\circ}}$ *—* (^1^D_2_)4*d* ^2^P_1/2_
1068.256	.263	10	93610.5	4*p*^5^ ${\;}^{2}P_{3/2}^{{}^{\circ}}$ *—* (^1^D_2_)4*d* ^2^P_3/2_
1068.849	.856	3	93558.6	4*p*^5^ ${\;}^{2}P_{3/2}^{{}^{\circ}}$ *—* 11*s* ^4^P_3/2_
1073.912	.916	70	93117.5	4*p*^5^ ${\;}^{2}P_{3/2}^{{}^{\circ}}$ *—* 6*d*′ ^2^P_3/2_
1074.243	.245	70	93088.8	4*p*^5^ ${\;}^{2}P_{3/2}^{{}^{\circ}}$ *—* (^1^D_2_) 4*d* ^2^D_5/2_
1074.803	.815	10	93040.3	4*p*^5^ ${\;}^{2}P_{3/2}^{{}^{\circ}}$ *—* 10*s* ^4^P_3/2_
1075.345	$\left\{ \begin{array}{l} .317\\ .354 \end{array}\right.$	15	92993.4	$\left\{ \begin{array}{l} 4p^{5}{\;}^{2}P_{3/2}^{{}^{\circ}}—10s{\;}^{4}P_{5/2}\\ 4p^{5}{\;}^{2}P_{3/2}^{{}^{\circ}}—6d\prime {\;}^{2}P_{1/2} \end{array}\right.$
1070.964	.964	20	92853.6	4*p*^5^ ${\;}^{2}P_{3/2}^{{}^{\circ}}$ *—* 8*d* ^4^F_3/2_
1077.873	.878	15	92775.3	4*p*^5^ ${\;}^{2}P_{3/2}^{{}^{\circ}}$ *—* 8*d* ^4^D_3/2_
1078.124	.124	15	92753.7	4*p*^5^ ${\;}^{2}P_{3/2}^{{}^{\circ}}$ *—* 8*d* ^4^D_5/2_
1078.242	.247	30	92743.6	4*p*^5^ ${\;}^{2}P_{3/2}^{{}^{\circ}}$ *—* 8*d* ^4^F_5/2_
1079.320	.326	30	92650.9	4*p*^5^ ${\;}^{2}P_{3/2}^{{}^{\circ}}$ *—* 8*d* ^4^D_1/2_
1080.882	.883	50	92517.0	4*p*^5^ ${\;}^{2}P_{3/2}^{{}^{\circ}}$ *—* (^1^D_2_) 4*d* ^2^D_3/2_
1084.810	.807	10	92182.0	4*p*^5^ ${\;}^{2}P_{3/2}^{{}^{\circ}}$ *—* 9*s* ^4^P_3/2_
1085.050	.052	15	92161.6	4*p*^5^ ${\;}^{2}P_{3/2}^{{}^{\circ}}$ *—* 9*s* ^4^P_5/2_
1085.896	.899	5	92089.8	4*p*^5^ ${\;}^{2}P_{3/2}^{{}^{\circ}}$ *—* 7*s*″ ^2^P_1/2_
1087.468	.470	30	91956.7	4*p*^5^ ${\;}^{2}P_{3/2}^{{}^{\circ}}$ *—* 7*d* ^4^F_5/2_
1087.687	.690	40	91938.2	4*p*^5^ ${\;}^{2}P_{3/2}^{{}^{\circ}}$ *—* (^1^S_0_) 5*s* ^2^S_1/2_
1087.819	.821	20	91927.1	4*p*^5^ ${\;}^{2}P_{3/2}^{{}^{\circ}}$ *—* 7*d* ^4^F_3/2_
1089.039	.040	30	91824.1	4*p*^5^ ${\;}^{2}P_{3/2}^{{}^{\circ}}$ *—* 7*d* ^4^P_1/2_
1089.203	.203	15	91810.2	4*p*^5^ ${\;}^{2}P_{3/2}^{{}^{\circ}}$ *—* 7*d* ^4^D_5/2_
1089.322	.323	20	91800.2	4*p*^5^ ${\;}^{2}P_{3/2}^{{}^{\circ}}$ *—* 7*d* ^4^D_3/2_
1090.623	.620	85	91690.7	4*p*^5^ ${\;}^{2}P_{3/2}^{{}^{\circ}}$ *—* 7*d* ^4^D_1/2_
1094.722	.719	100	91347.4	4*p*^5^ ${\;}^{2}P_{3/2}^{{}^{\circ}}$ *—* 7*s*′ ^4^P_1/2_
1095.481	.482	100	91284.1	4*p*^5^ ${\;}^{2}P_{3/2}^{{}^{\circ}}$ *—* 7*s*′ ^2^P_3/2_
1096.788	.790	100	91175.3	4*p*^5^ ${\;}^{2}P_{3/2}^{{}^{\circ}}$ *—* 5*d*″ ^2^D_5/2_
1098.881	.885	100	91001.7	4*p*^5^ ${\;}^{2}P_{3/2}^{{}^{\circ}}$ *—* 5*d*″ ^2^D_3/2_
1101.347	.351	50	907979	4*p*^5^ ${\;}^{2}P_{3/2}^{{}^{\circ}}$ *—* 8*s* ^4^P_3/2_
a1101.456	**………**	**………**	90788.9	4*p*^5^ ${\;}^{2}P_{3/2}^{{}^{\circ}}$ *—* 8*s* ^4^P_5/2_
1101.498	**………**	250	90785.5	4*p*^5^ ${\;}^{2}P_{1/2}^{{}^{\circ}}$ *—* (^1^D_2_) 6*s* ^2^D_3/2_
1103.924	.932	60	90585.9	4*p*^5^ ${\;}^{2}P_{3/2}^{{}^{\circ}}$ *—* 5*d*′ ^4^P_5/2_
1104.168	.169	60	90565.9	4*p*^5^ ${\;}^{2}P_{3/2}^{{}^{\circ}}$ *—* 5*d*′ ^2^F_5/2_
1105.460	.464	50	90460.1	4*p*^5^ ${\;}^{2}P_{3/2}^{{}^{\circ}}$ *—* 5*d*′ ^2^P_3/2_
1105.844	.846	50	90428.7	4*p*^5^ ${\;}^{2}P_{3/2}^{{}^{\circ}}$ *—* 6*d* ^4^P_1/2_
1105.994	.994	70	90416.4	4*p*^5^ ${\;}^{2}P_{3/2}^{{}^{\circ}}$ *—* 6*d* ^4^F_3/2_
1107.442	.445	50	90298.2	4*p*^5^ ${\;}^{2}P_{1/2}^{{}^{\circ}}$ *—* 8*s*′ ^4^P_1/2_
1107.512	.514	50	90292.5	4*p*^5^ ${\;}^{2}P_{3/2}^{{}^{\circ}}$ *—* 5*d*′ ^2^P_1/2_
1109.422	.432	20	90137.0	4*p*^5^ ${\;}^{2}P_{1/2}^{{}^{\circ}}$ *—* 10*d* ^4^D_3/2_
1110.904	.911	100	90016.8	4*p*^5^ ${\;}^{2}P_{1/2}^{{}^{\circ}}$ *—* (^1^D_2_) 4*d* ^4^S_1/2_
b1111.579	.542	50	89962.1	4*p*^5^ ${\;}^{2}P_{1/2}^{{}^{\circ}}$ *—* (^1^D_2_) 4*d* ^2^P_1/2_
1111.751	.754	40	89948.2	4*p*^5^ ${\;}^{2}P_{3/2}^{{}^{\circ}}$ *—* 6*d* ^4^D_5/2_
1112.743	.747	20	89868.0	4*p*^5^ ${\;}^{2}P_{3/2}^{{}^{\circ}}$ *—* 6*d* ^4^D_3/2_
1115.448	.448	30	89650.1	4*p*^5^ ${\;}^{2}P_{1/2}^{{}^{\circ}}$ *—* 9*d* ^4^D_3/2_
1116.105	.105	40	89597.3	4*p*^5^ ${\;}^{2}P_{1/2}^{{}^{\circ}}$ *—* 9*d* ^4^D_1/2_
1118.173	.169	90	89431.6	4*p*^5^ ${\;}^{2}P_{1/2}^{{}^{\circ}}$ *—* 6*d*′ ^2^P_3/2_
1119.140	.143	60	89354.3	4*p*^5^ ${\;}^{2}P_{1/2}^{{}^{\circ}}$ *—* 10*s* ^4^P_3/2_
1119.725	.728	90	89307.6	4*p*^5^ ${\;}^{2}P_{1/2}^{{}^{\circ}}$ *—* 6*d′* ^2^P_1/2_
1121.473	.474	120	89168.4	4*p*^5^ ${\;}^{2}P_{1/2}^{{}^{\circ}}$ *—* 8*d* ^4^F_3/2_
1121.839	.842	80	89139.4	4*p*^5^ ${\;}^{2}P_{1/2}^{{}^{\circ}}$ *—* 8*d* ^4^P_1/2_
1124.038	.035	120	88965.0	4*p*^5^ ${\;}^{2}P_{1/2}^{{}^{\circ}}$ *—* 8*d* ^4^D_1/2_
1125.728	.725	200	88831.4	4*p*^5^ ${\;}^{2}P_{1/2}^{{}^{\circ}}$ *—* (^1^D_2_) 4*d* ^2^D_3/2_
1129.979	.981	35	88497.2	4*p*^5^ ${\;}^{2}P_{1/2}^{{}^{\circ}}$ *—* 9*s* ^4^P_3/2_
1131.171	.166	80	88404.0	4*p*^5^ ${\;}^{2}P_{1/2}^{{}^{\circ}}$ *—* 7*s*″ ^2^P_1/2_
1132.822	.817	100	88275.1	4*p*^5^ ${\;}^{2}P_{3/2}^{{}^{\circ}}$ *—* 7*s* ^4^P_3/2_
1133.116	0.110	200	88252.2	4*p*^5^ ${\;}^{2}P_{1/2}^{{}^{\circ}}$ *—* (^1^S_0_) 5*s* ^2^S_1/2_
1133.251	.252	80	88241.7	4*p*^5^ ${\;}^{2}P_{1/2}^{{}^{\circ}}$ *—* 7*d* ^4^F_3/2_
1134.588	$\left\{ \begin{array}{l} .575\\ .622 \end{array}\right.$	300	88137.7	$\left\{ \begin{array}{l} 4p^{5}{\;}^{2}P_{1/2}^{{}^{\circ}}—7d{\;}^{4}P_{1/2}\\ 4p^{5}{\;}^{2}P_{3/2}^{{}^{\circ}}—7s{\;}^{4}P_{5/2} \end{array}\right.$
1134.888	.882	200	88114.4	4*p*^5^ ${\;}^{2}P_{1/2}^{{}^{\circ}}$ *—* 7*d* ^4^D_3/2_
1136.294	.290	250	88005.4	4*p*^5^ ${\;}^{2}P_{1/2}^{{}^{\circ}}$ *—* 7*d* ^4^D_1/2_
1139.350	.342	100	87769.3	4*p*^5^ ${\;}^{2}P_{3/2}^{{}^{\circ}}$ *—* 5*d* ^4^F_3/2_
1139.544	$\left\{ \begin{array}{l} .537\\ .547 \end{array}\right.$	120	87754.4	$\left\{ \begin{array}{l} 4p^{5}{\;}^{2}P_{3/2}^{{}^{\circ}}—5d{\;}^{4}F_{5/2}\\ 4p^{5}{\;}^{2}P_{3/2}^{{}^{\circ}}—5d{\;}^{4}P_{1/2} \end{array}\right.$
1140.732	.739	20	87663.0	4*p*^5^ ${\;}^{2}P_{1/2}^{{}^{\circ}}$ *—* 7*s*′ ^4^P_1/2_
1141.564	.568	25	87599.1	4*p*^5^ ${\;}^{2}P_{1/2}^{{}^{\circ}}$ *—* 7*s*′ ^2^P_3/2_
1145.268	.264	80	87315.8	4*p*^5^ ${\;}^{2}P_{1/2}^{{}^{\circ}}$ *—* 5*d*″ ^2^D_3/2_
1145.854	.846	60	87271.2	4*p*^5^ ${\;}^{2}P_{3/2}^{{}^{\circ}}$ *—* 5*d* ^4^D_1/2_
1147.689	.689	80	87131.6	4*p*^5^ ${\;}^{2}P_{3/2}^{{}^{\circ}}$ *—* 5*d* ^4^D_3/2_
1147.943	.943	60	87112.3	4*p*^5^ ${\;}^{2}P_{1/2}^{{}^{\circ}}$ *—* 8*s* ^4^P_3/2_
1150.312	.307	80	86932.9	4*p*^5^ ${\;}^{2}P_{3/2}^{{}^{\circ}}$ *—* 5*d* ^4^D_5/2_
1151.381	.380	100	86852.2	4*p*^5^ ${\;}^{2}P_{1/2}^{{}^{\circ}}$ *—* 5*d*′ ^4^P_3/2_
1152.418	.412	100	86774.1	4*p*^5^ ${\;}^{2}P_{1/2}^{{}^{\circ}}$ *—* 5*d*′ ^2^P_3/2_
1152.833	.827	40	86742.8	4*p*^5^ ${\;}^{2}P_{1/2}^{{}^{\circ}}$ *—* 6*d* ^4^P_1/2_
1152.989	.988	60	86731.1	4*p*^5^ ${\;}^{2}P_{1/2}^{{}^{\circ}}$ *—* 6*d* ^4^F_3/2_
1154.640	.640	15	86607.1	4*p*^5^ ${\;}^{2}P_{1/2}^{{}^{\circ}}$ *—* 5*d*′ ^2^P_1/2_
1159.030	.025	80	86279.0	4*p*^5^ ${\;}^{2}P_{3/2}^{{}^{\circ}}$ *—* 6*s*″ ^2^P_1/2_
1160.332	.329	100	86182.2	4*p*^5^ ${\;}^{2}P_{1/2}^{{}^{\circ}}$ *—* 6*d* ^4^D_3/2_
1168.542	.539	150	85576.7	4*p*^5^ ${\;}^{2}P_{3/2}^{{}^{\circ}}$ *—* 6*s′* ^4^P_1/2_
1170.479	.476	150	85435.1	4*p*^5^ ${\;}^{2}P_{3/2}^{{}^{\circ}}$ *—* 6*s′* ^2^P_3/2_
1173.827	.825	175	85191.4	4*p*^5^ ${\;}^{2}P_{3/2}^{{}^{\circ}}$ *—* 4*d*″ ^2^D_3/2_
1177.233	.230	250	84945.0	4*p*^5^ ${\;}^{2}P_{3/2}^{{}^{\circ}}$ *—* 4*d*″ ^2^D_5/2_
1178.895	.889	400	84825.2	4*p*^5^ ${\;}^{2}P_{3/2}^{{}^{\circ}}$ *—* 4*s*4*p*^6 2^S_1/2_
1182.171	.169	150	84590.1	4*p*^5^ ${\;}^{2}P_{1/2}^{{}^{\circ}}$ *—* 7*s* ^4^P_3/2_
1186.161	.159	150	84305.6	4*p*^5^ ${\;}^{2}P_{3/2}^{{}^{\circ}}$ *—* 4*d*′ ^4^P_5/2_
1189.279	.277	1000	84084.6	4*p*^5^ ${\;}^{2}P_{1/2}^{{}^{\circ}}$ *—* 5*d* ^4^F_3/2_
1189.378	.379	250	84077.6	4*p*^5^ ${\;}^{2}P_{3/2}^{{}^{\circ}}$ *—* 4*d*′ ^4^P_3/2_
1189.498	.500	1000	84069.1	4*p*^5^ ${\;}^{2}P_{1/2}^{{}^{\circ}}$ *—* 5*d* ^4^P_1/2_
1194.413	.414	200	83723.1	4*p*^5^ ${\;}^{2}P_{3/2}^{{}^{\circ}}$ *—* 4*d*′ ^2^F_5/2_
1196.370	.366	200	83586.2	4*p*^5^ ${\;}^{2}P_{1/2}^{{}^{\circ}}$ *—* 5*d* ^4^D_1/2_
1196.477	.480	200	83578.7	4*p*^5^ ${\;}^{2}P_{3/2}^{{}^{\circ}}$ *—* 4*d*′ ^2^P_3/2_
1198.371	.374	500	83446.6	4*p*^5^ ${\;}^{2}P_{1/2}^{{}^{\circ}}$ *—* 5*d* ^4^D_3/2_
1203.353	.354	200	83101.1	4*p*^5^ ${\;}^{2}P_{3/2}^{{}^{\circ}}$ *—* 4*d*′ ^2^P_3/2_
1209.756	.752	800	82661.3	4*p*^5^ ${\;}^{2}P_{3/2}^{{}^{\circ}}$ *—* 6*s* ^4^P_3/2_
1210.734	.739	1000	82594.5	4*p*^5^ ${\;}^{2}P_{1/2}^{{}^{\circ}}$ *—* 6*s*″ ^2^P_1/2_
1216.006	.010	750	82236.4	4*p*^5^ ${\;}^{2}P_{3/2}^{{}^{\circ}}$ *—* 6*s* ^4^P_5/2_
1221.128	.125	1000	81891.5	4*p*^5^ ${\;}^{2}P_{1/2}^{{}^{\circ}}$ *—* 6*s*′ ^4^P_1/2_
1221.870	.866	900	81841.8	4*p*^5^ ${\;}^{2}P_{3/2}^{{}^{\circ}}$ *—* 4*d* ^4^F_3/2_
1223.240	.240	1000	81750.1	4*p*^5^ ${\;}^{2}P_{1/2}^{{}^{\circ}}$ *—* 6*s*′ ^2^P_3/2_
1224.408	.406	1200	81672.1	4*p*^5^ ${\;}^{2}P_{3/2}^{{}^{\circ}}$ *—* 4*d* ^4^F_5/2_
1226.899	.898	1200	81506.3	4*p*^5^ ${\;}^{2}P_{1/2}^{{}^{\circ}}$ *—* 4*d*″ ^2^D_3/2_
1228.049	.049	750	81430.0	4*p*^5^ ${\;}^{2}P_{3/2}^{{}^{\circ}}$ *—* 4*d* ^4^P_1/2_
1232.431	.432	7500	81140.4	4*p*^5^ ${\;}^{2}P_{1/2}^{{}^{\circ}}$ *—* 4*s*4*p*^6 2^S_1/2_
1243.897	.901	1200	80392.5	4*p*^5^ ${\;}^{2}P_{1/2}^{{}^{\circ}}$ *—* 4*d*′ ^4^P_3/2_
1249.589	.588	800	80026.3	4*p*^5^ ${\;}^{2}P_{3/2}^{{}^{\circ}}$ *—* 4*d* ^4^D_1/2_
1251.664	.670	1500	79893.6	4*p*^5^ ${\;}^{2}P_{1/2}^{{}^{\circ}}$ *—* 4*d*′ ^2^P_1/2_
1255.799	.798	1000	79630.6	4*p*^5^ ${\;}^{2}P_{3/2}^{{}^{\circ}}$ *—* 4*d* ^4^D_3/2_
1259.199	.194	1500	79415.6	4*p*^5^ ${\;}^{2}P_{1/2}^{{}^{\circ}}$*—* 4*d*′ ^2^P_1/2_
1261.658	.656	1200	79260.8	4*p*^5^ ${\;}^{2}P_{3/2}^{{}^{\circ}}$ *—* 4*d* ^4^D_5/2_
1266.200	.202	1200	789765	4*p*^5^ ${\;}^{2}P_{1/2}^{{}^{\circ}}$ *—* 6*s* ^4^P_3/2_
1279.477	.480	1000	78156.9	4*p*^5^ ${\;}^{2}P_{1/2}^{{}^{\circ}}$ *—* 4*d* ^4^F_3/2_
1286.259	.261	1000	77744.8	4*p*^5^ ${\;}^{2}P_{1/2}^{{}^{\circ}}$ *—* 4*d* ^4^P_1/2_
1309.908	0.909	3000	76341.2	4*p*^5^ ${\;}^{2}P_{1/2}^{{}^{\circ}}$ *—* 4*d* ^4^D_1/2_
1316.735	.735	3000	75945.4	4*p*^5^ ${\;}^{2}P_{1/2}^{{}^{\circ}}$ *—* 4*d* ^4^D_3/2_
1317.372	.375	1000	75908.7	4*p*^5^ ${\;}^{2}P_{3/2}^{{}^{\circ}}$ *—* (^1^D_2_) 5*s* ^2^D_3/2_
1317.695	.691	2000	75890.1	4*p*^5^ ${\;}^{2}P_{3/2}^{{}^{\circ}}$ *—* (^1^D_2_) 5*s* ^2^D_5/2_
1384.598	.595	12000	72223.1	4*p*^5^ ${\;}^{2}P_{1/2}^{{}^{\circ}}$ *—* (^1^D_2_) 5*s* ^2^D_3/2_
1449.903	.901	3000	68970.1	4*p*^5^ ${\;}^{2}P_{3/2}^{{}^{\circ}}$ *—* 5*s*″ ^2^P_1/2_
1488.452	.459	50000	67183.9	4*p*^5^ ${\;}^{2}P_{3/2}^{{}^{\circ}}$ *—* 5*s*′ ^2^P_3/2_
1495.132	.129	5	66883.7	4*p*^5^ ${\;}^{2}P_{3/2}^{{}^{\circ}}$ *—* 5*s*′ ^4^P_1/2_
1531.743	.746	30000	65285.1	4*p*^5^ ${\;}^{2}P_{1/2}^{{}^{\circ}}$ *—* 5*s*″ ^2^P_1/2_
1540.654	.661	25000	64907.5	4*p*^5^ ${\;}^{2}P_{3/2}^{{}^{\circ}}$ *—* 5*s* ^4^P_3/2_
1574.841	0.844	30000	63498.5	4*p*^5^ ${\;}^{2}P_{1/2}^{{}^{\circ}}$ *—* 5*s*′ ^2^P_3/2_
1576.387	.381	20000	63436.2	4*p*^5^ ${\;}^{2}P_{3/2}^{{}^{\circ}}$ *—* 5*s* ^4^P_5/2_
1582.312	.313	25000	63198.7	4*p*^5^ ${\;}^{2}P_{1/2}^{{}^{\circ}}$ *—* 5*s*′ ^4^P_1/2_
1633.404	.401	75000	61221.8	4*p*^5^ ${\;}^{2}P_{1/2}^{{}^{\circ}}$ *—* 5*s* ^4^P_3/2_

a λ1101.456 computed wavelength. The line is masked by λ 1101.498.

b λ1111.579 this may be a Br II line.

**Table A2:** Observed lines of *Br I*

Wavelength	Intensity	Wave number	Classification
*Å*		*K*	
3325.307	10 *w*	30063.76	5*s* ^4^P_5/2_ — 7*p*″ ${\;}^{2}P_{3/2}^{{}^{\circ}}$
3348.566	15	29854.95	5*s* ^4^P_5/2_ — 10*p* ${\;}^{4}D_{7/2}^{{}^{\circ}}$
3400.030 3400.060	$\begin{array}{l} \;\\ \; \end{array}\}\begin{array}{c} 25\\ 20 \end{array}$	29403.07 29402.81	$\begin{array}{l} \;\\ \; \end{array}\}\;5s{\;}^{4}P_{5/2}—7p\prime {\;}^{4}S_{3/2}^{{}^{\circ}}$
3402.411 3402.436	$\begin{array}{l} \;\\ \;\\ \; \end{array}\}\begin{array}{c} 15\\ \begin{array}{l} \;\\ 10 \end{array} \end{array}$	29382.49 29382.28	$\begin{array}{l} \;\\ \;\\ \; \end{array}\}5s{\;}^{4}P_{5/2}—7p\prime {\;}^{2}D_{3/2}^{{}^{\circ}}$
3409.728 3409.753	$\begin{array}{l} \;\\ \; \end{array}\}\begin{array}{c} 15\\ 10 \end{array}$	29319.44 29319.23	$\begin{array}{l} \;\\ \; \end{array}\}\;5s{\;}^{4}P_{5/2}—7p\prime {\;}^{2}D_{5/2}^{{}^{\circ}}$
3418.888	15*w*	29240.89	5*s* ^4^P_5/2_ — 9*p* ${\;}^{4}D_{3/2}^{{}^{\circ}}$
3425.577	60	29183.79	5*s* ^4^P_5/2_ — 9*p* ${\;}^{4}D_{7/2}^{{}^{\circ}}$
3428.605	15	29158.02	5*s* ^4^P_5/2_ — 9*p* ${\;}^{4}P_{3/2}^{{}^{\circ}}$
3429.809	8	29147.79	5*s* ^4^P_5/2_ — 9*p* ${\;}^{4}P_{5/2}^{{}^{\circ}}$
3472.188 3472.216	$\begin{array}{l} \;\\ \; \end{array}\}\begin{array}{c} 20\\ 10 \end{array}$	28792.04 28791.81	$\begin{array}{l} \;\\ \; \end{array}\}\;5s{\;}^{4}P_{5/2}—4f\prime \prime {\left[3\right]}_{7/2}^{{}^{\circ}}$
3496.366	5	28592.94	5*s* ^4^P_3/2_ — 7*p*″ ${\;}^{2}P_{3/2}^{{}^{\circ}}$
3497.433	1	28584.22	5*s* ^4^P_3/2_ — 7*p*″ ${\;}^{2}P_{1/2}^{{}^{\circ}}$
3516.144	40	28432.12	5*s* ^4^P_5/2_ — 10*p* ${\;}^{4}D_{3/2}^{{}^{\circ}}$
3541.173	100	28231.16	5*s* ^4^P_5/2_ — 8*p* ${\;}^{4}D_{3/2}^{{}^{\circ}}$
3556.518 3556.540	$\begin{array}{l} \;\\ \; \end{array}\}\begin{array}{c} 40\\ 30 \end{array}$	28109.36 28109.19	$\begin{array}{l} \;\\ \; \end{array}\}\;5s{\;}^{4}P_{5/2}—4f\prime {\left[3\right]}_{7/2}^{{}^{\circ}}$
3557.461	250	28101.91	5*s* ^4^P_5/2_ — 8*p* ${\;}^{4}D_{7/2}^{{}^{\circ}}$
3558.616	75	28092.79	5*s* ^4^P_5/2_ — 8*p* ${\;}^{4}P_{3/2}^{{}^{\circ}}$
3563.706	50	28052.66	5*s* ^4^P_5/2_ — 8*p* ${\;}^{4}P_{5/2}^{{}^{\circ}}$
3564.468 3564.498	$\begin{array}{l} \;\\ \; \end{array}\}\begin{array}{c} 15w\\ 10 \end{array}$	28046.67 28046.43	$\begin{array}{l} \;\\ \; \end{array}\}\;5s{\;}^{4}P_{5/2}—4f\prime {\left[2\right]}_{5/2}^{{}^{\circ}}$
3579.081	40	27932.16	5*s* ^4^P_3/2_ — 7*p*′ ${\;}^{4}S_{3/2}^{{}^{\circ}}$
3581.712	15	27911.64	5*s* ^4^P_3/2_ — 7*p*′ ${\;}^{2}D_{3/2}^{{}^{\circ}}$
3589.818	100	27848.62	5*s* ^4^P_3/2_ — 7*p*′ ${\;}^{2}D_{3/2}^{{}^{\circ}}$
3599.962	100	27770.15	5*s* ^4^P_3/2_ — 9*p* ${\;}^{4}D_{5/2}^{{}^{\circ}}$
3606.518	20	27719.67	5*s* ^4^P_3/2_ — 9*p* ${\;}^{4}D_{5/2}^{{}^{\circ}}$
3608.864	7	27701.65	5*s* ^4^P_3/2_ — 9*p* ${\;}^{4}P_{1/2}^{{}^{\circ}}$
3644.165 3644.198	$\begin{array}{l} \;\\ \; \end{array}\}\begin{array}{c} 15\\ 10 \end{array}$	27433.31 27433.06	$\begin{array}{l} \;\\ \; \end{array}\}\;5s{\;}^{4}P_{5/2}—5f{\left[2\right]}_{5/2}^{{}^{\circ}}$
3646.605 3646.626	$\begin{array}{l} \;\\ \; \end{array}\}\begin{array}{c} 100\\ 80 \end{array}$	27414.96 27414.80	$\begin{array}{l} \;\\ \; \end{array}\}\;5s{\;}^{4}P_{5/2}—5f{\left[3\right]}_{7/2,5/2}^{{}^{\circ}}$
3658.486	*2h*	27325.93	5*s* ^4^P_3/2_ — 6*f* ${\left[1\right]}_{3/2}^{{}^{\circ}}$
3659.970	20	27314.85	5*s* ^4^P_3/2_ — 8*p* ${\left[2\right]}_{5/2}^{{}^{\circ}}$
3735.800	500	26760.42	5*s* ^4^P_3/2_ — 8*p* ${\;}^{4}D_{3/2}^{{}^{\circ}}$
3745.419	50	26691.70	5*s* ^4^P_3/2_ — 8*p* ${\;}^{4}P_{1/2}^{{}^{\circ}}$
3751.287	10	26649.94	5*s* ^4^P_3/2_ — 8*p* ${\;}^{4}D_{5/2}^{{}^{\circ}}$
3753.351	5	26635.29	5*s* ^4^P_3/2_ — 4*f*′ ${\left[3\right]}_{5/2}^{{}^{\circ}}$
3756.032	30	26616.28	5*s*′ ^4^P_1/2_ — 7*p*″ ${\;}^{2}P_{3/2}^{{}^{\circ}}$
3757.265	5	26607.54	5*s*′ ^4^P_1/2_ — 7*p*″ ${\;}^{2}P_{1/2}^{{}^{\circ}}$
3761.746	2	26575.85	5*s* ^4^P_3/2_ — 4*f*′ ${\left[2\right]}_{5/2}^{{}^{\circ}}$
3761.847	1	26575.14	5*s* ^4^P_3/2_ — 4*f*′ ${\left[2\right]}_{3/2}^{{}^{\circ}}$
3770.88	150*c*	26511.48	5*s* ^4^P_5/2_ — 6*p*″ ${\;}^{2}P_{3/2}^{{}^{\circ}}$
3794.03	900*c*	26349.72	5*s* ^4^P_5/2_ — 7*p* ${\;}^{4}D_{3/2}^{{}^{\circ}}$
3798.805	5*w*	26316.60	5*s*′ ^2^P_3/2_ — 7*p*″ ${\;}^{2}P_{3/2}^{{}^{\circ}}$
3810.95	1*h*	26232.73	5*s* ^4^P_5/2_ — 7*p* ${\;}^{4}D_{5/2}^{{}^{\circ}}$
3815.650	1200	26200.42	5*s* ^4^P_5/2_ — 7*p* ${\;}^{4}D_{7/2}^{{}^{\circ}}$
3828.505	700	26112.45	5*s* ^4^P_5/2_ — 7*p* ${\;}^{4}P_{3/2}^{{}^{\circ}}$
3829.75	200*c*	26103.96	5*s* ^4^P_3/2_ — 7*p* ${\;}^{4}P_{3/2}^{{}^{\circ}}$
3844.035	100	26006.95	5*s*′ ^4^P_1/2_ — 7*p*′ ${\;}^{4}D_{1/2}^{{}^{\circ}}$
3847.37	5*h*	25984.41.	5*s* ^4^P_3/2_ — 5*f* ${\left[1\right]}_{3/2}^{{}^{\circ}}$
3850.619	10	25962.49	5*s* ^4^P_3/2_ — 5*f* ${\left[2\right]}_{5/2}^{{}^{\circ}}$
3850.740	1	25961.67	5*s* ^4^P_3/2_ — 5*f* ${\left[2\right]}_{3/2}^{{}^{\circ}}$
3851.654	3	25955.51	5*s*′ ^4^P_1/2_ — 7*p*′ ${\;}^{4}S_{3/2}^{{}^{\circ}}$
3854.702	90	25934.99	5*s*′ ^4^P_1/2_ — 7*p*′ ${\;}^{2}D_{3/2}^{{}^{\circ}}$
3896.653	200	25655.78	5*s*′ ^2^P_3/2_ — 7*p*′ ${\;}^{4}S_{3/2}^{{}^{\circ}}$
3899.767	25	25635.29	5*s*′ ^2^P_3/2_ — 7*p*′ ${\;}^{2}D_{3/2}^{{}^{\circ}}$
3905.823 3905.858	$\begin{array}{l} \;\\ \; \end{array}\}\begin{array}{c} 25w\\ 20 \end{array}$	25595.55 25595.32	$\begin{array}{l} \;\\ \; \end{array}\}\;5s{\;}^{4}P_{5/2}—6p\prime \;S_{3/2}^{{}^{\circ}}$
3909.385	175	25572.23	5*s*′ ^2^P_3/2_ — 7*p*′ ${\;}^{2}D_{5/2}^{{}^{\circ}}$
3913.560	12	25544.95	5*s*′ ^2^P_3/2_ — 7*p*′ ${\;}^{2}S_{1/2}^{{}^{\circ}}$
3917.775 3917.819	$\begin{array}{l} \;\\ \;\\ \; \end{array}\}\begin{array}{c} \begin{array}{l} 200\\ \; \end{array}\\ 150 \end{array}$	25517.47 25517.18	$\begin{array}{l} \;\\ \;\\ \; \end{array}\}\;5s{\;}^{4}P_{5/2}—6p\prime {\;}^{2}D_{3/2}^{{}^{\circ}}$
3929.196	5	25443.30	5*s*′ ^2^P_3/2_ — 9*p* ${\;}^{4}D_{5/2}^{{}^{\circ}}$
3934.068 3934.084 3934.119	$\begin{array}{l} \;\\ \;\\ \; \end{array}\}\begin{array}{c} \begin{array}{l} 60\\ 80 \end{array}\\ 100 \end{array}$	25411.79 25411.68 25411.46	$\begin{array}{l} \;\\ \;\\ \; \end{array}\}\;5s{\;}^{4}P_{5/2}—6p\prime {\;}^{2}D_{5/2}^{{}^{\circ}}$
3942.026	2*h*	25360.49	
3943.776	2	25349.23	5*s*′ ^4^P_1/2_ — 6*f* ${\left[1\right]}_{3/2}^{{}^{\circ}}$
3943.835	8	25348.86	5*s*′ ^4^P_1/2_ — 6*f* ${\left[1\right]}_{1/2}^{{}^{\circ}}$
3945.565	4	25337.74	5*s*′ ^4^P_1/2_ — 6*f* ${\left[2\right]}_{3/2}^{{}^{\circ}}$
3969.416	1	25185.50	
3960.434	1	25185.38	
3973.967	5	25156.66	
3991.363	300	25047.01	5*s* ^4^P_5/2_ — (^1^D_2_) 5*p* ${\;}^{2}D_{5/2}^{{}^{\circ}}$
3992.363	1500	25040.74	5*s* ^4^P_3/2_ — 6*p*″ ${\;}^{2}P_{3/2}^{{}^{\circ}}$
3993.177	5	25035.64	
3998.492	35	25002.36	5*s* ^4^P_5/2_ — (^1^D_2_) 5*p* ${\;}^{2}D_{3/2}^{{}^{\circ}}$
3099.070	200	24998.75	5*s* ^4^P_3/2_ — 6*p*″ ${\;}^{2}P_{1/2}^{{}^{\circ}}$
a 4000.599	2*w*	24989.19	5*s* ^4^P_5/2_ — 4*f* ${\left[1\right]}_{3/2}^{{}^{\circ}}$
4009.296 4009.325	$\begin{array}{l} \;\\ \; \end{array}\}\begin{array}{c} 2w\\ 2 \end{array}$	2493499 24934.81	$\begin{array}{l} \;\\ \; \end{array}\}\;5s{\;}^{4}P_{5/2}—4f\;{\left[2\right]}_{5/2}^{{}^{\circ}}$
4012.548 4012.580	$\begin{array}{l} \;\\ \; \end{array}\}\begin{array}{c} 60s\\ 50 \end{array}$	24914.78 24914.58	$\begin{array}{l} \;\\ \; \end{array}\}\;5s{\;}^{4}P_{5/2}—4f\;{\left[3\right]}_{7/2}^{{}^{\circ}}$
4016.453	1*h*	24890.55	
4018.316	200	24879.01	5*s* ^4^P_3/2_ — 7*p* ${\;}^{4}D_{3/2}^{{}^{\circ}}$
4021.772	250	24857.64	5*s* ^4^P_3/2_ — 7*p* ${\;}^{4}P_{1/2}^{{}^{\circ}}$
4033.762	1*h*	24783.75	5*s*′ ^4^P_1/2_ — 8*p* ${\;}^{4}D_{3/2}^{{}^{\circ}}$
4037.324	500	24761.89	5*s* ^4^P_3/2_ — 7*p* ${\;}^{4}D_{5/2}^{{}^{\circ}}$
4056.404 4056.425	$\begin{array}{l} \;\\ \; \end{array}\}\begin{array}{c} 30s\\ 25 \end{array}$	24645.42 24645.29	$\begin{array}{l} \;\\ \;\\ \; \end{array}\}\;5s\prime {\;}^{4}P_{1/2}—8p{\;}^{4}P_{3/2}^{{}^{\circ}}$
4057.020	30	24641.67	5*s* ^4^P_3/2_ — 7*p* ${\;}^{4}P_{5/2}^{{}^{\circ}}$
4058.406	75	24633.26	5*s* ^4^P_3/2_ — 7*p* ${\;}^{4}P_{3/2}^{{}^{\circ}}$
4064.150	4	24598.44	5*s*′ ^4^P_1/2_ — 4*f*′ ${\left[2\right]}_{3/2}^{{}^{\circ}}$
4075.503	50	24529.92	5*s*″ ^2^P_1/2_ — 7*p*″ ${\;}^{2}P_{3/2}^{{}^{\circ}}$
4076.956	75	24521.18	5*s*′ ^2^P_1/2_ — 7*p*″ ${\;}^{2}P_{1/2}^{{}^{\circ}}$
4083.149	50	24483.99	5*s*′ ^2^P_3/2_ — 8*p* ${\;}^{4}D_{3/2}^{{}^{\circ}}$
4094.643	35	24415.26	5*s*′ ^2^P_3/2_ — 8*p* ${\;}^{4}P_{1/2}^{{}^{\circ}}$
4106.352	25	24345.65	5*s*′ ^2^P_3/2_ — 8*p* ${\;}^{4}P_{3/2}^{{}^{\circ}}$
4113.132	4	24305.52	5*s*′ ^2^P_3/2_ — 8*p* ${\;}^{4}P_{5/2}^{{}^{\circ}}$
4114.165	25	24299.41	5*s*′ ^2^P_3/2_ — 4*f*′ ${\left[2\right]}_{5/2}^{{}^{\circ}}$
4124.233	75	24240.09	5*s* ^4^P_3/2_ — 6*p*′ ${\;}^{4}D_{1/2}^{{}^{\circ}}$
4143.974	600	24124.62	5*s* ^4^P_3/2_ — 6*p*′ ${\;}^{4}S_{3/2}^{{}^{\circ}}$
4157.425	250	24046.57	5*s* ^4^P_3/2_ — 6*p*′ ${\;}^{2}D_{3/2}^{{}^{\circ}}$
4164.158	4	24007.69	5*s*′ ^4^P_1/2_ — 5*f* ${\left[1\right]}_{3/2}^{{}^{\circ}}$
4164.270	15	24007.04	5*s*′ ^4^P_1/2_ — 5*f* ${\left[1\right]}_{1/2}^{{}^{\circ}}$
4168.115	10	23984.90	5*s*′ ^4^P_1/2_ — 5*f* ${\left[2\right]}_{3/2}^{{}^{\circ}}$
4175.786	50	23940.84	5*s* ^4^P_3/2_ — 6*p*′ ${\;}^{2}D_{5/2}^{{}^{\circ}}$
4179.322	100	23920.58	5*s*″ ^2^P_1/2_ — 7*p*′ ${\;}^{4}D_{1/2}^{{}^{\circ}}$
4191.935	50	23848.61	5*s*″ ^2^P_1/2_ — 7*p*′ ${\;}^{2}D_{3/2}^{{}^{\circ}}$
4196.468	50	23822.85	5*s* ^4^P_5/2_ — (^1^D_2_) 5*p* ${\;}^{3}P_{3/2}^{{}^{\circ}}$
4199.473	35	23805.80	5*s* ^4^P_3/2_ — 6*p*′ ${\;}^{2}S_{1/2}^{{}^{\circ}}$
4202.487	450	23788.73	5*s* ^4^P_5/2_ — (^1^D_2_) 5*p* ${\;}^{2}F_{7/2}^{{}^{\circ}}$
4220.701	5*h*	23686.08	5*s*′ ^2^P_3/2_ — 5*f* ${\left[2\right]}_{5/2}^{{}^{\circ}}$
4220.859	2	23685.19	5*s*′ ^2^P_3/2_ — 5*f* ${\left[2\right]}_{3/2}^{{}^{\circ}}$
4231.643	4	23624.83	5*s* ^4^P_5/2_ — (^1^D_2_) 5*p* ${\;}^{2}F_{5/2}^{{}^{\circ}}$
4240.366	40	23576.23	5*s* ^4^P_3/2_ — (^1^D_2_) 5*p* ${\;}^{2}D_{5/2}^{{}^{\circ}}$
4248.414	50	23531.57	5*s* ^4^P_3/2_ — (^1^D_2_) 5*p* ${\;}^{2}D_{3/2}^{{}^{\circ}}$
4250.818	40	23518.26	5*s* ^4^P_3/2_ — 4*f* ${\left[1\right]}_{3/2}^{{}^{\circ}}$
4250.924	15	23517.68	5*s* ^4^P_3/2_ — 4*f* ${\left[1\right]}_{1/2}^{{}^{\circ}}$
4260.625	40	23464.13	5*s* ^4^P_3/2_ — 4*f* ${\left[2\right]}_{5/2}^{{}^{\circ}}$
4261.964	3	23456.76	5*s* ^4^P_3/2_ — 4*f* ${\left[2\right]}_{3/2}^{{}^{\circ}}$
4334.54	20	23064.01.	5*s*′ ^4^P_1/2_ — 6*p*″ ${\;}^{2}P_{3/2}^{{}^{\circ}}$
4342.45	2	23022.00	5*s*′ ^4^P_1/2_ — 6*p*″ ${\;}^{2}P_{1/2}^{{}^{\circ}}$
4365.14	2000	22902.34	5*s*′ ^4^P_1/2_ — 7*p* ${\;}^{4}D_{3/2}^{{}^{\circ}}$
4369.22	75	22880.95	5*s*′ ^4^P_1/2_ — 7*p* ${\;}^{4}P_{1/2}^{{}^{\circ}}$
4391.60	800	22764.35	5*s*′ ^2^P_3/2_ — 6*p*″ ${\;}^{2}P_{3/2}^{{}^{\circ}}$
4399.73	450	22722.28	5*s*′ ^2^P_3/2_ — 6*p*″ ${\;}^{2}P_{1/2}^{{}^{\circ}}$
4404.57	10	22697.32	5*s*″ ^2^P_3/2_ — 8*p* ${\;}^{4}D_{3/2}^{{}^{\circ}}$
4412.49	100	22656.58	5*s*′ ^4^P_1/2_ — 7*p* ${\;}^{4}P_{3/2}^{{}^{\circ}}$
4423.0:3	200	22602.59	5*s*′ ^2^P_3/2_ — 7*p* ${\;}^{4}D_{3/2}^{{}^{\circ}}$
4425.14	1500	22591.81	5*s* ^4^P_3/2_ — (^1^D_2_) 5*p* ${\;}^{2}P_{1/2}^{{}^{\circ}}$
4427.20	40	22581.30	5*s*′ ^2^P_3/2_ — 7*p* ${\;}^{4}P_{1/2}^{{}^{\circ}}$
4431.59	1	22558.9:3	5*s*″ ^2^P_1/2_ — 8*p* ${\;}^{4}P_{3/2}^{{}^{\circ}}$
4441.74	10000	22507.38	5*s* ^4^P_5/2_ — 7*p* ${\;}^{4}D_{3/2}^{{}^{\circ}}$
b4446.08	15	22485.41	5*s*′ ^2^P_3/2_ — 7*p* ${\;}^{4}D_{5/2}^{{}^{\circ}}$
4466.21	200	22384.07	5*s* ^4^P_5/2_ — 6*p* ${\;}^{4}D_{5/2}^{{}^{\circ}}$
4469.98	150	22365.19	5*s*′ ^2^P_3/2_ — 7*p* ${\;}^{4}P_{5/2}^{{}^{\circ}}$
4471.67	80	22356.74	5*s*′ ^2^P_3/2_ — 7*p* ${\;}^{4}P_{3/2}^{{}^{\circ}}$
4472.61	10000	22352.04	5*s* ^4^P_3/2_ — (^1^D_2_) 5*p* ${\;}^{2}P_{3\;2}^{{}^{\circ}}$
4477.72	20000	22326.53	5*s* ^4^P_5/2_ — 6*p* ${\;}^{4}D_{7/2}^{{}^{\circ}}$
4490.42	1000	22263.39	5*s*′ ^4^P_1/2_ — 6*p*′ ${\;}^{4}D_{1/2}^{{}^{\circ}}$
4513.44	3000	22149.84	5*s* ^4^P_5/2_ — 6*p* ${\;}^{4}P_{3/2}^{{}^{\circ}}$
4513.82	150	22147.97	5*s*′ ^4^P_1/2_ — 6*p′* ${\;}^{4}S_{3/2}^{{}^{\circ}}$
4525.59	15000	22090.37	5*s* ^4^P_5/2_ — 6*p* ${\;}^{4}P_{5/2}^{{}^{\circ}}$
4529.79	600	22069.89	5*s*′ ^4^P_1/2_ — 6*p*′ ${\;}^{2}D_{3/2}^{{}^{\circ}}$
4575.74	3000	21848.27	5*s*′ ^2^P_3/2_ — 6*p′* ${\;}^{4}S_{3/2}^{{}^{\circ}}$
4592.16	200	21770.15	5*s*′ ^2^P_3/2_ — 6*p′* ${\;}^{2}D_{3/2}^{{}^{\circ}}$
4614.58	2500	21664.38	5*s*′ ^2^P_3/2_ — 6*p′* ${\;}^{2}D_{5/2}^{{}^{\circ}}$
4640.92	40	21541.42	5*s*′ ^4^P_1/2_ — 4*f* ${\left[1\right]}_{3/2}^{{}^{\circ}}$
4641.02	70	21540.96	5*s*′ ^4^P_1/2_ — 4*f* ${\left[1\right]}_{1/2}^{{}^{\circ}}$
4643.52	900	21529.36	5*s*′ ^2^P_3/2_ — 6*p′* ${\;}^{2}S_{1/2}^{{}^{\circ}}$
b 4654.18	25	21480.05	5*s*′ ^4^P_1/2_ — 4*f* ${\left[2\right]}_{3/2}^{{}^{\circ}}$
4693.56	300	21299.83	5*s*′ ^2^P_3/2_ — (^1^D_2_) 5*p* ${\;}^{2}D_{5/2}^{{}^{\circ}}$
4703.42	50	21255.18	5*s*′ ^2^P_3/2_ — (^1^D_2_) 5*p* ${\;}^{2}D_{3/2}^{{}^{\circ}}$
4706.36	12*h*	21241.90	5*s*′ ^2^P_3/2_ — 4*f* ${\left[1\right]}_{3/2}^{{}^{\circ}}$
4720.03	100	21180.38	5*s*′ ^2^P_3/2_ — 4*f* ${\left[2\right]}_{3/2}^{{}^{\circ}}$
4722.97	9	21167.20	5*s*′ ^2^P_3/2_ — 4*f* ${\left[3\right]}_{5/2}^{{}^{\circ}}$
4752.28	2500	21036.65	5*s* ^4^P_3/2_ — 6*p* ${\;}^{4}D_{3/2}^{{}^{\circ}}$
4765.63	250	20977.72	5*s*″ ^2^P_1/2_ — 6*p*″ ${\;}^{2}P_{3/2}^{{}^{\circ}}$
4775.20	750	20935.68	5*s*″ ^2^P_1/2_ — 6*p*″ ${\;}^{2}P_{1/2}^{{}^{\circ}}$
4780.31	4000	20913.30	5*s* ^4^P_3/2_ — 6*p* ${\;}^{4}D_{5/2}^{{}^{\circ}}$
4785.19	1600	20891.97	5*s* ^4^P_3/2_ — 6*p* ${\;}^{4}P_{1/2}^{{}^{\circ}}$
4802.67	250	20815.93	5*s*″ ^2^P_1/2_ — 7*p* ${\;}^{4}D_{3/2}^{{}^{\circ}}$
4807.61	350	20794.54	5*s*″ ^2^P_1/2_ — 7*p* ${\;}^{4}P_{1/2}^{{}^{\circ}}$
4834.46	500	20679.06	5*s* ^4^P_3/2_ — 6*p* ${\;}^{4}P_{3/2}^{{}^{\circ}}$
4848.39	25	20619.64	5*s* ^4^P_3/2_ — 6*p* ${\;}^{4}P_{5/2}^{{}^{\circ}}$
4860.04	250	20570.22	5*s*″ ^2^P_1/2_ — 7*p* ${\;}^{4}P_{3/2}^{{}^{\circ}}$
4906.49	10	20375.48	5*s*′ ^4^P_1/2_ — (^1^D_2_) 5*p* ${\;}^{2}P_{3/2}^{{}^{\circ}}$
4920.98	300	20315.48	5*s*′ ^2^P_3/2_ — (^1^D_2_) 5*p* ${\;}^{2}P_{1/2}^{{}^{\circ}}$
4921.80	10*w*	20312.10	
4954.73	300	20177.10	5*s*″ ^2^P_1/2_ — 6*p*′ ${\;}^{2}D_{1/2}^{{}^{\circ}}$
4979.76	4000	20075.69	5*s*′ ^2^P_3/2_ — (^1^D_2_) 5*p* ${\;}^{2}P_{3/2}^{{}^{\circ}}$
4983.25	75	20061.63	5*s*″ ^2^P_1/2_ — 6*p*′ ${\;}^{4}S_{3/2}^{{}^{\circ}}$
5002.72	500	19983.55	5*s*″ ^2^P_1/2_ — 6*p*′ ${\;}^{2}D_{3/2}^{{}^{\circ}}$
5029.37	200	19877.66	5*s*′ ^2^P_3/2_ — (^1^D_2_) 5*p* ${\;}^{2}F_{5/2}^{{}^{\circ}}$
5063.74	40	19742.75	5*s*″ ^2^P_1/2_ — 6*p*′ ${\;}^{2}S_{1/2}^{{}^{\circ}}$
5092.60	4	19630.86	
5138.55	10	19455.32	5*s*″ ^2^P_1/2_ — 4*f* ${\left[1\right]}_{3/2}^{{}^{\circ}}$
5139.47	10*w*	19451.84	
5148.78	10	19416.67	5*p* ${\;}^{4}P_{5/2}^{{}^{\circ}}$ — 6*d*″ ^2^D_5/2_
5150.47	6*h*	19410.30	5*p* ${\;}^{4}P_{5/2}^{{}^{\circ}}$ — 6*d*″ ^2^D_3/2_
5220.71	8	19149.15	5*p* ${\;}^{4}P_{5/2}^{{}^{\circ}}$ — 10*d* ^4^D_3/2_
5222.32	12	19143.25	5*p* ${\;}^{4}P_{5/2}^{{}^{\circ}}$ — 10*d* ^4^D_5/2_
5226.91	20	19126.44	5*p* ${\;}^{4}P_{5/2}^{{}^{\circ}}$— 10*d* ^4^D_7/2_
5211.43	5	19073.45	5*p* ${\;}^{4}P_{3/2}^{{}^{\circ}}$ — 6*d*″ ^2^D_3/2_
5245.12	350	19060.04	5*s′* ^4^P_1/2_ — 6*p* ${\;}^{4}D_{3/2}^{{}^{\circ}}$
5261.80	5	18999.62	5*p* ${\;}^{4}P_{5/2}^{{}^{\circ}}$ — (^1^D_2_) 6*d* ^2^F_5/2_
5285.23	50	18915.39	5*s′* ^4^P_1/2_ — 6*p* ${\;}^{4}P_{1/2}^{{}^{\circ}}$
5297.32	15	18872.22	5*p* ${\;}^{4}P_{5/2}^{{}^{\circ}}$ — 11*s* ^4^P_5/2_
5314.18	10	13812.34	5*p* ${\;}^{4}P_{3/2}^{{}^{\circ}}$ — 10*d* ^4^D_3/2_
5315.85	8	18806.44	5*p* ${\;}^{4}P_{3/2}^{{}^{\circ}}$ — 10*d* ^4^D_5/2_
5317.25	20	18801.48	5*p* ${\;}^{4}P_{5/2}^{{}^{\circ}}$ — 6*d*′ ^4^P_5/2_
5318.83	7	13795.90	5*p* ${\;}^{4}D_{1/2}^{{}^{\circ}}$ — 12*d* ^4^F_9/2_
5323.20	12	18730.47	5*p* ${\;}^{4}P_{5/2}^{{}^{\circ}}$ — 6*d*′ ^4^P_3/2_
5328.92	200	18760.31	5*s′* ^2^P_3/2_ — 6*p* ${\;}^{4}D_{3/2}^{{}^{\circ}}$
5329.30	12	18758.97	5*p* ${\;}^{4}P_{5/2}^{{}^{\circ}}$ — 9*d* ^4^F_7/2_
5337.10	40	18731.56	5*p* ${\;}^{4}P_{5/2}^{{}^{\circ}}$ — 9*d* ^4^D_7/2_
5345.42	600	18702.40	5*s′* ^4^P_1/2_ — 6*p* ${\;}^{4}P_{3/2}^{{}^{\circ}}$
5348.29	10	18692.37	5*p* ${\;}^{4}P_{3/2}^{{}^{\circ}}$ — (^1^D_2_) 4*d* ^2^S_1/2_
5354.67	120	18670.09	5*p* ${\;}^{4}P_{5/2}^{{}^{\circ}}$ — 6*d*′ ^2^F_5/2_
5356.74	12*w*	18662.88	5*p* ${\;}^{4}P_{3/2}^{{}^{\circ}}$ — (^1^D_2_) 4*d* ^2^F_5/2_
5364.19	300	18636.96	5*s′* ^2^P_3/2_ — 6*p* ${\;}^{4}D_{5/2}^{{}^{\circ}}$
5370.34	300	18615.62	5*s′* ^2^P_3/2_ — 6*p* ${\;}^{4}P_{1/2}^{{}^{\circ}}$
5379.13	15	18585.20	5*p* ${\;}^{4}D_{7/2}^{{}^{\circ}}$ — 11*d* ^2^F_9/2_
5382.96	120	18571.98	5*p* ${\;}^{4}P_{5/2}^{{}^{\circ}}$ — 6*d*′ ^2^F_7/2_
5384.27	8	18567.46	5*p* ${\;}^{4}D_{7/2}^{{}^{\circ}}$ — 6*d*″ ^2^D_5/2_
5393.58	7	18535.41	5*p* ${\;}^{4}P_{3/2}^{{}^{\circ}}$ — 11*s* ^2^P_5/2_
5395.48 5395.55	$\begin{array}{l} \;\\ \; \end{array}\}\begin{array}{c} 1200\\ 900 \end{array}$	18528.88 18528.64	$\begin{array}{l} \;\\ \; \end{array}\}\;5s\prime \prime {\;}^{2}P_{1/2}—\left({\;}^{1}D_{2}\right)5p{\;}^{2}P_{1/2}^{{}^{\circ}}$
5414.26	4	18464.61	5*p* ${\;}^{4}P_{3/2}^{{}^{\circ}}$ — 6*d*′ ^4^P_5/2_
5420.43	13	18443.59	5*p* ${\;}^{4}P_{3/2}^{{}^{\circ}}$ — 6*d*′ ^4^P_3/2_
5420.80	18	1,442.33	5*p* ${\;}^{4}P_{3/2}^{{}^{\circ}}$ — 9*d* ^4^P_1/2_
5424.61	8*h*	18429.38	5*p* ${\;}^{4}D_{5/2}^{{}^{\circ}}$ — 11*d* ^4^F_7/2_
5432.47	60	18402.72	5*s′* ^2^P_3/2_ — 6*p* ${\;}^{4}P_{3/2}^{{}^{\circ}}$
5434.22	7	18390.79	5*p* ${\;}^{4}P_{3/2}^{{}^{\circ}}$ — 9*d* ^4^D_5/2_
5450.09	550	18343.22	5*s′* ^2^P_3/2_ — 6p ${\;}^{4}P_{5/2}^{{}^{\circ}}$
5453.03	90	18333.33	5*p* ${\;}^{4}P_{3/2}^{{}^{\circ}}$ — 6*d*′ ^2^F_5/2_
5455.16	60	18326.17	5*p* ${\;}^{4}P_{3/2}^{{}^{\circ}}$ — 9*d* ^4^D_3/2_
5455.96	70	18323.49	5*p* ${\;}^{4}P_{5/2}^{{}^{\circ}}$ — 10*s* ^4^P_5/2_
5463.72	80	18297.46	5*p* ${\;}^{4}D_{7/2}^{{}^{\circ}}$ — 10*d* ^4^F_9/2_
5466.22	1200	18289.10	5*s*″ ^2^P_1/2_ — (^1^D_2_) 5*p* ${\;}^{2}P_{3/2}^{{}^{\circ}}$
5469.76	15	18277.26	5*p* ${\;}^{4}D_{7/2}^{{}^{\circ}}$ — 10*d* ^4^D_7/2_
5470.90	5	18273.45	5*p* ${\;}^{4}P_{3/2}^{{}^{\circ}}$ — 9*d* ^4^D_1/2_
5504.46	35*h*	18162.04	5*p* ${\;}^{4}D_{5/2}^{{}^{\circ}}$ — 10*d* ^4^F_7/2_
5520.86	20	18108.09	5*p* ${\;}^{4}P_{3/2}^{{}^{\circ}}$ — 6*d*′ ^2^P_3/2_
5522.53	90	18102.61	5*p* ${\;}^{4}P_{5/2}^{{}^{\circ}}$ — 8*d* ^4^D_3/2_
5529.01	175	18081.40	5*p* ${\;}^{4}P_{5/2}^{{}^{\circ}}$ — 8*d* ^4^D_5/2_
5532.22	100	18070.91	5*p* ${\;}^{4}P_{5/2}^{{}^{\circ}}$ — 8*d* ^4^F_5/2_
5536.37	300	18057.36	5*p* ${\;}^{4}P_{5/2}^{{}^{\circ}}$ — 8*d* ^4^D_7/2_
5544.72	6	18030.17	5*p* ${\;}^{4}P_{3/2}^{{}^{\circ}}$ — 10*s* ^4^P_3/2_
5546.90	20	18023.08	5*p* ${\;}^{4}D_{7/2}^{{}^{\circ}}$ — 11*s* ^4^P_5/2_
5558.13	40	17986.67	5*p* ${\;}^{4}P_{3/2}^{{}^{\circ}}$ — 10*s* ^4^P_5/2_
5558.40	10	17985.79	5*p* ${\;}^{4}D_{5/2}^{{}^{\circ}}$ — (^1^D_2_) 4*d* ^2^F_7/2_
5588.17	160	17889.98	5*p* ${\;}^{4}D_{7/2}^{{}^{\circ}}$ — 9*d* ^4^F_9/2_
5590.56	50	17882.33	5*p* ${\;}^{4}D_{7/2}^{{}^{\circ}}$ — 9*d* ^4^D_7/2_
5597.26	15	17860.93	5*p* ${\;}^{4}D_{5/2}^{{}^{\circ}}$ — 11*s* ^4^P_3/2_
5602.41	8*h*	17844.51	$\left\{ \begin{array}{l} 5p{\;}^{4}P_{3/2}^{{}^{\circ}}—8d{\;}^{4}F_{3/2}\\ 5p{\;}^{4}P_{5/2}^{{}^{\circ}}—\left({\;}^{1}D_{2}\right)4d{\;}^{2}D_{3/2} \end{array}\right.$
5604.96	4	17836.39	5*p* ${\;}^{4}P_{1/2}^{{}^{\circ}}$ — (^1^D_2_) 4*d* ^2^P_1/2_
5609.83	10*h*	17820.91	5*p* ${\;}^{4}D_{7/2}^{{}^{\circ}}$ — 6*d*′ ^2^F_5/2_
5611.62	125	17815.22	5*p* ${\;}^{4}P_{3/2}^{{}^{\circ}}$ — 8*d* ^4^P_1/2_
5627.24	200	17765.77	5*p* ${\;}^{4}P_{3/2}^{{}^{\circ}}$ — 8*d* ^4^D_3/2_
5633.97	140	17744.55	5*p* ${\;}^{4}P_{3/2}^{{}^{\circ}}$ — 8*d* ^4^D_5/2_
5637.27	130	17734.16	5*p* ${\;}^{4}D_{5/2}^{{}^{\circ}}$ — 9*d* ^4^F_7/2_
5640.86	3.5	17722.87	5*p* ${\;}^{4}D_{7/2}^{{}^{\circ}}$ — 6*d*′ ^2^F_7/2_
5645.31	30	17708.90	5*p* ${\;}^{4}D_{5/2}^{{}^{\circ}}$ — 9*d* ^4^D_5/2_
5645.97	45	17706.83	5*p* ${\;}^{4}D_{5/2}^{{}^{\circ}}$ — 9*d* ^4^D_7/2_
5667.75	9	17638.79	5*p* ${\;}^{4}P_{1/2}^{{}^{\circ}}$ — 6*d*′ ^4^P_3/2_
5667.92	8	17638.26	5*p* ${\;}^{4}D_{5/2}^{{}^{\circ}}$ — 9*d* ^4^D_3/2_
5685.77	15*W*	17582.89	(^1^D_2_)5*s* ^2^D_3/2_ — 7*p*″ ${\;}^{2}P_{1/2}^{{}^{\circ}}$
5697.29	18	17547.34	5*p* ${\;}^{4}D_{5/2}^{{}^{\circ}}$ — 6*d*′ ^2^F_7/2_
5705.74	85	17521.35	5*p* ${\;}^{4}P_{1/2}^{{}^{\circ}}$ — 9*d* ^4^D_3/2_
5709.44	9	17509.99	5*p* ${\;}^{4}P_{5/2}^{{}^{\circ}}$ — 9*s* ^4^P_3/2_
5716.26	225	17489.10	5*p* ${\;}^{4}P_{5/2}^{{}^{\circ}}$ — 9*s* ^4^P_5/2_
5721.11	100	17474.28	5*p* ${\;}^{4}D_{7/2}^{{}^{\circ}}$ — 10*s* ^4^P_5/2_
5722.97	40*h*	17468.60	5*p* ${\;}^{4}P_{1/2}^{{}^{\circ}}$ — 9*d* ^4^D_1/2_
5764.66	45	17342.27	5*p* ${\;}^{4}D_{5/2}^{{}^{\circ}}$ — 10*s* ^4^P_3/2_
5771.30	5	17322.31	5*p* ${\;}^{4}D_{7/2}^{{}^{\circ}}$ — 8*d* ^4^F_7/2_
5777.69	10	17303.16	5*p* ${\;}^{4}P_{1/2}^{{}^{\circ}}$ — 6*d*′ ^2^P_3/2_
5779.97	4	17296.33	
5783.32	500	17286.31	5*p* ${\;}^{4}D_{7/2}^{{}^{\circ}}$ — 8*d* ^4^F_9/2_
5784.02	30	17284.22	5*p* ${\;}^{4}P_{5/2}^{{}^{\circ}}$ — 7*d* ^4^F_5/2_
5793.98	100	17254.51	5*p* ${\;}^{4}P_{5/2}^{{}^{\circ}}$ — 7*d* ^4^F_3/2_
5801.50	40	17232.14	5*p* ${\;}^{4}D_{7/2}^{{}^{\circ}}$ — 8*d* ^4^D_5/2_
5803.82	30	17225.26	5*p* ${\;}^{4}P_{1/2}^{{}^{\circ}}$ — 10*s* ^4^P_3/2_
5805.04	30	17221.64	5*p* ${\;}^{4}D_{7/2}^{{}^{\circ}}$ — 8*d* ^4^F_5/2_
5809.59	250	17208.15	5*p* ${\;}^{4}D_{7/2}^{{}^{\circ}}$ — 8*d* ^4^F_7/2_
5819.56	80	17178.67	5*p* ${\;}^{4}P_{1/2}^{{}^{\circ}}$ — 6*d*′ ^2^P_1/2_
5821.45	12	17173.09	5*p* ${\;}^{4}P_{3/2}^{{}^{\circ}}$ — 9*s* ^4^P_3/2_
5826.07	5	17159.47	
5827.08	10	17156.50	5*p* ${\;}^{4}D_{5/2}^{{}^{\circ}}$ — 8*d* ^4^F_3/2_
5828.51	150	17152.29	5*p* ${\;}^{4}P_{3/2}^{{}^{\circ}}$ — 9*s* ^4^P_5/2_
5828.89	7	17151.17	
5830.39	400	17146.76	5*p* ${\;}^{4}D_{5/2}^{{}^{\circ}}$ — 8*d* ^4^F_7/2_
5833.39	900	17137.94	5*p* ${\;}^{4}P_{5/2}^{{}^{\circ}}$ — 7*d* ^4^D_5/2_
5836.84	150	17127.81	5*p* ${\;}^{4}P_{5/2}^{{}^{\circ}}$ — 7*d* ^4^D_3/2_
5852.08	1800	17083.21	5*p* ${\;}^{4}P_{5/2}^{{}^{\circ}}$ — 7*d* ^4^D_7/2_
5861.20	120	17056.62	5*p* ${\;}^{4}D_{5/2}^{{}^{\circ}}$ — 8*d* ^4^D_5/2_
5864.82	90	17046.10	5*p* ${\;}^{4}D_{5/2}^{{}^{\circ}}$ — 8*d* ^4^F_5/2_
5867.05	60	17039.62	5*p* ${\;}^{4}P_{1/2}^{{}^{\circ}}$ — 8*a* ^4^F_3/2_
5869.47	18	17032.59	5*p* ${\;}^{4}D_{5/2}^{{}^{\circ}}$ — 8*d* ^4^D_7/2_
5877.15	50*w*	17010.34	5*p* ${\;}^{4}P_{1/2}^{{}^{\circ}}$ — 8*d* ^4^P_1/2_
5886.87	60*cw*	16982.25	(^1^D_2_)5*s* ^2^D_3/2_ — 7*p*′ ${\;}^{4}D_{1/2}^{{}^{\circ}}$
5889.87	60	16973.60	5*s*″ ^2^P_1/2_ — 6*p* ${\;}^{4}D_{3/2}^{{}^{\circ}}$
5894.28	18	16960.90	5*p* ${\;}^{4}P_{1/2}^{{}^{\circ}}$ — 8*d* ^4^D_3/2_
5898.32 5898.51	$\begin{array}{l} \;\\ \; \end{array}\}\begin{array}{c} 8\\ 8d \end{array}$	16949.28 16948.74	$\begin{array}{l} \;\\ \; \end{array}\}\;\left({\;}^{1}D_{2}\right)5s{\;}^{2}D_{5/2}—7p\prime {\;}^{4}S_{3/2}^{{}^{\circ}}$
5898.96	90	16947.44	5*p* ${\;}^{4}P_{3/2}^{{}^{\circ}}$ — 7*d* ^4^F_5/2_
5905.45	900	16928.82	$\left\{ \begin{array}{l} 5p{\;}^{4}P_{3/2}^{{}^{\circ}}—\left({\;}^{1}S_{0}\right)5s{\;}^{2}S_{1/2}\\ 5p{\;}^{4}D_{3/2}^{{}^{\circ}}—\left({\;}^{1}D_{2}\right)4d{\;}^{2}F_{5/2} \end{array}\right.$
c 5905.66	80	16928.22	(^1^D_2_)5*s* ^2^D_5/2_ — 7*p*′ ${\;}^{2}D_{3/2}^{{}^{\circ}}$
5909.31	250	16917.76	5*p* ${\;}^{4}P_{3/2}^{{}^{\circ}}$ — 7*d* ^4^F_3/2_
5937.85	90	16836.45	5*p* ${\;}^{4}P_{1/2}^{{}^{\circ}}$ — 8*d* ^4^D_1/2_
5940.48	1600	16828.99	5*s*″ ^2^P_1/2_ — 6*p* ${\;}^{4}P_{1/2}^{{}^{\circ}}$
5943.71	140	16819.85	5*p* ${\;}^{4}D_{5/2}^{{}^{\circ}}$ — (^1^D_2_) 4*d* ^2^D_3/2_
5945.50	600	16814.78	5*p* ${\;}^{4}P_{3/2}^{{}^{\circ}}$ — 7*d* ^4^P_1/2_
5950.32	750	16801.16	5*p* ${\;}^{4}P_{3/2}^{{}^{\circ}}$ — 7*d* ^4^D_5/2_
5953.92	750	16791.01	5*p* ${\;}^{4}P_{3/2}^{{}^{\circ}}$ — 7*d* ^4^D_3/2_
5982.90	12	16709.67	5*p* ${\;}^{4}D_{3/2}^{{}^{\circ}}$ — 6*d*′ ^4^P_3/2_
5983.35	5	16708.42	5*p* ${\;}^{4}D_{3/2}^{{}^{\circ}}$ — 9*a* ^4^P_1/2_
5985.32	125	16702.92	5*p* ${\;}^{4}P_{1/2}^{{}^{\circ}}$ — (^1^D_2_) 4*d* ^2^D_3/2_
5992.89	100	16681.82	5*p* ${\;}^{4}P_{3/2}^{{}^{\circ}}$ — 7*d* ^4^D_1/2_
5999.73	95	16662.80	5*p* ${\;}^{4}D_{3/2}^{{}^{\circ}}$ — 9*d* ^4^D_5/2_
6007.96	200	16639.98	5*p* ${\;}^{4}D_{7/2}^{{}^{\circ}}$ — 9*s* ^4^P_5/2_
6018.17	200	16611.75	5*p* ${\;}^{4}P_{5/2}^{{}^{\circ}}$ — 7*s*′ ^2^P_3/2_
6022.66	30	16599.36	5*p* ${\;}^{4}D_{3/2}^{{}^{\circ}}$ — 6*d*′ ^2^F_5/2_
6025.24	10*h*	16592.25	5*p* ${\;}^{4}D_{3/2}^{{}^{\circ}}$ — 9*d* ^4^D_3/2_
6057.87	200	16502.88	5*p* ${\;}^{4}P_{5/2}^{{}^{\circ}}$ — 5*d*″ ^2^D_5/2_
6064.35	200	16485.25	5*p* ${\;}^{4}D_{5/2}^{{}^{\circ}}$ — 9*s* ^4^P_3/2_
6067.36	5	16477.07	5*p* ${\;}^{4}D_{7/2}^{{}^{\circ}}$ — (^1^D_2_) 4*d* ^2^G_7/2_
6095.74	700	16400.36	5*p* ${\;}^{4}D_{7/2}^{{}^{\circ}}$ — (^1^D_2_) 4*d* ^2^G_9/2_
6107.69	125	16368.27	5*p* ${\;}^{4}P_{1/2}^{{}^{\circ}}$ — 9*s* ^4^P_3/2_
6111.10	80	16359.14	5*p* ${\;}^{4}D_{7/2}^{{}^{\circ}}$ — 7*d* ^4^F_7/2_
6116.19	60	16345.52	5*p* ${\;}^{4}D_{3/2}^{{}^{\circ}}$ — (^1^D_2_) 4*d* ^2^D_5/2_
6118.80	300	16338.55	5*p* ${\;}^{4}P_{3/2}^{{}^{\circ}}$ — 7*s*′ ^4^P_1/2_
6122.14	2400	16329.64	5*p* ${\;}^{4}D_{7/2}^{{}^{\circ}}$ — 7*d* ^4^F_9/2_
6122.35	100	16329.08	5*p* ${\;}^{4}P_{5/2}^{{}^{\circ}}$ — 5*d*″ ^4^D_3/2_
6126.53	30*W*	16317.94	(^1^D_2_)5*s* ^2^D_5/2_ — 6*f* ${\left[4\right]}_{7/2}^{{}^{\circ}}$
6132.71	800	16301.49	5*p* ${\;}^{4}D_{5/2}^{{}^{\circ}}$ — (^1^D_2_) 4*d* ^2^G_7/2_
6134.71	75	16296.18	5*p* ${\;}^{4}D_{3/2}^{{}^{\circ}}$ — 10*s* ^4^P_3/2_
6137.49	150	16288.80	5*p* ${\;}^{4}D_{7/2}^{{}^{\circ}}$ — 7*d* ^4^D_5/2_
6141.04	100*w*	16279.38	(^1^D_2_)5*s* ^2^D_3/2_ — 6*f* ${\left[3\right]}_{5/2}^{{}^{\circ}}$
6142.73	100	16274.90	5*p* ${\;}^{4}P_{3/2}^{{}^{\circ}}$ — 7*s*′ ^4^P_3/2_
6148.60	40000	16259.37	5*s* ^4^P_5/2_ — 5*p*″ ${\;}^{2}P_{3/2}^{{}^{\circ}}$
d 6151.10	50*d*	16252.76	5*p* ${\;}^{4}D_{3/2}^{{}^{\circ}}$ — 10*s* ^4^P_5/2_
6158.19	750	16234.05	5*p* ${\;}^{4}D_{7/2}^{{}^{\circ}}$ — 7*d* ^4^D_7/2_
6177.39	2000	16183.59	5*p* ${\;}^{4}D_{5/2}^{{}^{\circ}}$ — 7*d* ^4^F_7/2_
6184.09	200	16166.05	5*p* ${\;}^{4}P_{3/2}^{{}^{\circ}}$ — 5*d*″ ^4^D_5/2_
6199.74	50	16125.25	5*p* ${\;}^{4}P_{5/2}^{{}^{\circ}}$ — 8*s* ^4^P_3/2_
6203.08	900	16116.56	5*p* ${\;}^{4}P_{5/2}^{{}^{\circ}}$ — 8*s* ^4^P_5/2_
6204.35	90	16113.27	5*p* ${\;}^{4}D_{5/2}^{{}^{\circ}}$ — 7*d* ^4^D_5/2_
6204.49	90	16112.90	5*p* ${\;}^{4}P_{1/2}^{{}^{\circ}}$ — 7*d* ^4^F_3/2_
6205.40	120	16110.54	5*p* ${\;}^{4}D_{3/2}^{{}^{\circ}}$ — 8*d* ^4^F_3/2_
6208.26	100	16103.12	5*p* ${\;}^{4}D_{5/2}^{{}^{\circ}}$ — 7*d* ^4^D_3/2_
6216.71	5	16081.23	5*p* ${\;}^{4}D_{3/2}^{{}^{\circ}}$ — 8*d* ^4^P_1/2_
6225.51	65	16058.50	5*p* ${\;}^{4}D_{5/2}^{{}^{\circ}}$ — 7*d* ^4^D_7/2_
6235.87	10	16031.82	5*p* ${\;}^{4}D_{3/2}^{{}^{\circ}}$ — 8*d* ^4^D_3/2_
6244.39	400	16009.95	5*p* ${\;}^{4}P_{1/2}^{{}^{\circ}}$ 7*d* — ^4^P_1/2_
6248.24	200	16000.08	5*p* ${\;}^{4}D_{3/2}^{{}^{\circ}}$— 8*d* ^4^F_5/2_
6251.32	300	15992.20	5*p* ${\;}^{4}P_{3/2}^{{}^{\circ}}$ — 5*d*″ ^2^D_3/2_
6253.69	400	15986.14	5*p* ${\;}^{4}P_{1/2}^{{}^{\circ}}$ — 7*d* ^4^D_3/2_
6282.46	600	15912.93	5*p* ${\;}^{4}P_{5/2}^{{}^{\circ}}$ — 5*d*′ ^4^D_5/2_
6284.69	8	15907.28	$\left\{ \begin{array}{l} 5p\prime {\;}^{2}S_{1/2}^{{}^{\circ}}—8s\prime {\;}^{4}P_{1/2}\\ 5p{\;}^{4}D_{3/2}^{{}^{\circ}}—8d{\;}^{4}D_{1/2} \end{array}\right.$
6290.13	550	15893.53	5*p* ${\;}^{4}P_{5/2}^{{}^{\circ}}$ — 5*d*′ ^2^F_5/2_
6296.71	700	15876.92	5*p* ${\;}^{4}P_{1/2}^{{}^{\circ}}$ — 7*d* ^4^D_1/2_
6301.36	275	15865.20	5*p* ${\;}^{4}P_{5/2}^{{}^{\circ}}$ — 5*d*′ ^4^P_3/2_
6315.12	5	15830.63	5*p*′ ${\;}^{2}S_{1/2}^{{}^{\circ}}$ — 8*s*′ ^2^P_3/2_
6331.99	300	15788.46	5*p* ${\;}^{4}P_{3/2}^{{}^{\circ}}$ — 8*s* ^4^P_3/2_
6335.48	1500	15779.76	5*p* ${\;}^{4}P_{3/2}^{{}^{\circ}}$ — 8*s* ^4^P_5/2_
6336.39 6336.56	$\begin{array}{l} \;\\ \; \end{array}\}\begin{array}{c} 300\\ 500 \end{array}$	15777.49 15777.07	$\begin{array}{l} \;\\ \; \end{array}\}\;\left({\;}^{1}D_{2}\right)5s{\;}^{2}D_{5/2}—8p{\;}^{4}D_{3/2}^{{}^{\circ}}$
6337.85	60	15773.86	5*p* ${\;}^{4}D_{3/2}^{{}^{\circ}}$ — (^1^D_2_) 4*d* ^2^D_3/2_
6343.79	35*w*	15759.09	(^1^D_2_)5*s* ^2^D_3/2_ — 8*p* ${\;}^{4}D_{3/2}^{{}^{\circ}}$
6345.30	500	15755.34	5*p* ${\;}^{4}P_{5/2}^{{}^{\circ}}$ — 5*d*′ ^4^F_7/2_
6349.82	500	15744.12	5*p* ${\;}^{4}P_{5/2}^{{}^{\circ}}$ — 6*d* ^4^F_3/2_
6350.73	60000	15741.87	5*s* ^4^P_5/2_ — 5*p*′ ${\;}^{4}S_{3/2}^{{}^{\circ}}$
6371.60	200*W*	15690.31	(^1^D_2_)5*s* ^2^D_3/2_ — 8*p* ${\;}^{4}P_{1/2}^{{}^{\circ}}$
6392.57	35*W*	15638.84	(^1^D_2_)5*s* ^2^D_5/2_ — 8*p* ${\;}^{4}P_{3/2}^{{}^{\circ}}$
6398.03	30	15625.49	5*p*′ ${\;}^{4}S_{1/2}^{{}^{\circ}}$ — (^1^D_2_) 4*d* ^2^S_1/2_
6399.99	10*w*	15620.71	(^1^D_2_)5*s* ^2^D_3/2_ — 8*p* ${\;}^{4}P_{3/2}^{{}^{\circ}}$
6405.52 6405.58 6405.68 6405.80	$\begin{array}{l} \;\\ \;\\ \;\\ \; \end{array}\}\begin{array}{c} 3\\ \begin{array}{l} 4\\ 6\\ 8 \end{array} \end{array}$	15607.22 15607.07 15606.83 15606.54	$\begin{array}{l} \;\\ \; \end{array}\}\;\left({\;}^{1}D_{2}\right)5s{\;}^{2}D_{5/2}—4f\prime \;{\left[4\right]}_{7/2}^{{}^{\circ}}$
6410.32	2500	15595.53	5*p* ${\;}^{4}P_{5/2}^{{}^{\circ}}$ — 6*d* ^4^F_7/2_
6418.29	45	15576.17	5*p* ${\;}^{4}P_{3/2}^{{}^{\circ}}$ — 5*d*′ ^4^P_5/2_
6418.96 6419.03	$\begin{array}{l} \;\\ \; \end{array}\}\begin{array}{c} 15s\\ 15 \end{array}$	15574.54 15574.37	$\begin{array}{l} \;\\ \; \end{array}\}\;\left({\;}^{1}D_{2}\right)5s{\;}^{2}D_{3/2}—4f\prime \;{\left[2\right]}_{5/2}^{{}^{\circ}}$
6426.30	500	15556.75	5*p* ${\;}^{4}P_{3/2}^{{}^{\circ}}$ — 5*d*′ ^2^F_5/2_
6435.81	15	15533.77	5*p* ${\;}^{4}P_{1/2}^{{}^{\circ}}$ — 7*s*′ ^4^P_1/2_
6436.60	10	15531.86	
6438.02	600	15528.43	5*p* ${\;}^{4}P_{3/2}^{{}^{\circ}}$ — 5*d*′ ^4^F_3/2_
6458.92	25*h*	15478.19	5*p* ${\;}^{4}D_{5/2}^{{}^{\circ}}$ — 5*d*″ ^2^D_5/2_
6462.32	500	15470.04	5*p* ${\;}^{4}P_{1/2}^{{}^{\circ}}$ — 7*d*′ ^2^P_3/2_
6470.41	200	15450.70	5*p* ${\;}^{4}P_{3/2}^{{}^{\circ}}$ — 5*d*′ ^2^P_3/2_
6475.23	100*h*	15439.20	5*p* ${\;}^{4}D_{3/2}^{{}^{\circ}}$ — 9*s* ^4^P_3/2_
6483.56	1800	15419.36	5*p* ${\;}^{4}P_{3/2}^{{}^{\circ}}$ — 6*d* ^4^P_1/2_
6483.96	35	15418.41	5*p* ${\;}^{4}D_{3/2}^{{}^{\circ}}$ — 9*s* ^4^P_5/2_
6488.62	800	15407.34	5*p* ${\;}^{4}P_{3/2}^{{}^{\circ}}$ — 6*d* ^4^F_3/2_
6493.80	20*h*	15395.05	5*p*′ ${\;}^{2}D_{5/2}^{{}^{\circ}}$ — 8*s*′ ^2^P_3/2_
6501.51	4	15376.79	5*p*′ ${\;}^{2}S_{1/2}^{{}^{\circ}}$ — 6*d*′ ^4^P_3/2_
6514.32	12	15346.56	5*p* ${\;}^{4}D_{3/2}^{{}^{\circ}}$ — 7*s*″ ^2^P_1/2_
6514.62	1000	15345.85	5*p* ${\;}^{4}P_{3/2}^{{}^{\circ}}$ — 6*d* ^4^F_5/2_
b 6531.39	70	15306.45	5*p*′ ${\;}^{2}D_{3/2}^{{}^{\circ}}$ — 8*s*′ ^2^P_1/2_
6532.29	600	15304.34	5*p* ${\;}^{4}D_{5/2}^{{}^{\circ}}$ — 5*d*″ ^2^D_3/2_
6541.30	600	15283.26	5*p* ${\;}^{4}P_{3/2}^{{}^{\circ}}$ — 5*d*′ ^2^P_1/2_
6544.57	20000	15275.62	5*p* ${\;}^{4}P_{5/2}^{{}^{\circ}}$ — 6*d* ^4^D_5/2_
6548.09	1500	15267.41	5*p* ${\;}^{4}D_{7/2}^{{}^{\circ}}$ — 8*s* ^4^P_5/2_
6551.57	12	15259.30	5*p*′ ${\;}^{2}S_{1/2}^{{}^{\circ}}$ — 9*d* ^4^D_3/2_
6559.80	50000*cw*	15240.16	5*s* ^4^P_5/2_ — 5*p′* ${\;}^{2}D_{3/2}^{{}^{\circ}}$
6571.31	1000	15213.46	5*p* ${\;}^{4}D_{3/2}^{{}^{\circ}}$ — 7*d* ^4^F_5/2_
6574.29	15	15206.57	5*p*′ ${\;}^{2}S_{1/2}^{{}^{\circ}}$ — 9*d* ^4^D_1/2_
6576.24	10*W*	15202.06	
6579.14	1800	15195.36	5*p* ${\;}^{4}P_{5/2}^{{}^{\circ}}$— 6*d* ^4^D_3/2_
6579.36	300	15194.85	5*p* ${\;}^{4}D_{3/2}^{{}^{\circ}}$ — (^1^S_0_) 5*s* ^4^S_1/2_
6582.17	20000	15188.36	5*p* ${\;}^{4}P_{5/2}^{{}^{\circ}}$ — 6*d* ^4^D_7/2_
6582.62	35	15187.32	5*p* ${\;}^{4}P_{1/2}^{{}^{\circ}}$ — 5*d*″ ^2^D_3/2_
6584.14	600	15183.82	5*p* ${\;}^{4}D_{3/2}^{{}^{\circ}}$ — 7*d* ^4^F_3/2_
6589.62	10*w*	15171.19	5*p*′ ${\;}^{2}D_{5/2}^{{}^{\circ}}$ — (^1^D_2_) 4*d* ^2^F_7/2_
6604.80	15	15136.32	4*d* ^4^D_7/2_ — 10*f* ${\left[4\right]}_{9/2}^{{}^{\circ}}$
6613.05	4	15117.44	5*p*′ ${\;}^{4}D_{1/2}^{{}^{\circ}}$ — 8*s*′ ^4^P_1/2_
6620.47	1500	15100.50	5*p* ${\;}^{4}D_{5/2}^{{}^{\circ}}$ — 8*s* ^4^P_3/2_
6621.44	8	15098.29	5*p*′ ${\;}^{2}D_{5/2}^{{}^{\circ}}$ — (^1^D_2_) 4*d* ^2^P_3/2_
6624.26	20	15091.86	5*p* ${\;}^{4}D_{5/2}^{{}^{\circ}}$ — 8*s* ^4^P_5/2_
6629.09	20	15080.86	5*p* ${\;}^{4}D_{3/2}^{{}^{\circ}}$ — 7*d* ^4^P_1/2_
6631.62	50000*cw*	15075.11	5*s* ^4^P_5/2_ — 5*p′* ${\;}^{2}D_{5/2}^{{}^{\circ}}$
6635.10	60	15067.20	5*p* ${\;}^{4}D_{3/2}^{{}^{\circ}}$ — 7*d* ^4^D_5/2_
6636.62	150	15063.75	5*p* ${\;}^{4}D_{7/2}^{{}^{\circ}}$ — 5*d*′ ^4^P_5/2_
6639.57	40	15057.06	5*p* ${\;}^{4}D_{3/2}^{{}^{\circ}}$ — 7*d* ^4^D_3/2_
6645.17	40	15044.37	5*p* ${\;}^{4}D_{7/2}^{{}^{\circ}}$ — 5*d*′ ^2^F_5/2_
6646.59	120	15041.16	5*p*′ ${\;}^{2}S_{1/2}^{{}^{\circ}}$ — 6*d*′ ^2^P_3/2_
6653.82	15	15024.81	5*p*′ ${\;}^{2}D_{3/2}^{{}^{\circ}}$ — (^1^D_2_) 4*d* ^2^S_1/2_
6666.93	150	14995.27	5*p*′ ${\;}^{2}D_{3/2}^{{}^{\circ}}$ — (^1^D_2_) 4*d* ^2^F_5/2_
6672.15	600	14983.54	5*p* ${\;}^{4}P_{1/2}^{{}^{\circ}}$ — 8*s* ^4^P_3/2_
6676.54	15	14973.68	$\left\{ \begin{array}{l} 4d{\;}^{4}D_{7/2}—5f\prime \;{\left[3\right]}_{5/2}^{{}^{\circ}}\\ 5p\prime {\;}^{2}D_{3/2}^{{}^{\circ}}—\left({\;}^{1}D_{2}\right)4d{\;}^{2}P_{1/2} \end{array}\right.$
6676.72	8	14973.28	4*d* ^4^D_7/2_ — 5*f′* ${\left[3\right]}_{7/2}^{{}^{\circ}}$
6681.71	15	14962.10	5*p*′ ${\;}^{2}D_{5/2}^{{}^{\circ}}$ — 6*d*′ ^4^P_5/2_
6682.28	20000	14960.82	5*s* ^4^P_3/2_ — 5*p*″ ${\;}^{2}P_{1/2}^{{}^{\circ}}$
6684.17 6684.23 6684.33	$\begin{array}{l} \;\\ \;\\ \; \end{array}\}\begin{array}{c} 50\\ \begin{array}{l} 50\\ 70 \end{array} \end{array}$	14956.59 14956.46 14956.23	$\begin{array}{l} \;\\ \; \end{array}\}\;\left({\;}^{1}D_{2}\right)5s{\;}^{2}D_{5/2}—5f\;{\left[4\right]}_{5/2}^{{}^{\circ}}$
6687.33	90	14949.52	4*d* ^4^D_7/2_ — 5*f′* ${\left[4\right]}_{9/2}^{{}^{\circ}}$
6688.08	90	14947.85	5*p* ${\;}^{2}D_{3/2}^{{}^{\circ}}$ — 7*d* ^4^D_1/2_
6690.35	120*cw*	14942.78	(^1^D_2_)5*s* ^2^D_3/2_ — 5*f* ${\left[3\right]}_{7/2}^{{}^{\circ}}$
6692.13	10000	14938.80	5*p* ${\;}^{4}P_{3/2}^{{}^{\circ}}$ — 6*d* ^4^D_5/2_
6694.62	10	14933.24	5*p*′ ${\;}^{2}D_{3/2}^{{}^{\circ}}$ — (^1^D_2_) 4*d* ^2^P_3/2_
6700.71	60*h*	14919.67	5*p*′ ${\;}^{2}D_{5/2}^{{}^{\circ}}$ — 9*d* ^4^F_7/2_
6702.07	110	14916.65	5*p*′ ${\;}^{2}S_{1/2}^{{}^{\circ}}$ — 6*d′* ^2^P_1/2_
6706.79	60	14906.15	5*p* ${\;}^{2}D_{7/2}^{{}^{\circ}}$ — 5*d*′ ^2^F_7/2_
6712.12	30	14894.31	5*p′* ${\;}^{2}D_{5/2}^{{}^{\circ}}$ — 9*d* ^4^D_5/2_
6713.06	125	14892.23	5*p′* ${\;}^{2}D_{5/2}^{{}^{\circ}}$ — 9*d* ^4^D_7/2_
6714.86	90	14888.23	5*p* ${\;}^{2}D_{5/2}^{{}^{\circ}}$ — 5*d*′ ^4^P_5/2_
6719.97	8	14876.91	
6720.68	75*h*	14875.34	4*d* ^4^D_7/2_ — 9*f* ${\left[4\right]}_{9/2}^{{}^{\circ}}$
6723.65	400	14868.77	5*p* ${\;}^{4}D_{5/2}^{{}^{\circ}}$ — 5*d*′ ^2^F_5/2_
6728.28	8000	14858.54	5*p* ${\;}^{4}P_{3/2}^{{}^{\circ}}$ — 6*d* ^2^D_3/2_
6738.61	100	14835.76	5*p*′ ${\;}^{4}D_{1/2}^{{}^{\circ}}$ — (^1^D_2_) 4*d* ^2^S_1/2_
6739.66	8	14833.45	5*p* ${\;}^{4}D_{7/2}^{{}^{\circ}}$ — 6*d* ^4^F_5/2_
6740.83	5	14830.87	5*p*′ ${\;}^{2}D_{5/2}^{{}^{\circ}}$ — 6*d*′ ^2^F_5/2_
6752.67	6	14804.87	5*p*′ ${\;}^{4}S_{3/2}^{{}^{\circ}}$ — 8*s*′ ^4^P_1/2_
6760.06	2000	14788.69	5*s* ^4^P_3/2_ — 5*p*″ ${\;}^{2}P_{3/2}^{{}^{\circ}}$
6761.92	25	14784.62	5*p*′ ${\;}^{4}D_{1/2}^{{}^{\circ}}$ — (^1^D_2_) 4*d* ^2^P_1/2_
6765.12	8	14777.62	5*p*′ ${\;}^{2}S_{1/2}^{{}^{\circ}}$ — 8*d* ^4^F_3/2_
6771.95	175	14762.72	5*p* ${\;}^{4}D_{5/2}^{{}^{\circ}}$ — 5*d*′ ^2^P_3/2_
6774.63	30*h*	14756.88	4*d* ^4^D_5/2_ — 5*f*′ ${\left[3\right]}_{7/2}^{{}^{\circ}}$
6778.57	60	14748.30	5*p*′ ${\;}^{2}S_{1/2}^{{}^{\circ}}$ — 8*d* ^4^P_1/2_
6779.48	2000	14746.32	5*p* ${\;}^{4}D_{7/2}^{{}^{\circ}}$ — 5*d*′ ^4^F_7/2_
6785.23	10	14733.83	4*d* ^4^D_5/2_ — 5*f*′ ${\left[4\right]}_{7/2}^{{}^{\circ}}$
6785.74	900	14732.72	5*p*′ ${\;}^{2}D_{5/2}^{{}^{\circ}}$ — 6*d*′ ^2^F_7/2_
6786.74	2200	14730.55	5*p* ${\;}^{2}D_{5/2}^{{}^{\circ}}$ — 5*d*′ ^4^F_7/2_
6787.34	175	14729.25	5*p* ${\;}^{2}D_{3/2}^{{}^{\circ}}$ — 9*d* ^4^D_5/2_
6787.77	8	14728.31	5*p*′ ${\;}^{4}S_{3/2}^{{}^{\circ}}$ — 8*s*′ ^2^P_3/2_
6790.04	6500	14723.39	5*p* ${\;}^{4}D_{7/2}^{{}^{\circ}}$ — 6*d* ^4^F_9/2_
6791.48	1600*c*	14720.27	5*p* ${\;}^{4}P_{3/2}^{{}^{\circ}}$ — 6*d* ^4^D_1/2_
6801.35	60	14698.91	5*p*′ ${\;}^{2}S_{1/2}^{{}^{\circ}}$ — 8*d* ^4^D_3/2_
6816.72	50	14665.76	5*p*′ ${\;}^{2}D_{3/2}^{{}^{\circ}}$ — 6*d*′ ^2^F_5/2_
6820.04	6*h*	14658.63	5*p*′ ${\;}^{2}D_{3/2}^{{}^{\circ}}$ — 9*d* ^4^D_3/2_
6820.39	800	14657.87	5*p* ${\;}^{4}D_{5/2}^{{}^{\circ}}$ — 6*d* ^4^F_5/2_
6826.02	400	14645.78	5*p* ${\;}^{4}P_{1/2}^{{}^{\circ}}$ — 5*d*′ ^2^P_3/2_
6840.62	175	14614.53	5*p* ${\;}^{4}P_{1/2}^{{}^{\circ}}$ — 6*d* ^4^P_1/2_
6844.82	5	14605.56	5*p′* ${\;}^{4}D_{5/2}^{{}^{\circ}}$ — 6*d*′ ^2^P_3/2_
6845.27	40*c*	14604.60	5*p* ${\;}^{4}D_{3/2}^{{}^{\circ}}$ — 7*s*′ ^4^P_1/2_
6846.27	150	14602.46	5*p* ${\;}^{4}P_{1/2}^{{}^{\circ}}$ — 6*d* ^4^F_3/2_
6853.51	3	14587.04	5*p*′ ${\;}^{4}D_{1/2}^{{}^{\circ}}$ — 8*f* ^4^P_3/2_
6858.22	45	14577.02	5*p*′ ${\;}^{2}D_{5/2}^{{}^{\circ}}$ — (^1^D_2_) 4*d* ^2^D_5/2_
6859.43	20	14574.45	5*p*′ ${\;}^{2}S_{1/2}^{{}^{\circ}}$ — 8*d* ^4^D_1/2_
6861.15	1800	14570.80	5*p* ${\;}^{4}D_{5/2}^{{}^{\circ}}$ — 6*d* ^4^F_7/2_
6875.22	20*w*	14540.98	5*p* ${\;}^{4}D_{3/2}^{{}^{\circ}}$ — 7*s*′ ^2^P_3/2_
6887.99	12*h*	14514.02	4*d* ^4^D_7/2_ — 8*f* ${\left[3\right]}_{7/2,\;5/2}^{{}^{\circ}}$
6888.73	80*h*	14512.46	4*d* ^4^D_7/2_ — 8*f* ${\left[4\right]}_{9/2}^{{}^{\circ}}$
6904.95	400	14478.37	5*p* ${\;}^{4}P_{1/2}^{{}^{\circ}}$ — 5*d*′ ^2^P_1/2_
6922.86	30	14440.91	5*p*′ ${\;}^{2}S_{1/2}^{{}^{\circ}}$ — (^1^D_2_) 4*d* ^2^D_3/2_
6923.04	1	14440.54	5*p*′ ${\;}^{2}D_{3/2}^{{}^{\circ}}$ — 6*d*′ ^2^P_3/2_
6927.10	2	14432.07	5*p* ${\;}^{4}D_{3/2}^{{}^{\circ}}$ — 5*d*″ ^2^D_5/2_
6927.34	8	14431.57	5*p*′ ${\;}^{4}S_{3/2}^{{}^{\circ}}$ — (^1^D_2_) 4*d* ^2^P_3/2_
6929.78	400	14426.49	5*p* ${\;}^{4}D_{7/2}^{{}^{\circ}}$ — 6*d* ^4^D_5/2_
6936.76	20	14411.98	5*p*′ ${\;}^{2}D_{3/2}^{{}^{\circ}}$ — (^1^D_2_) 4*d* ^2^D_5/2_
6962.99	10	14357.69	4*d* ^4^D_3/2_ — 5*f*′ ${\left[2\right]}_{5/2}^{{}^{\circ}}$
6971.96	250	14339.21	5*p* ${\;}^{4}D_{7/2}^{{}^{\circ}}$ — 6*d* ^4^D_7/2_
6992.25	5	14297.60	4*d* ^4^D_5/2_ — 8*f* ${\left[3\right]}_{7/2,\;5/2}^{{}^{\circ}}$
6992.83	5	14296.42	4*d* ^4^D_5/2_ — 8*f* ${\left[4\right]}_{7/2}^{{}^{\circ}}$
6993.31	20	14295.44	5*p*′ ${\;}^{4}S_{3/2}^{{}^{\circ}}$ — 6*d*′ ^4^P_5/2_
7005.19	10000	14271.19	5*s* ^4^P_3/2_ — 5*p*′ ${\;}^{4}S_{3/2}^{{}^{\circ}}$
7011.53	50	14258.29	5*p* ${\;}^{4}D_{3/2}^{{}^{\circ}}$ — 5*d*″ ^2^D_3/2_
7015.15	75	14250.93	5*p* ${\;}^{4}D_{5/2}^{{}^{\circ}}$ — 6*d* ^4^D_5/2_
7024.70	20	14231.56	5*p′* ${\;}^{2}D_{5/2}^{{}^{\circ}}$ — 8*d* ^4^F_5/2_
7026.53	2	14227.85	
7031.36	15	14218.08	5*p′* ${\;}^{2}D_{5/2}^{{}^{\circ}}$ — 8*d* ^4^D_7/2_
7054.88	50	14170.68	5*p* ${\;}^{4}D_{5/2}^{{}^{\circ}}$ — 6*d* ^4^D_3/2_
7058.38	200	14163.65	5*p* ${\;}^{4}D_{5/2}^{{}^{\circ}}$ — 6*d* ^4^D_7/2_
7061.71	1	14156.97	5*p′* ${\;}^{4}S_{3/2}^{{}^{\circ}}$ — 9*d* ^4^D_3/2_
7066.33	4	14147.72	5*p*′ ${\;}^{2}D_{3/2}^{{}^{\circ}}$ — 8*d* ^4^P_1/2_
7076.71	5	14126.97	5*p*′ ${\;}^{4}D_{1/2}^{{}^{\circ}}$ — 6*d*′ ^2^P_1/2_
7082.63	3	14115.16	5*p*″ ${\;}^{2}P_{1/2}^{{}^{\circ}}$ — 8*s*′ ^4^P_1/2_
7091.12	4	14098.26	5*p*′ ${\;}^{2}D_{3/2}^{{}^{\circ}}$ — 8*d* ^4^D_3/2_
7101.80	5*h*	14077.06	5*p*′ ${\;}^{2}D_{3/2}^{{}^{\circ}}$ — 8*d* ^4^D_5/2_
7107.14	1	14066.48	5*p*′ ${\;}^{2}D_{3/2}^{{}^{\circ}}$ — 8*d* ^4^F_5/2_
7111.50 7111.62 7111.73	$\begin{array}{l} \;\\ \;\\ \; \end{array}\}\begin{array}{c} 100\\ \begin{array}{l} 100\\ 150 \end{array} \end{array}$	14057.86 14057.62 14057.40	$\begin{array}{l} \;\\ \; \end{array}\}\;\left({\;}^{1}D_{2}\right)5s{\;}^{2}D_{5/2}—6p\prime \prime {\;}^{2}P_{3/2}^{{}^{\circ}}$
7113.22	50	14054.46	5*p* ${\;}^{4}D_{3/2}^{{}^{\circ}}$ — 8*s* ^4^P_3/2_
7113.60	100	14053.71	5*p* ${\;}^{2}P_{1/2}^{{}^{\circ}}$ — 6*d* ^4^D_3/2_
7117.59	15	14045.83	5*p* ${\;}^{4}D_{3/2}^{{}^{\circ}}$ — 8*s* ^4^P_5/2_
7120.78 7120.88	$\begin{array}{l} \;\\ \; \end{array}\}\begin{array}{c} 10\\ 15 \end{array}$	14039.54 14039.34	$\begin{array}{l} \;\\ \; \end{array}\}\;\left({\;}^{1}D_{2}\right)5s{\;}^{2}D_{3/2}—6p\prime \prime {\;}^{2}P_{3/2}^{{}^{\circ}}$
7133.95	5	14013.62	5*p*′ ${\;}^{2}S_{1/2}^{{}^{\circ}}$ — 7*s*″ ^2^P_1/2_
7138.05	2	14005.57	5*p*″ ${\;}^{2}P_{3/2}^{{}^{\circ}}$ — (^1^D_2_) 4*d* ^2^S_1/2_
7138.19	4	14005.29	5*p*′ ${\;}^{2}D_{5/2}^{{}^{\circ}}$ — (^1^D_2_) 4*d* ^2^D_3/2_
7142.17 7142.30	$\begin{array}{l} \;\\ \; \end{array}\}\begin{array}{c} 300\\ 400 \end{array}$	13997.49 13997.23	$\begin{array}{l} \;\\ \; \end{array}\}\;\left({\;}^{1}D_{2}\right)5s{\;}^{2}D_{3/2}—6p\prime \prime {\;}^{2}P_{1/2}^{{}^{\circ}}$
7145.56	2	13990.85	4*d* ^4^D_7/2_ — 7*f* ${\left[2\right]}_{5/2}^{{}^{\circ}}$
7147.07	4	13987.89	5*p*′ ${\;}^{4}D_{1/2}^{{}^{\circ}}$ — 8*d* ^4^F_3/2_
7149.08	15	13983.96	4*d* ^4^D_7/2_ — 7*f* ${\left[3\right]}_{7/2,\;5/2}^{{}^{\circ}}$
7150.00	8	13982.16	4*d* ^4^D_7/2_ — 7*f* ${\left[4\right]}_{7/2}^{{}^{\circ}}$
7150.30	75	13981.57	4*d* ^4^D_7/2_ — 7*f* ${\left[4\right]}_{9/2}^{{}^{\circ}}$
7153.13	5*h*	13976.04	5*p*″ ${\;}^{2}P_{3/2}^{{}^{\circ}}$ — (^1^D_2_) 4*d* ^4^F_5/2_
7160.74	15	13961.19	4*d* ^4^D_1/2_ — 5*f*′ ${\left[2\right]}_{3/2}^{{}^{\circ}}$
7162.10	750	13958.54	5*s* ^4^P_3/2_ — 5*p*′ ${\;}^{4}D_{1/2}^{{}^{\circ}}$
7172.22	25	13938.84	5*p*′ ${\;}^{4}S_{3/2}^{{}^{\circ}}$ — 6*d*′ ^2^P_3/2_
7175.74	5*H*	13932.00	4*d* ^4^D_3/2_ — 8*f* ${\left[2\right]}_{3/2}^{{}^{\circ}}$
7177.89	5*h*	13927.83	4*d* ^4^D_3/2_ — 8*f* ${\left[3\right]}_{5/2}^{{}^{\circ}}$
7184.30	300	13915.41	5*p* ${\;}^{4}P_{1/2}^{{}^{\circ}}$ — 6*d* ^4^D_1/2_
7194.24 7194.30 7194.41 7194.53	$\begin{array}{l} \;\\ \;\\ \; \end{array}\}\begin{array}{c} 3\\ \begin{array}{l} 5\\ 7\\ 15 \end{array} \end{array}$	13896.18 13896.06 13895.85 13895.62	$\begin{array}{l} \;\\ \; \end{array}\}\;\left({\;}^{1}D_{2}\right)5s{\;}^{2}D_{5/2}—7p{\;}^{4}D_{3/2}^{{}^{\circ}}$
7214.95	5*c*	13856.29	(^1^D_2_)5*s* ^2^D_3/2_ — 7*p* ${\;}^{4}P_{1/2}^{{}^{\circ}}$
7217.78	20	13850.86	5*p*′ ${\;}^{2}S_{1/2}^{{}^{\circ}}$ — 7*d* ^4^F_3/2_
7222.31	50	13842.17	5*p* ${\;}^{4}D_{3/2}^{{}^{\circ}}$ — 5*d*′ ^4^P_5/2_
7232.45	100	13822.76	5*p* ${\;}^{4}D_{3/2}^{{}^{\circ}}$ — 5*d*′ ^2^F_5/2_
7236.86	7	13814.34	5*p*′ ${\;}^{4}S_{3/2}^{{}^{\circ}}$ — 6*d*′ ^2^P_1/2_
7247.29	5*h*	13794.46	5*p* ${\;}^{4}D_{3/2}^{{}^{\circ}}$ — 5*d*′ ^4^P_3/2_
7255.56	15*cw*	13778.74	(^1^D_2_)5*s* ^2^D_5/2_ — 7*p* ${\;}^{4}D_{5/2}^{{}^{\circ}}$
7257.82	10*h*	13774.45	4*d* ^4^D_5/2_ — 7*f* ${\left[2\right]}_{5/2}^{{}^{\circ}}$
7260.45	2000	13769.46	5*s* ^4^P_3/2_ — 5*p*′ ${\;}^{2}D_{3/2}^{{}^{\circ}}$
7261.46	20	13767.54	4*d* ^4^D_5/2_ — 7*f* ${\left[3\right]}_{7/2,\;5/2}^{{}^{\circ}}$
7262.40	25	13765.76	4*d* ^4^D_5/2_ — 7*f* ${\left[4\right]}_{7/2}^{{}^{\circ}}$
7265.16	5	13760.53	(^1^D_2_)5*s* ^2^D_3/2_ — 7*p* ${\;}^{4}D_{5/2}^{{}^{\circ}}$
7272.58	3*w*	13746.49	(^1^D_2_)5*s* ^2^D_5/2_ — 7*p* ${\;}^{4}D_{7/2}^{{}^{\circ}}$
7284.41	5	13724.17	5*p*′ ${\;}^{2}S_{1/2}^{{}^{\circ}}$ — 7*d* ^4^D_3/2_
7288.40	150	13716.65	5*p* ${\;}^{2}D_{3/2}^{{}^{\circ}}$ — 5*d*′ ^2^P_3/2_
7291.92	1	13710.03	5*p*″ ${\;}^{2}P_{3/2}^{{}^{\circ}}$ — 9*d* ^4^D_5/2_
7305.03	75	13685.43	5*p* ${\;}^{4}D_{3/2}^{{}^{\circ}}$ — 6*d* ^4^P_1/2_
7310.45	4	13675.28	5*p*′ ${\;}^{4}S_{3/2}^{{}^{\circ}}$ — 8*d* ^4^F_3/2_
7311.48	100*d*	13673.35	5*p* ${\;}^{4}D_{3/2}^{{}^{\circ}}$ — 6*d* ^4^F_3/2_
7319.31 7319.41 7319.53	$\begin{array}{l} \;\\ \;\\ \; \end{array}\}\begin{array}{c} 20\\ \begin{array}{l} 25\\ 30 \end{array} \end{array}$	13658.73 13658.54 13658.32	$\begin{array}{l} \;\\ \; \end{array}\}\;\left({\;}^{1}D_{2}\right)5s{\;}^{2}D_{5/2}—7p{\;}^{4}P_{5/2}^{{}^{\circ}}$
7323.35	10	13651.19	5*p*′ ${\;}^{4}D_{1/2}^{{}^{\circ}}$ — (^1^D_2_) 4*d* ^2^D_3/2_
7329.20	10*hw*	13640.30	(^1^D_2_)5*s* ^2^D_3/2_ — 7*p* ${\;}^{4}P_{5/2}^{{}^{\circ}}$
7329.68	1	13639.40	5*p*″ ${\;}^{2}P_{3/2}^{{}^{\circ}}$ — 9*d* ^4^D_3/2_
7333.65 7333.77	$\begin{array}{l} \;\\ \; \end{array}\}\begin{array}{c} 20s\\ 25 \end{array}$	13632.02 13631.80	$\begin{array}{l} \;\\ \; \end{array}\}\;\left({\;}^{1}D_{2}\right)5s{\;}^{2}D_{3/2}—7p{\;}^{4}P_{3/2}^{{}^{\circ}}$
7342.85	10	13614.94	5*p*′ ${\;}^{2}S_{1/2}^{{}^{\circ}}$ — 7*d* ^4^D_1/2_
7344.53	100	13611.83	5*p*′ ${\;}^{4}D_{3/2}^{{}^{\circ}}$ — 6*d* ^4^F_5/2_
7348.51	10000	13604.45	5*s* ^4^P_3/2_ — 5*p*′ ${\;}^{2}D_{5/2}^{{}^{\circ}}$
7378.43	20*d*	13549.29	5*p* ${\;}^{4}D_{3/2}^{{}^{\circ}}$ — 5*d*′ ^2^P_1/2_
7383.69	5	13539.63	4*d* ^4^D_7/2_ — 9*p* ${\;}^{4}P_{5/2}^{{}^{\circ}}$
7385.54	4*h*	13536.24	4*d* ^4^D_1/2_ — 8*f* ${\left[2\right]}_{3/2}^{{}^{\circ}}$
7420.69	2	13472.13	5*p*′ ${\;}^{4}S_{3/2}^{{}^{\circ}}$ — 8*d* ^4^D_1/2_
7425.85	750	13462.76	5*p* ${\;}^{4}P_{5/2}^{{}^{\circ}}$ — 7*s* ^4^P_5/2_
7452.13	60	13415.29	5*p*′ ${\;}^{2}D_{5/2}^{{}^{\circ}}$ — 7*d* ^4^F_3/2_
7453.43	70	13412.95	5*p*′ ${\;}^{2}D_{3/2}^{{}^{\circ}}$ — 7*s*″ ^2^P_½_
7458.23	50*h*	13404.32	4*d* ^4^D_3/2_ — 7*f* ${\left[2\right]}_{3/2}^{{}^{\circ}}$
7461.86	70	13397.79	4*d* ^4^D_3/2_ — 7*f* ${\left[3\right]}_{5/2}^{{}^{\circ}}$
7464.68	8	13392.73	5*p*″ ${\;}^{2}P_{3/2}^{{}^{\circ}}$ — (^1^D_2_) 4*d* ^2^D_5/2_
7495.46	15*hw*	13337.74	4*d* ^4^F_9/2_ — 9*f* ${\left[5\right]}_{1\overset{{}^{\circ}}{1}/2}$
7497.81	5	13333.56	4*d* ^2^D_5/2_ — 9*p* ${\;}^{4}P_{3/2}^{{}^{\circ}}$
7512.96	40000	13306.67	5*s* ^4^P_5/2_ — 5*p* ${\;}^{4}D_{3/2}^{{}^{\circ}}$
7532.77	50	13271.67	5*p*′ ${\;}^{2}S_{1/2}^{{}^{\circ}}$ — 7*s*′ ^4^P_½_
7535.79	400	13266.36	5*p* ${\;}^{4}P_{3/2}^{{}^{\circ}}$ — 7*s* ^4^P_3/2_
7545.60	1	13249.11	5*p*″ ${\;}^{2}P_{1/2}^{{}^{\circ}}$ — 6*d*′ ^2^P_3/2_
7551.40 7551.48 7551.60	$\begin{array}{l} \;\\ \;\\ \; \end{array}\}\begin{array}{c} 100\\ 125\\ 175 \end{array}$	13238.93 13238.79 13238.58	$\begin{array}{l} \;\\ \;\\ \; \end{array}\}\;\left({\;}^{1}D_{2}\right)5s{\;}^{2}D_{3/2}—6p\prime {\;}^{4}D_{1/2}^{{}^{\circ}}$
7559.99	70	13223.89	5*p*′ ${\;}^{2}D_{1/2}^{{}^{\circ}}$ — 7*s*″ ^2^P_½_
7569.08	500	13208.01	5*p*′ ${\;}^{2}S_{1/2}^{{}^{\circ}}$ — 7*s*′ ^2^P_3/2_
7570.87	550	13204.89	5*p* ${\;}^{4}D_{3/2}^{{}^{\circ}}$ — 6*d* ^4^D_5/2_
7575.23	15*w*	13197.29	
7582.56	10*h*	13184.53	
7582.98	100*h*	13183.80	4*d* ^4^D_7/2_ — 4*f*″ ${\left[3\right]}_{7/2}^{{}^{\circ}}$
7586.60	25	13177.51	4*d* ^4^D_7/2_ — 6*f* ${\left[2\right]}_{5/2}^{{}^{\circ}}$
7591.61	1600	13168.81	5*s* ^4^P_3/2_ — 5*p*′ ${\;}^{2}S_{1/2}^{{}^{\circ}}$
7594.52	25	13163.77	4*d* ^4^D_7/2_ — 6*f* ${\left[4\right]}_{7/2}^{{}^{\circ}}$
7595.07	1800	13162.81	4*d* ^4^D_7/2_ — 6*f* ${\left[4\right]}_{9/2}^{{}^{\circ}}$
7598.00	4	13157.74	5*p*″ ${\;}^{2}P_{3/2}^{{}^{\circ}}$ — 8*d* ^4^F_3/2_
7604.03	70	13147.30	5*p*′ ${\;}^{2}D_{3/2}^{{}^{\circ}}$ — 7*d* ^4^P_1/2_
7606.28	120	13143.41	4*d* ^4^D_7/2_ — 6*f* ${\left[3\right]}_{7/2,\;5/2}^{{}^{\circ}}$
7607.15 7607.25 7607.38 7607.54	$\begin{array}{l} \;\\ \;\\ \;\\ \; \end{array}\}\begin{array}{c} 100\\ 150\\ 200\\ 300 \end{array}$	13141.91 13141.74 13141.51 13141.24	$\begin{array}{l} \;\\ \;\\ \; \end{array}\}\;\left({\;}^{1}D_{2}\right)5s{\;}^{2}D_{5/2}—6p\prime {\;}^{4}S_{3/2}^{{}^{\circ}}$
7616.41	2000	13125.93	5*p* ${\;}^{4}P_{3/2}^{{}^{\circ}}$ — 7*s* ^4^P_5/2_
7617.82 7617.90 7618.03	$\begin{array}{l} \;\\ \;\\ \; \end{array}\}\begin{array}{c} 3\\ 5\\ 10 \end{array}$	13123.50 13123.36 13123.14	$\begin{array}{l} \;\\ \;\\ \; \end{array}\}\;\left({\;}^{1}D_{2}\right)5s{\;}^{2}D_{5/2}—6p\prime {\;}^{4}S_{3/2}^{{}^{\circ}}$
7632.87	50	13097.63	5*p* ${\;}^{4}P_{5/2}^{{}^{\circ}}$ — 5*d* ^4^F_3/2_
7641.64	600	13082.59	5*p* ${\;}^{4}P_{5/2}^{{}^{\circ}}$ — 5*d* ^4^F_5/2_
7647.70	40	13072.23	5*p*′ ${\;}^{4}D_{1/2}^{{}^{\circ}}$ — (^1^S_0_) 5*s* ^2^S_½_
7652.60 7652.70 7652.85 7653.02	$\begin{array}{l} \;\\ \;\\ \;\\ \; \end{array}\}\begin{array}{c} 30\\ 40\\ 60\\ 120 \end{array}$	13063.86 13063.69 13063.43 13063.14	$\begin{array}{l} \;\\ \;\\ \; \end{array}\}\;\left({\;}^{1}D_{2}\right)5s{\;}^{2}D_{5/2}—6p\prime {\;}^{2}D_{3/2}^{{}^{\circ}}$
7663.39 7663.50 7663.63	$\begin{array}{l} \;\\ \;\\ \; \end{array}\}\begin{array}{c} 40\\ 50\\ 80 \end{array}$	13045.46 13045.28 13045.06	$\begin{array}{l} \;\\ \;\\ \; \end{array}\}\;\left({\;}^{1}D_{2}\right)5s{\;}^{2}D_{3/2}—6p\prime {\;}^{2}D_{3}^{{}^{\circ}}$
7680.56	5*h*	13016.30	4*d* ^4^D_1/2_ — 7*f* ${\left[1\right]}_{3/2}^{{}^{\circ}}$
7680.74	10*h*	13016.00	4*d* ^4^D_1/2_ — 7*f* ${\left[1\right]}_{1/2}^{{}^{\circ}}$
7681.75	20	13014.29	5*p*′ ${\;}^{2}D_{3/2}^{{}^{\circ}}$ — 7*d* ^4^D_½_
7685.11	50	13008.60	4*d* ^4^D_1/2_ — 7*f* ${\left[2\right]}_{3/2}^{{}^{\circ}}$
7698.72	35	12985.60	5*p*″ ${\;}^{2}P_{1/2}^{{}^{\circ}}$ — 8*d* ^4^F_3/2_
7699.58	80	12984.15	5*s*′ ^4^P_1/2_ — 5*p*″ ${\;}^{2}P_{1/2}^{{}^{\circ}}$
7703.17	2	12978.10	4*d* ^4^D_3/2_ — 9*p* ${\;}^{4}P_{1/2}^{{}^{\circ}}$
7704.32	60*h*	12976.16	4*d* ^4^F_9/2_ — 8*f* ${\left[5\right]}_{1\overset{{}^{\circ}}{1}/2}$
7706.69	15*hw*	12972.17	4*d* ^4^D_5/2_ — 6*f* ${\left[1\right]}_{3/2}^{{}^{\circ}}$
7708.39	8	12969.31	4*d* ^4^F_9/2_ — 8*f* ${\left[4\right]}_{9/2}^{{}^{\circ}}$
7709.35	15*h*	12967.69	4*d* ^4^D_5/2_ — 4*f*″ ${\left[3\right]}_{5/2}^{{}^{\circ}}$
7709.55	15*h*	12967.36	4*d* ^4^D_5/2_ — 4*f*″ ${\left[3\right]}_{7/2}^{{}^{\circ}}$
7711.68	5	12963.78	4*d* ^4^D_3/2_ — 9*p* ${\;}^{4}P_{3/2}^{{}^{\circ}}$
7713.27	200	12961.10	4*d* ^4^D_5/2_ — 6*f* ${\left[2\right]}_{5/2}^{{}^{\circ}}$
7713.54	45	12960.65	4*d* ^4^D_5/2_ — 6*f* ${\left[2\right]}_{3/2}^{{}^{\circ}}$
7715.06 7715.16 7715.31 7715.48	$\begin{array}{l} \;\\ \;\\ \;\\ \; \end{array}\}\begin{array}{c} 100\\ 150\\ 200\\ 400 \end{array}$	12958.10 12957.93 12957.68 12957.39	$\begin{array}{l} \;\\ \;\\ \; \end{array}\}\;\left({\;}^{1}D_{2}\right)5s{\;}^{2}D_{5/2}—6p\prime {\;}^{2}D_{5/2}^{{}^{\circ}}$
7717.18	2	12954.54	5*p*″ ${\;}^{2}P_{3/2}^{{}^{\circ}}$ — 8*d* ^4^D_1/2_
7721.45	300	12947.37	4*d* ^4^D_5/2_ — 6*f* ${\left[4\right]}_{7/2}^{{}^{\circ}}$
7726.02 7726.13 7726.26	$\begin{array}{l} \;\\ \;\\ \; \end{array}\}\begin{array}{c} 60\\ 80\\ 110 \end{array}$	12939.71 12939.53 12939.31	$\begin{array}{l} \;\\ \;\\ \; \end{array}\}\;\left({\;}^{1}D_{2}\right)5s{\;}^{2}D_{3/2}—6p\prime {\;}^{2}D_{5/2}^{{}^{\circ}}$
7729.19	1	12934.41	5*p*′ ${\;}^{4}D_{1/2}^{{}^{\circ}}$ — 7*d* ^4^D_3/2_
7733.61	900	12927.01	4*d* ^4^D_5/2_ — 6*f* ${\left[3\right]}_{7/2,\;5/2}^{{}^{\circ}}$
7734.61	25	12925.34	5*p*′ ${\;}^{2}S_{1/2}^{{}^{\circ}}$ — 5*d*″ ^2^D_3/2_
7742.05	1	12912.92	4*d* ^4^F_7/2_ — 5*f*′ ${\left[4\right]}_{9/2}^{{}^{\circ}}$
7783.44	9*w*	12844.26	4*d* ^4^F_7/2_ — 9*f* ${\left[5\right]}_{9/2}^{{}^{\circ}}$
7795.01	15	12825.19	5*p*′ ${\;}^{4}D_{1/2}^{{}^{\circ}}$ — 7*d* ^4^D_1/2_
7803.02	30000	12812.03	5*s*′ ^4^P_1/2_ — 5*p*″ ${\;}^{2}P_{3/2}^{{}^{\circ}}$
7807.52 7807.64 7807.78	$\begin{array}{l} \;\\ \;\\ \; \end{array}\}\begin{array}{c} 50\\ 60\\ 100 \end{array}$	12804.64 12804.44 12804.21	$\begin{array}{l} \;\\ \;\\ \; \end{array}\}\;\left({\;}^{1}D_{2}\right)5s{\;}^{2}D_{3/2}—6p\prime {\;}^{2}S_{1/2}^{{}^{\circ}}$
7821.10	30	12782.41	5*p*″ ${\;}^{2}P_{1/2}^{{}^{\circ}}$ — 8*d* ^4^D_1/2_
7827.23	1200	12772.40	5*p*′ ${\;}^{2}D_{5/2}^{{}^{\circ}}$ — 7*s*′ ^2^P_3/2_
7835.08	30	12759.60	5*p*′ ${\;}^{4}S_{3/2}^{{}^{\circ}}$ — (^1^S_0_) 5*s* ^2^S_1/2_
7841.87	30	12748.55	5*p*′ ${\;}^{4}S_{3/2}^{{}^{\circ}}$ — 7*d* ^4^F_3/2_
7843.58	600	12745.77	5*p* ${\;}^{4}P_{3/2}^{{}^{\circ}}$ — 5*d* ^4^F_5/2_
7844.01 7844.10	$\begin{array}{l} \;\\ \; \end{array}\}\begin{array}{c} 80\\ 80 \end{array}$	12745.07 12744.93	$\begin{array}{l} \;\\ \;\\ \; \end{array}\}\;5p{\;}^{4}P_{3/2}^{{}^{\circ}}—5d{\;}^{4}P_{1/2}$
7869.53	10	12703.74	4*d* ^4^F_9/2_ — 10*p* ${\;}^{4}D_{7/2}^{{}^{\circ}}$
7881.45 7881.57	$\begin{array}{l} \;\\ \; \end{array}\}\begin{array}{c} 2500s\\ 2500 \end{array}$	12684.53 12684.34	$\begin{array}{l} \;\\ \;\\ \; \end{array}\}\;5s\prime {\;}^{2}P_{3/2}—5p\prime \prime {\;}^{2}P_{1/2}^{{}^{\circ}}$
7889.85	600	12671.03	5*p*′ ${\;}^{2}D_{3/2}^{{}^{\circ}}$ — 7*s*′ ^4^P_1/2_
7903.66	50	12648.89	5*p*″ ${\;}^{2}P_{1/2}^{{}^{\circ}}$ — (^1^D_2_) 4*d* ^2^D_3/2_
7905.70	50	12645.62	5*p*′ ${\;}^{4}S_{3/2}^{{}^{\circ}}$ — 7*d* ^4^P_1/2_
7925.81	2500	12613.54	5*p* ${\;}^{4}D_{7/2}^{{}^{\circ}}$ — 7*s* ^4^P_5/2_
7929.69	250	12607.37	5*p*′ ${\;}^{2}D_{3/2}^{{}^{\circ}}$ — 7*s*′ ^2^P_3/2_
7932.99	30*W*	12602.12	$\left\{ \begin{array}{l} 4d{\;}^{4}D_{3/2}—6f\;{\left[1\right]}_{3/2}^{{}^{\circ}}\\ 4d{\;}^{4}D_{3/2}—6f\;{\left[1\right]}_{1/2}^{{}^{\circ}} \end{array}\right.$
7938.68	30000*c*	12593.09	(^1^D_2_)5*s* ^2^D_5/2_ — (^1^D_2_) 5*p* ${\;}^{4}D_{5/2}^{{}^{\circ}}$
7940.07	60	12590.88	4*d* ^4^D_3/2_ — 6*f* ${\left[2\right]}_{3/2}^{{}^{\circ}}$
7947.94	3000	12578.42	5*p* ${\;}^{4}D_{5/2}^{{}^{\circ}}$ — 7*s* ^4^P_3/2_
7950.18	3000	12574.87	(^1^D_2_)5*s* ^2^D_3/2_ — (^1^D_2_) 5*p* ${\;}^{2}D_{5/2}^{{}^{\circ}}$
7961.33	350	12557.26	4*d* ^4^D_3/2_ — 6*f* ${\left[3\right]}_{5/2}^{{}^{\circ}}$
7966.84 7967.03	$\begin{array}{l} \;\\ \; \end{array}\}\begin{array}{c} 600\\ 750 \end{array}$	12548.58 12548.28	$\begin{array}{l} \;\\ \;\\ \; \end{array}\}\;\left({\;}^{1}D_{2}\right)5s{\;}^{2}D_{5/2}—\left({\;}^{1}D_{2}\right)5p{\;}^{2}D_{3/2}^{{}^{\circ}}$
7975.11 7975.23 7975.39 7975.62	$\begin{array}{l} \;\\ \;\\ \;\\ \; \end{array}\}\begin{array}{c} 10\\ 20\\ 15\\ 35 \end{array}$	12535.56 12535.38 12535.12 12534.76	$\begin{array}{l} \;\\ \;\\ \; \end{array}\}\;\left({\;}^{1}D_{2}\right)5s{\;}^{2}D_{5/2}—4f\;{\left[1\right]}_{3/2}^{{}^{\circ}}$
7978.44 7978.57	$\begin{array}{l} \;\\ \; \end{array}\}\begin{array}{c} 8000\\ 10000 \end{array}$	12530.33 12530.13	$\begin{array}{l} \;\\ \;\\ \; \end{array}\}\;\left({\;}^{1}D_{2}\right)5s{\;}^{2}D_{3/2}—\left({\;}^{1}D_{2}\right)5p{\;}^{2}D_{3/2}^{{}^{\circ}}$
7989.94	30000	12512.30	5*s*′ ^2^P_3/2_ — 5*p*″ ${\;}^{2}P_{3/2}^{{}^{\circ}}$
7997.02	80	12501.22	4*d* ^4^D_7/2_ — 4*f*′ ${\left[3\right]}_{7/2}^{{}^{\circ}}$
7998.75	175	12498.52	5*p*′ ${\;}^{2}D_{3/2}^{{}^{\circ}}$ — 5*d*″ ^2^D_5/2_
8004.36	25	12489.76	5*p*′ ${\;}^{2}D_{5/2}^{{}^{\circ}}$ — 5*d*″ ^2^D_3/2_
8008.87	30*h*	12482.72	4*d* ^4^F_7/2_ — 8*f* ${\left[5\right]}_{9/2}^{{}^{\circ}}$
8009.36	75	12481.96	5*p*′ ${\;}^{2}D_{1/2}^{{}^{\circ}}$ — 7*s*′ ^4^P_1/2_
8009.77 8009.86 8010.00 8010.17	$\begin{array}{l} \;\\ \;\\ \;\\ \; \end{array}\}\begin{array}{c} 100\\ 150\\ 200\\ 400 \end{array}$	12481.32 12481.18 12480.96 12480.70	$\begin{array}{l} \;\\ \;\\ \; \end{array}\}\;\left({\;}^{1}D_{2}\right)5s{\;}^{2}D_{5/2}—4f\;{\left[2\right]}_{5/2}^{{}^{\circ}}$
8013.03	1	12476.24	4*d* ^4^F_7/2_ — 8*f* ${\left[4\right]}_{7/2}^{{}^{\circ}}$
8014.51 8014.60 8014.74 8014.89	$\begin{array}{l} \;\\ \;\\ \;\\ \; \end{array}\}\begin{array}{c} 100\\ 150\\ 175\\ 200 \end{array}$	12473.94 12473.80 12473.58 12473.35	$\begin{array}{l} \;\\ \;\\ \; \end{array}\}\;\left({\;}^{1}D_{2}\right)5s{\;}^{2}D_{5/2}—4f\;{\left[2\right]}_{3/2}^{{}^{\circ}}$
8021.57 8021.67 8021.80	$\begin{array}{l} \;\\ \;\\ \; \end{array}\}\begin{array}{c} 100\\ 150\\ 200 \end{array}$	12462.96 12462.80 12462.60	$\begin{array}{l} \;\\ \;\\ \; \end{array}\}\;\left({\;}^{1}D_{2}\right)5s{\;}^{2}D_{3/2}—4f\;{\left[2\right]}_{5/2}^{{}^{\circ}}$
8022.52	800	12461.48	$\left\{ \begin{array}{l} 5p{\;}^{4}P_{1/2}^{{}^{\circ}}—7s{\;}^{4}P_{3/2}\\ 5p\prime {\;}^{2}S_{1/2}^{{}^{\circ}}—5d\prime {\;}^{4}P_{3/2} \end{array}\right.$
8022.83 8022.98 8023.14	$\begin{array}{l} \;\\ \;\\ \; \end{array}\}\begin{array}{c} 15\\ 15\\ 20 \end{array}$	12461.00 12460.77 12460.52	$\begin{array}{l} \;\\ \;\\ \; \end{array}\}\;\left({\;}^{1}D_{2}\right)5s{\;}^{2}D_{5/2}—4f\;{\left[3\right]}_{7/2}^{{}^{\circ}}$
8023.91	900	12459.33	5*p* ${\;}^{4}P_{5/2}^{{}^{\circ}}$ — 5*d* ^4^D_3/2_
8026.35 8026.54	$\begin{array}{l} \;\\ \; \end{array}\}\begin{array}{c} 2000\\ 2500 \end{array}$	12455.54 12455.24	$\begin{array}{l} \;\\ \;\\ \; \end{array}\}\;\left({\;}^{1}D_{2}\right)5s{\;}^{2}D_{3/2}—4f\;{\left[2\right]}_{3/2}^{{}^{\circ}}$
8028.22	18	12452.64	4*d* ^4^D_7/2_ — 4*f*′ ${\left[4\right]}_{7/2}^{{}^{\circ}}$
8028.80	400	12451.74	4*d* ^4^D_7/2_ — 4*f*′ ${\left[4\right]}_{9/2}^{{}^{\circ}}$
8029.48 8029.53 8029.67 8029.81	$\begin{array}{l} \;\\ \;\\ \;\\ \; \end{array}\}\begin{array}{c} 100\\ 150\\ 200\\ 250 \end{array}$	12450.68 12450.60 12450.39 12450.17	$\begin{array}{l} \;\\ \;\\ \; \end{array}\}\;\left({\;}^{1}D_{2}\right)5s{\;}^{2}D_{5/2}—4f\;{\left[4\right]}_{7/2}^{{}^{\circ}}$
8030.91	250*h*	12448.47	4*d* ^4^F_9/2_ — 7*f* ${\left[5\right]}_{11/2}^{{}^{\circ}}$
8033.40	40	12444.61	4*d* ^4^D_7/2_ — 8*p* ${\;}^{4}P_{5/2}^{{}^{\circ}}$
8034.83 8034.91 8035.03	$\begin{array}{l} \;\\ \;\\ \; \end{array}\}\begin{array}{c} 75\\ 100\\ 150 \end{array}$	12442.39 12442.27 12442.08	$\begin{array}{l} \;\\ \;\\ \; \end{array}\}\;\left({\;}^{1}D_{2}\right)5s{\;}^{2}D_{3/2}—4f\;{\left[3\right]}_{5/2}^{{}^{\circ}}$
8037.34	40	12438.51	$\left\{ \begin{array}{l} 4d{\;}^{4}D_{7/2}—4f\prime \;{\left[2\right]}_{5/2}^{{}^{\circ}}\\ 4d{\;}^{4}F_{9/2}—7f\;{\left[4\right]}_{9/2}^{{}^{\circ}} \end{array}\right.$
8037.65	60	12438.03	5*p* ${\;}^{4}D_{5/2}^{{}^{\circ}}$—7s ^4^P_5/2_
8050.41	15	12418.31	5*p*′ ${\;}^{4}D_{1/2}^{{}^{\circ}}$—7*s*′ ^2^P_3/2_
8066.38	200	12393.73	5*p*″ ${\;}^{2}P_{3/2}^{{}^{\circ}}$—7*s*″ ^2^P_1/2_
8072.89	300	12383.73	5*p*′ ${\;}^{2}S_{1/2}^{{}^{\circ}}$—5*d*′ ^2^P_3/2_
8101.21	150	12340.44	5*p*′ ${\;}^{2}S_{1/2}^{{}^{\circ}}$—6*d* ^4^F_3/2_
8111.56	50	12324.70	5*p*′ ${\;}^{2}D_{3/2}^{{}^{\circ}}$—5*d*" ^2^D_3/2_
8113.04	20*h*	12322.45	4*d* ^4^F_5/2_—5*f*′ ${\left[4\right]}_{7/2}^{{}^{\circ}}$
8131.52	30000	12294.44	5*s*′ ^4^P_1/2_—5*p*′ ${\;}^{4}S_{3/2}^{{}^{\circ}}$
8137.89	150	12284.82	4*d* ^4^D_5/2_—4*f*′ ${\left[3\right]}_{7/2}^{{}^{\circ}}$
8142.80	40*h*	12277.41	$\left\{ \begin{array}{l} 4d{\;}^{4}D_{5/2}—8p{\;}^{4}D_{7/2}^{{}^{\circ}}\\ 5p\prime {\;}^{2}D_{5/2}^{{}^{\circ}}—8s{\;}^{4}P_{5/2} \end{array}\right.$
8148.84	20	12268.31	4*d* ^4^D_5/2_—8*p* ${\;}^{4}P_{3/2}^{{}^{\circ}}$
8152.65	1000*c*	12262.58	5*p* ${\;}^{4}P_{3/2}^{{}^{\circ}}$—5d ^4^D_1/2_
8153.75	10000	12260.92	5*p* ${\;}^{4}P_{5/2}^{{}^{\circ}}$—5d ^4^D_5/2_
8154.00	25000	12260.55	5*s* ^4^P_5/2_— 5*p* ${\;}^{4}D_{5/2}^{{}^{\circ}}$
8166.32	175	12242.05	5*p*" ${\;}^{2}P_{3/2}^{{}^{\circ}}$—(^1^S_0_)5*s* ^2^S_1/2_
8170.21	200*d*	12236.22	4*d* ^4^D_5/2_—4*f*′ ${\left[4\right]}_{7/2}^{{}^{\circ}}$
8172.09	200	12233.41	5*p* ${\;}^{4}D_{7/2}^{{}^{\circ}}$—5d ^4^F_5/2_
8173.71	20	12230.98	5*p*" ${\;}^{2}P_{3/2}^{{}^{\circ}}$—7d ^4^F_3/2_
8175.58	25	12228.19	4*d* ^4^D_5/2_—8*p* ${\;}^{4}P_{5/2}^{{}^{\circ}}$
8179.65	40	12222.10	4*d* ^4^D_5/2_—4*f*′ ${\left[2\right]}_{5/2}^{{}^{\circ}}$
8180.01	100	12221.56	5*p*" ${\;}^{2}P_{1/2}^{{}^{\circ}}$—7*s*″ ^2^P_1/2_
8183.52	450	12216.32	5*p*′ ${\;}^{2}S_{1/2}^{{}^{\circ}}$—5d' ^2^P_1/2_
8189.93 8190.08	$\begin{array}{l} \;\\ \; \end{array}\}\begin{array}{c} 1hw\\ 1hw \end{array}$	12206.76 12206.54	$\begin{array}{l} \;\\ \;\\ \; \end{array}\}\;4d{\;}^{4}D_{1/2}—6f\;{\left[1\right]}_{3/2}^{{}^{\circ}}$
8190.28	40	12206.24	4*d* ^4^D_1/2_—6*f* ${\left[1\right]}_{1/2}^{{}^{\circ}}$
8197.73	175*h*	12195.15	4*d* ^4^D_1/2_—6*f* ${\left[2\right]}_{3/2}^{{}^{\circ}}$
8210.49	3*h*	12176.19	4*d* ^4^F_3/2_—5*f*′ ${\left[3\right]}_{5/2}^{{}^{\circ}}$
8215.12	200	12169.33	5*p*′ ${\;}^{4}S_{3/2}^{{}^{\circ}}$—7s' ^4^P_1/2_
8237.96	500	12135.59	5*p*′ ${\;}^{4}D_{1/2}^{{}^{\circ}}$—*5d*″ ^2^D_3/2_
8246.86	5000	12122.50	5*p* ${\;}^{4}P_{3/2}^{{}^{\circ}}$ — 5*d* ^4^D_3/2_
8247.97	30	12120.86	5*p*′ ${\;}^{2}D_{3/2}^{{}^{\circ}}$ — 8*s* ^4^P_3/2_
8252.38	30	12114.39	5*p*″ ${\;}^{2}P_{3/2}^{{}^{\circ}}$ — 7*d* ^4^D_5/2_
8253.87	35	12112.20	5*p*′ ${\;}^{2}D_{3/2}^{{}^{\circ}}$ — 8*s* ^4^P_5/2_
8258.32	300	12105.67	5*p*′ ${\;}^{4}S_{3/2}^{{}^{\circ}}$ — 7*s*′ ^2^P_3/2_
8264.96	15000	12095.95	5*p* ${\;}^{4}P_{5/2}^{{}^{\circ}}$ — 5*d* ^4^D_7/2_
8272.44	75000*c*	12085.01	5*s* ^4^P_5/2_ — 5*p* ${\;}^{4}D_{7/2}^{{}^{\circ}}$
8280.24	100	12073.63	5*p*′ ${\;}^{2}D_{5/2}^{{}^{\circ}}$ — 5*d′* ^4^P_5/2_
8280.75	100*w*	12072.88	5*p* ${\;}^{4}D_{5/2}^{{}^{\circ}}$ — 5*d* ^4^F_3/2_
8291.06	900	12057.87	5*p* ${\;}^{4}D_{5/2}^{{}^{\circ}}$— 5*d* ^4^F_5/2_
8293.57	100	12054.22	5*p*′ ${\;}^{2}D_{5/2}^{{}^{\circ}}$ — 5*d′* ^2^F_5/2_
8308.49	20	12032.57	4*d* ^4^F_9/2_ — 9*p* ${\;}^{4}D_{7/2}^{{}^{\circ}}$
8313.09	10	12025.92	5*p*′ ${\;}^{2}D_{5/2}^{{}^{\circ}}$ — 5*d′* ^2^P_3/2_
8334.70	20000	11994.74	5*s*′ ^2^P_3/2_ — 5*p*′ ${\;}^{4}S_{3/2}^{{}^{\circ}}$
8343.70	10000	11981.80	5*s*′ ^4^P_1/2_ — 5*p*′ ${\;}^{4}D_{1/2}^{{}^{\circ}}$
8361.71	300	11955.99	5*p* ${\;}^{4}P_{1/2}^{{}^{\circ}}$ — 5*d* ^4^F_3/2_
8362.38	100*h*	11955.03	4*d* ^4^F_7/2_ — 7*f* ${\left[5\right]}_{9/2}^{{}^{\circ}}$
8367.20	15	11948.15	5*p*′ ${\;}^{2}D_{5/2}^{{}^{\circ}}$ — 5*d′* ^2^P_3/2_
8369.00	20	11945.58	4*d* ^4^F_7/2_ — 7*f* ${\left[4\right]}_{7/2}^{{}^{\circ}}$
8372.79	150	11940.17	5*p* ${\;}^{4}P_{1/2}^{{}^{\circ}}$ — 5*d* ^4^P_1/2_
8378.65	100*w*	11931.82	5*p*′ ${\;}^{4}D_{1/2}^{{}^{\circ}}$ — 8*s* ^4^P_3/2_
8382.42	5	11926.45	4*d* ^4^D_3/2_ — 8*p* ${\;}^{4}D_{5/2}^{{}^{\circ}}$
8384.04	1200	11924.15	5*p* ${\;}^{4}P_{3/2}^{{}^{\circ}}$ — 5*d* ^4^D_5/2_
8387.99	100	11918.53	5*p* ${\;}^{4}D_{7/2}^{{}^{\circ}}$ — 5*d* ^4^F_7/2_
8389.75	300	11916.03	5*p′* ${\;}^{2}D_{5/2}^{{}^{\circ}}$ — 5*d*′ ^2^F_7/2_
8392.76	75	11911.76	4*d* ^4^D_3/2_ — 4*f′* ${\left[3\right]}_{5/2}^{{}^{\circ}}$
8397.62	50	11904.86	5*p′* ${\;}^{2}D_{5/2}^{{}^{\circ}}$ — 6*d* ^4^F_3/2_
8402.08	20	11898.55	4*d* ^4^D_3/2_ — 8*p* ${\;}^{4}P_{3/2}^{{}^{\circ}}$
8408.68	250	11889.21	5*p′* ${\;}^{2}D_{3/2}^{{}^{\circ}}$ — 5*d′* ^2^F_5/2_
8410.79	10*w*	11886.22	4*d* ^4^F_5/2_ — 8*f* ${\left[3\right]}_{7/2,\;5/2}^{{}^{\circ}}$
8411.61	5*w*	11885.06	4*d* ^4^F_5/2_ — 8*f* ${\left[4\right]}_{7/2}^{{}^{\circ}}$
8421.88	4	11870.57	
8428.77	75	11860.87	5*p′* ${\;}^{2}D_{3/2}^{{}^{\circ}}$ — 5*d′* ^4^P_3/2_
8430.51	50	11858.42	4*d* ^4^D_3/2_ — 8*p* ${\;}^{4}P_{5/2}^{{}^{\circ}}$
8434.83	150	11852.35	4*d* ^4^D_3/2_ — 4*f*′ ${\left[2\right]}_{5/2}^{{}^{\circ}}$
8435.34	75	11851.63	4*d* ^4^D_3/2_ — 4*f*′ ${\left[2\right]}_{3/2}^{{}^{\circ}}$
8441.26	100	11843.32	5*p′* ${\;}^{2}D_{5/2}^{{}^{\circ}}$ — 6*d* ^4^F_5/2_
8446.55	40000	11835.90	5*s* ^4^P_3/2_ — 5*p* ${\;}^{4}D_{3/2}^{{}^{\circ}}$
8454.25	75	11825.12	4*d* ^4^D_7/2_ — 5*f* ${\left[2\right]}_{5/2}^{{}^{\circ}}$
8467.38	300	11806.78	4*d* ^4^D_7/2_ — 5*f* ${\left[3\right]}_{7/2,\;5/2}^{{}^{\circ}}$
8470.62	100	11802.27	4*d* ^4^D_7/2_ — 5*f* ${\left[4\right]}_{7/2}^{{}^{\circ}}$
8471.51	500	1101.03	4*d* ^4^D_7/2_ — 5*f* ${\left[4\right]}_{9/2}^{{}^{\circ}}$
8477.45	4000	11792.76	5*s′* ^4^P_1/2_ — 5*p*′ ${\;}^{2}D_{3/2}^{{}^{\circ}}$
8484.40	30	11783.10	5*s′* ${\;}^{2}D_{3/2}^{{}^{\circ}}$ — 5*d*′ ^2^P_3/2_
8503.78	400	11756.25	5*p′* ${\;}^{2}D_{5/2}^{{}^{\circ}}$ — 6*d* ^4^F_7/2_
8513.38	1500	11742.99	5*p* ${\;}^{4}D_{5/2}^{{}^{\circ}}$ — 5*d* ^4^F_7/2_
8515.69	100	11739.80	5*p*′ ${\;}^{2}D_{3/2}^{{}^{\circ}}$ — 6*d* ^4^F_3/2_
8529.38	8*h*	11720.96	
8557.73	1000	11682.13	5*s′* ^2^P_3/2_ — 5*p*′ ${\;}^{4}D_{1/2}^{{}^{\circ}}$
8560.57	300	11678.26	5*p*′ ${\;}^{2}D_{3/2}^{{}^{\circ}}$ — 6*d* ^4^F_5/2_
8565.30	200	11671.81	5*p*′ ${\;}^{4}D_{1/2}^{{}^{\circ}}$ — 5*d′* ^4^P_3/2_
8566.28	1000	11670.47	5*p* ${\;}^{4}D_{7/2}^{{}^{\circ}}$ — 5*d* ^4^F_9/2_
8578.84	400	11653.39	5*p*′ ${\;}^{2}S_{1/2}^{{}^{\circ}}$ — 6*d* ^4^D_1/2_
8580.00	20	11651.81	5*p*″ ${\;}^{2}P_{3/2}^{{}^{\circ}}$ — 7*s′* ^4^P_1/2_
8592.30	250	11635.13	4*d* ^4^F_9/2_ — 6*f* ${\left[5\right]}_{11/2}^{{}^{\circ}}$
8595.50	50	11630.80	
8595.67	50	11630.57	4*d* ^4^D_5/2_ — 5*f* ${\left[1\right]}_{3/2}^{{}^{\circ}}$
8603.00	2	11620.66	4*d* ^4^F_9/2_ — 6*f* ${\left[4\right]}_{7/2}^{{}^{\circ}}$
8603.69	100	11619.73	4*d* ^4^F_9/2_ — 6*f* ${\left[4\right]}_{9/2}^{{}^{\circ}}$
8604.06	20*hw*	11619.23	5*p*′ ${\;}^{4}S_{3/2}^{{}^{\circ}}$ — 8*s* ^4^P_3/2_
8606.68	20	11615.69	5*p*′ ${\;}^{2}D_{3/2}^{{}^{\circ}}$ — 5*d*′ ^1^P_1/2_
8608.98	30*hw*	11612.59	$\left\{ \begin{array}{l} 4d{\;}^{4}P_{1/2}—7f\;{\left[1\right]}_{3/2}^{{}^{\circ}}\\ 4d{\;}^{4}P_{1/2}—7f\;{\left[1\right]}_{1/2}^{{}^{\circ}} \end{array}\right.$
8610.49	100	11610.55	5*p*′ ${\;}^{4}S_{3/2}^{{}^{\circ}}$ — 8*s* ^4^P_5/2_
8611.85	250	11608.72	4*d* ^4^D_5/2_ — 5*f* ${\left[2\right]}_{5/2}^{{}^{\circ}}$
8612.48	75	11607.87	4*d* ^4^D_5/2_ — 5*f* ${\left[2\right]}_{3/2}^{{}^{\circ}}$
8625.38	750*c*	11590.51	(^1^D_2_)5*s* ^2^D_3/2_ — (^1^D_2_) 5*p* ${\;}^{2}P_{1/2}^{{}^{\circ}}$
8625.47	100	11590.39	4*d* ^4^D_5/2_ — 5*f* ${\left[3\right]}_{7/2,\;5/2}^{{}^{\circ}}$
8628.84	200	11585.86	4*d* ^4^D_5/2_ — 5*f* ${\left[4\right]}_{7/2}^{{}^{\circ}}$
8638.66	20000	11572.69	5*s* ^4^P_5/2_ — 5*p* ${\;}^{4}P_{3/2}^{{}^{\circ}}$
8655.11	100	11550.70	5*p*′ ${\;}^{4}D_{1/2}^{{}^{\circ}}$ — 6*d* ^4^F_3/2_
8658.84	10	11545.72	4*d* ^4^F_7/2_ — 9*p* ${\;}^{4}D_{5/2}^{{}^{\circ}}$
8668.84	300	11532.40	5*p* ${\;}^{4}D_{3/2}^{{}^{\circ}}$ — 7*s* ^4^P_3/2_
8698.53	4000	11493.04	5*s′* ^2^P_3/2_ — 5*p′* ${\;}^{2}D_{3/2}^{{}^{\circ}}$
8708.68	75	11479.64	5*p″* ${\;}^{2}P_{1/2}^{{}^{\circ}}$ — 7*s′* ^4^P_1/2_
8708.96	100	11479.27	5*p″* ${\;}^{2}P_{3/2}^{{}^{\circ}}$ — 5*d*″ ^4^D_5/2_
8725.33	500	11457.74	5*p* ${\;}^{4}P_{1/2}^{{}^{\circ}}$ — 5*d* ^4^D_1/2_
8726.75	100*c*	11455.87	4*d* ^4^D_1/2_ — 4*f′* ${\left[2\right]}_{3/2}^{{}^{\circ}}$
8741.64	50	11436.36	5*p*′ ${\;}^{2}D_{5/2}^{{}^{\circ}}$ — 6*d* ^4^D_5/2_
8742.99	75	11434.59	5*p* ${\;}^{4}D_{5/2}^{{}^{\circ}}$ — 5*d* ^4^D_3/2_
8749.10	10	11426.61	5*p*′ ${\;}^{4}D_{1/2}^{{}^{\circ}}$ — 5*d*′ ^2^P_1/2_
8757.24	10	11415.99	5*p*″ ${\;}^{2}P_{1/2}^{{}^{\circ}}$ — 7*s*′ ^2^P_3/2_
8760.46	700	11411.79	5*p* ${\;}^{4}D_{7/2}^{{}^{\circ}}$ — 5*d* ^4^D_5/2_
8764.20	500	11406.92	5*p*′ ${\;}^{4}S_{3/2}^{{}^{\circ}}$ — 5*d*′ ^4^P_5/2_
8775.71	250	11391.96	5*p* ${\;}^{4}D_{3/2}^{{}^{\circ}}$ — 7*s* ^4^P_5/2_
8779.15	50	11387.50	5*p*′ ${\;}^{4}S_{3/2}^{{}^{\circ}}$ — 5*d*′ ^2^F_5/2_
8793.47	10000*c*	11368.95	(^1^D_2_)5*s* ^2^D_5/2_ — (^1^D_2_) 5*p* ${\;}^{2}P_{3/2}^{{}^{\circ}}$
8798.04	6	11363.05	4*d* ^4^F_5/2_ — 7*f* ${\left[2\right]}_{5/2}^{{}^{\circ}}$
8801.03	150	11359.19	5*p*′ ${\;}^{4}S_{3/2}^{{}^{\circ}}$ — 5*d*′ ^4^P_3/2_
8803.41	100	11356.12	$\left\{ \begin{array}{l} 4d{\;}^{4}F_{5/2}—7f\;{\left[3\right]}_{7/2,\;5/2}^{{}^{\circ}}\\ 5p\prime {\;}^{2}D_{5/2}^{{}^{\circ}}—6d{\;}^{4}D_{3/2} \end{array}\right.$
8804.76	20	11354.38	4*d* ^4^F_5/2_ — 7*f* ${\left[4\right]}_{7/2}^{{}^{\circ}}$
8807.44 8807.68	$\begin{array}{l} \;\\ \; \end{array}\}\begin{array}{c} 600\\ 800 \end{array}$	11350.92 11350.61	$\begin{array}{l} \;\\ \;\\ \; \end{array}\}\;\left({\;}^{1}D_{2}\right)5s{\;}^{2}D_{3/2}—\left({\;}^{1}D_{2}\right)5p{\;}^{2}P_{3/2}^{{}^{\circ}}$
8808.85	500	11349.10	5*p*′ ${\;}^{2}D_{5/2}^{{}^{\circ}}$ — 6*d* ^4^D_7/2_
8819.96	15000	11334.81	(^1^D_2_)5*s* ^2^D_5/2_ — (^1^D_2_) 5*p* ${\;}^{2}F_{7/2}^{{}^{\circ}}$
8825.22	25000	11328.05	5*s*′ ^2^P_3/2_ — 5*p′* ${\;}^{2}D_{5/2}^{{}^{\circ}}$
8833.33	175	11317.65	5*p* ${\;}^{4}P_{1/2}^{{}^{\circ}}$ — 5*d* ^4^D_3/2_
8842.85	125	11305.47	5*p*″ ${\;}^{2}P_{3/2}^{{}^{\circ}}$ — 5*d″* ^2^D_3/2_
8861.69	85	11281.43	5*p*′ ${\;}^{4}S_{3/2}^{{}^{\circ}}$ — 5*d′* ^2^P_3/2_
8869.64	300	11271.32	5*p*′ ${\;}^{2}D_{3/2}^{{}^{\circ}}$ — 6*d* ^4^D_5/2_
8870.46	200	11270.28	5*p* ${\;}^{4}P_{3/2}^{{}^{\circ}}$ — 6*s″* ^2^P_1/2_
8875.43	3	11263.97	6*s* ^4^P_5/2_ — 7*p″* ${\;}^{2}P_{3/2}^{{}^{\circ}}$
8877.89	90	11260.85	4*d* ^4^D_3/2_ — 5*f* ${\left[1\right]}_{3/2}^{{}^{\circ}}$
8878.40	90	11260.20	4*d* ^4^D_3/2_ — 5*f* ${\left[1\right]}_{1/2}^{{}^{\circ}}$
8886.34	40	11250.14	5*p* ${\;}^{4}S_{3/2}^{{}^{\circ}}$ — 6*d* ^4^P_1/2_
8888.98	4000	11246.80	5*p*′ ${\;}^{4}D_{7/2}^{{}^{\circ}}$ — 5*d* ^4^D_7/2_
8895.21	20	11238.92	4*d* ^4^D_3/2_ — 5*f* ${\left[2\right]}_{5/2}^{{}^{\circ}}$
8895.87	150	11238.09	$\left\{ \begin{array}{l} 4d{\;}^{4}D_{3/2}—5f\;{\left[2\right]}_{3/2}^{{}^{\circ}}\\ 5p\prime {\;}^{4}S_{3/2}^{{}^{\circ}}—6d{\;}^{4}F_{3/2} \end{array}\right.$
8897.62	30000	11235.88	5*s* ^4^P_5/2_ — 5*p* ${\;}^{4}P_{5/2}^{{}^{\circ}}$
8909.73	300	11220.60	4*d* ^4^D_3/2_ — 5*f* ${\left[3\right]}_{5/2}^{{}^{\circ}}$
8925.64	15	11200.60	4*d* ^4^F_3/2_ — 7*f* ${\left[1\right]}_{3/2}^{{}^{\circ}}$
8932.40	6000	11192.13	5*s′* ^4^P_1/2_ — 5*p′* ${\;}^{2}S_{1/2}^{{}^{\circ}}$
8933.29	25	11191.01	5*p*′ ${\;}^{2}D_{3/2}^{{}^{\circ}}$ — 6*d* ^4^D_3/2_
8936.96	15	11186.42	4*d* ${\;}^{4}F_{3/2}^{{}^{\circ}}$ — 7*f* ${\left[3\right]}_{5/2}^{{}^{\circ}}$
8944.83	50	11176.57	5*p*′ ${\;}^{2}S_{3/2}^{{}^{\circ}}$ — 6*d* ^4^F_5/2_
8949.39	1800	11170.88	(^1^D_2_)5*s* ^2^D_5/2_ — (^1^D_2_) 5*p* ${\;}^{2}F_{5/2}^{{}^{\circ}}$
8964.00	9000	11152.67	(^1^D_2_)5*s* ^2^D_3/2_ — (^1^D_2_) 5*p* ${\;}^{2}F_{5/2}^{{}^{\circ}}$
8972.83	350	11141.70	4*d* ^4^F_7/2_ — 6*f* ${\left[5\right]}_{9/2}^{{}^{\circ}}$
8979.59	200	11133.31	5*p*″ ${\;}^{2}P_{1/2}^{{}^{\circ}}$ — 5*d″* ^4^D_3/2_
8984.56	100	11127.15	4*d* ^4^F_7/2_ — 6*f* ${\left[4\right]}_{7/2}^{{}^{\circ}}$
8985.32	1	11126.21	4*d* ^4^F_7/2_ — 6*f* ${\left[4\right]}_{9/2}^{{}^{\circ}}$
8995.21	50	11113.98	5*p*′ ${\;}^{4}S_{3/2}^{{}^{\circ}}$ — 5*d′* ^2^P_1/2_
9000.99	2*h*	11106.84	4*d* ^4^F_7/2_ — 6*f* ${\left[3\right]}_{7/2,\;5/2}^{{}^{\circ}}$
9005.20	50*h*	11101.65	5*p*″ ${\;}^{2}P_{3/2}^{{}^{\circ}}$ — 8*s* ^4^P_3/2_
9012.23	20	11092.99	5*p*″ ${\;}^{2}P_{3/2}^{{}^{\circ}}$ — 8*s* ^4^P_5/2_
9029.94	300	11071.23	5*p* ${\;}^{4}D_{5/2}^{{}^{\circ}}$ — 5*d* ^4^D_7/2_
9045.06	40	11052.72	5*p′* ${\;}^{2}D_{3/2}^{{}^{\circ}}$ — 6*d* ^4^D_1/2_
9066.28	400	11026.86	5*p* ${\;}^{4}D_{3/2}^{{}^{\circ}}$ — 5*d* ^4^F_3/2_
9078.64	700	11011.84	5*p* ${\;}^{4}D_{3/2}^{{}^{\circ}}$ — 5*d* ^4^F_5/2_
9079.29	125	11011.05	5*p* ${\;}^{4}D_{3/2}^{{}^{\circ}}$ — 5*d* ^4^P_1/2_
9084.29	25	11004.99	4*d* ^4^F_5/2_ — 9*p* ${\;}^{4}D_{3/2}^{{}^{\circ}}$
9086.80	100	11001.95	5*p′* ${\;}^{4}D_{1/2}^{{}^{\circ}}$ — 6*d* ^4^D_3/2_
9107.37	10	10977.11	4*d′* ^4^F_5/2_ — 5*f″* ${\left[3\right]}_{7/2}^{{}^{\circ}}$
9123.17	50	10958.09	4*d* ^4^F_9/2_ — 4*f′* ${\left[3\right]}_{7/2}^{{}^{\circ}}$
9126.14	4	10954.53	4*d* ^4^F_5/2_ — 9*p* ${\;}^{4}D_{5/2}^{{}^{\circ}}$
9129.31	70	10950.72	4*d* ^4^F_9/2_ — 8*p* ${\;}^{4}D_{7/2}^{{}^{\circ}}$
9147.04	75*h*	10929.50	5*p*″ ${\;}^{2}P_{1/2}^{{}^{\circ}}$ — 8*s* ^4^P_3/2_
9166.06	30000	10906.82	5*s* ^4^P_3/2_ — 8*s* ${\;}^{4}P_{1/2}^{{}^{\circ}}$
9173.63	15000	10897.82	5*s*″ ^2^P_1/2_ — 5*p″* ${\;}^{2}P_{1/2}^{{}^{\circ}}$
9178.16	20000	10892.44	5*s*′ ^2^P_3/2_ — 5*p*′ ${\;}^{2}S_{1/2}^{{}^{\circ}}$
9183.14	30	10886.53	$\left\{ \begin{array}{l} 4d{\;}^{4}F_{3/2}—7p\prime {\;}^{2}S_{1/2}^{{}^{\circ}}\\ 4d\prime {\;}^{2}P_{1/2}—5f\prime \;{\left[2\right]}_{3/2}^{{}^{\circ}} \end{array}\right.$
9197.16	50	10869.94	5*p*″ ${\;}^{2}P_{3/2}^{{}^{\circ}}$ — 5*d′* ^4^F_5/2_
9201.21	80*w*	10865.15	4*d* ^4^D_1/2_ — 5*f* ${\left[1\right]}_{3/2}^{{}^{\circ}}$
9201.78	80	10864.48	4*d* ^4^D_1/2_ — 5*f* ${\left[1\right]}_{1/2}^{{}^{\circ}}$
9202.47	80	10863.67	5*p*′ ${\;}^{4}D_{1/2}^{{}^{\circ}}$ — 6*d* ^4^D_1/2_
9220.56	200	10842.35	4*d* ^4^D_1/2_ — 5*f* ${\left[2\right]}_{3/2}^{{}^{\circ}}$
9221.19	35	10841.61	5*p*″ ${\;}^{2}P_{3/2}^{{}^{\circ}}$ — 5*d′* ^4^P_3/2_
9226.57	15	10835.29	4*d* ^4^F_3/2_ — 9*p*′ ${\;}^{4}D_{3/2}^{{}^{\circ}}$
9254.06	70	10803.10	4*d* ^4^P_1/2_ — 6*f* ${\left[1\right]}_{3/2}^{{}^{\circ}}$
9254.39	30	10802.72	4*d* ^4^P_1/2_ — 6*f* ${\left[1\right]}_{1/2}^{{}^{\circ}}$
9265.42	40000	10789.86	5*s* ^4^P_3/2_ — 5*p* ${\;}^{4}D_{5/2}^{{}^{\circ}}$
9282.80	100	10769.66	5*p*′ ${\;}^{2}S_{3/2}^{{}^{\circ}}$ — 6*d* ^4^D_5/2_
9287.82	2	10763.84	5*p*″ ${\;}^{2}P_{3/2}^{{}^{\circ}}$ — 5*d′* ^2^P_3/2_
9288.52	200	10763.02	5*p* ${\;}^{4}P_{5/2}^{{}^{\circ}}$ — 6*s′* ^2^P_3/2_
9314.85	30	10732.60	5*p*″ ${\;}^{2}P_{3/2}^{{}^{\circ}}$ — 6*d′* ^4^P_1/2_
9320.86	15000	10725.68	5*s*″ ^2^P_1/2_ — 5*p″* ${\;}^{2}P_{3/2}^{{}^{\circ}}$
9325.32	20	10720.55	5*p*″ ${\;}^{2}P_{3/2}^{{}^{\circ}}$ — 6*d* ^4^F_3/2_
9352.55	175	10689.34	5*p*′ ${\;}^{4}S_{3/2}^{{}^{\circ}}$ — 6*d* ^4^D_3/2_
9354.56	15	10687.04	4*d* ^4^D_5/2_ — 6*p″* ${\;}^{2}P_{3/2}^{{}^{\circ}}$
9369.94	100	10669.50	5*p*″ ${\;}^{2}P_{1/2}^{{}^{\circ}}$ — 5*d′* ^4^P_3/2_
9378.84	1	10659.37	4*d′* ^2^F_7/2_ — 5*f′* ${\left[3\right]}_{5/2}^{{}^{\circ}}$
9379.21	30	10658.95	4*d′* ^2^F_7/2_ — 5*f′* ${\left[3\right]}_{7/2}^{{}^{\circ}}$
9399.55	5	10635.89	4*d′* ^2^F_7/2_ — 5*f′* ${\left[4\right]}_{7/2}^{{}^{\circ}}$
9400.19	90	10635.17	4*d′* ^2^F_7/2_ — 5*f′* ${\left[4\right]}_{9/2}^{{}^{\circ}}$
9409.56	20	10624.57	4*d* ^4^D_7/2_ — 7*p* ${\;}^{4}D_{5/2}^{{}^{\circ}}$
9428.58	3	10603.14	6*s* ^4^P_5/2_ — 7*p′* ${\;}^{4}S_{3/2}^{{}^{\circ}}$
9434.51	3	10596.48	5*p*″ ${\;}^{2}P_{3/2}^{{}^{\circ}}$ — 5*d′* ^2^P_1/2_
9438.20	80	10592.33	4*d* ^4^D_7/2_ — 7*p* ${\;}^{4}D_{7/2}^{{}^{\circ}}$
9460.14	500*d*	10567.77	5*p* ${\;}^{4}P_{3/2}^{{}^{\circ}}$ — 6*s′* ^4^P_1/2_
9466.45	2*w*	10560.73	4*d* ^4^F_5/2_ — 6*f* ${\left[1\right]}_{3/2}^{{}^{\circ}}$
9470.67	20*h*	10556.02	4*d* ^4^F_5/2_ — 4*f″* ${\left[3\right]}_{7/2}^{{}^{\circ}}$
9475.11	80	10551.07	5*p*′ ${\;}^{4}S_{3/2}^{{}^{\circ}}$ — 6*d* ^4^D_1/2_
9476.32	30	10549.73	4*d* ^4^F_5/2_ — 6*f* ${\left[2\right]}_{5/2}^{{}^{\circ}}$
9477.50	60	10548.41	5*p*″ ${\;}^{2}P_{1/2}^{{}^{\circ}}$ — 6*d* ^4^F_3/2_
9488.66	120	10536.01	4*d* ^4^F_5/2_ — 6*f* ${\left[4\right]}_{7/2}^{{}^{\circ}}$
9495.30	50	10528.64	5*p* ${\;}^{4}D_{3/2}^{{}^{\circ}}$ — 5*d* ^4^D_1/2_
9498.32	20	10525.29	4*d* ^4^D_5/2_ — 5*d* ${\;}^{4}D_{3/2}^{{}^{\circ}}$
9503.78	6*w*	10519.24	5*p* ${\;}^{4}P_{5/2}^{{}^{\circ}}$ — 4*d″* ^2^D_3/2_
9507.03	75	10515.65	4*d* ^4^F_5/2_ — 6*f* ${\left[3\right]}_{7/2,\;5/2}^{{}^{\circ}}$
9517.27	100	10504.33	4*d* ^4^D_7/2_ — 7*p* ${\;}^{4}P_{5/2}^{{}^{\circ}}$
9543.03	40	10475.98	4*d* ^4^F_7/2_ — 8*p* ${\;}^{4}D_{5/2}^{{}^{\circ}}$
9552.67	175	10465.41	5*p* ${\;}^{4}P_{1/2}^{{}^{\circ}}$ — 6*s″* ^2^P_1/2_
9556.43	2*h*	10461.29	4*d* ^4^F_7/2_ — 4*f′* ${\left[3\right]}_{5/2}^{{}^{\circ}}$
9560.17	6	10457.20	4*d* ^4^F_7/2_ — 8*p* ${\;}^{4}D_{7/2}^{{}^{\circ}}$
9576.17	1	10439.72	4*d′* ^2^P_3/2_ — 5*f′* ${\left[3\right]}_{5/2}^{{}^{\circ}}$
9588.61	300	10426.18	5*p* ${\;}^{4}P_{3/2}^{{}^{\circ}}$ — 6*s′* ^2^P_3/2_
9590.33	20	10424.31	5*p″* ${\;}^{2}P_{1/2}^{{}^{\circ}}$ — 5*d′* ^2^P_1/2_
9597.98	2	10416.00	4*d* ^2^F_7/2_ — 4*f′* ${\left[4\right]}_{7/2}^{{}^{\circ}}$
9598.79	60	10415.12	4*d* ^2^F_7/2_ — 4*f′* ${\left[4\right]}_{9/2}^{{}^{\circ}}$
9603.62	35	10409.89	4*d′* ^2^P_3/2_ — 5*f′* ${\left[2\right]}_{5/2}^{{}^{\circ}}$
9604.30	6	10409.15	4*d′* ^2^P_3/2_ — 5*f′* ${\left[2\right]}_{3/2}^{{}^{\circ}}$
9605.34	1	10408.02	4*d* ^4^F_7/2_ — 8*p* ${\;}^{4}P_{5/2}^{{}^{\circ}}$
9621.02	35*h*	10391.06	4*d* ^4^F_3/2_ — 6*f* ${\left[1\right]}_{3/2}^{{}^{\circ}}$
9623.33	15	10388.56	5*p* ${\;}^{4}D_{3/2}^{{}^{\circ}}$ — 5*d* ^4^D_3/2_
9625.17	8	10386.58	4*d* ^4^F_3/2_ — 4*f″* ${\left[3\right]}_{5/2}^{{}^{\circ}}$
9627.54	4	10384.02	6*s* ^4^P_5/2_ — 5*d* ${\;}^{4}D_{7/2}^{{}^{\circ}}$
9631.30	60	10379.97	4*d* ^4^F_3/2_ — 6*f* ${\left[2\right]}_{5/2}^{{}^{\circ}}$
9635.05	20	10375.93	4*d* ^4^D_5/2_ — 7*p* ${\;}^{4}D_{7/2}^{{}^{\circ}}$
9661.08	1	10347.97	6*s* ^4^P_5/2_ — 9*p* ${\;}^{4}P_{5/2}^{{}^{\circ}}$
9662.99	20	10345.93	4*d* ^4^F_3/2_ — 6*f* ${\left[3\right]}_{5/2}^{{}^{\circ}}$
9710.68	12	10295.12	4*d′* ^4^F_5/2_ — 5*f′* ${\left[3\right]}_{5/2}^{{}^{\circ}}$
9717.48	40	10287.91	4*d* ^4^D_5/2_ — 7*p* ${\;}^{4}P_{5/2}^{{}^{\circ}}$
9719.20	600	10286.09	4*d* ^4^F_9/2_ — 5*f* ${\left[5\right]}_{11/2}^{{}^{\circ}}$
9725.42	40	10279.51	4*d* ^4^D_5/2_ — 7*p* ${\;}^{4}P_{3/2}^{{}^{\circ}}$
9731.76	350	10272.82	5*p* ${\;}^{4}P_{5/2}^{{}^{\circ}}$ — 4*d″* ^2^D_5/2_
9732.90	50	10271.61	4*d*′ ^4^F_5/2_ — 5*f*′ ${\left[4\right]}_{7/2}^{{}^{\circ}}$
9740.41	15	10263.69	4*d* ^4^F_9/2_ — 5*f* ${\left[3\right]}_{7/2}^{{}^{\circ}}$
9745.86	100	10257.95	4*d* ^4^F_9/2_ — 5*f* ${\left[4\right]}_{9/2}^{{}^{\circ}}$
9751.45	90	10252.07	5*p″* ${\;}^{2}P_{3/2}^{{}^{\circ}}$ — 6*d* ^4^D_5/2_
9793.48	6000	10208.08	5*s″* ^2^P_1/2_ — 5*p′* ${\;}^{4}S_{3/2}^{{}^{\circ}}$
9810.67	60	10190.19	5*p* ${\;}^{4}D_{3/2}^{{}^{\circ}}$ — 5*d* ^4^D_5/2_
9818.04 9818.21	$\begin{array}{l} \;\\ \; \end{array}\}\begin{array}{c} 60\\ 70 \end{array}$	10182.54 10182.36	$\begin{array}{l} \;\\ \;\\ \; \end{array}\}\;5p{\;}^{4}P_{3/2}^{{}^{\circ}}—4d\prime \prime {\;}^{2}D_{3/2}$
9828.40	15	10171.81	5*p″* ${\;}^{2}P_{3/2}^{{}^{\circ}}$ — 6*d* ^4^D_3/2_
9831.25	2	10168.86	4*d* ^4^P_1/2_ — 8*p* ${\;}^{4}P_{1/2}^{{}^{\circ}}$
9844.17	7	10155.51	4*d* ^4^D_3/2_ — 7*p* ${\;}^{4}D_{3/2}^{{}^{\circ}}$
9864.95	2	10134.12	4*d* ^4^D_3/2_ — 7*p* ${\;}^{4}P_{1/2}^{{}^{\circ}}$
9896.40	10000	10101.91	5*s* ^4^P_3/2_ — 5*p* ${\;}^{4}P_{3/2}^{{}^{\circ}}$
9943.85 9944.18	$\begin{array}{l} \;\\ \; \end{array}\}\begin{array}{c} 250\\ 250 \end{array}$	10053.71 10053.38	$\begin{array}{l} \;\\ \;\\ \; \end{array}\}\;\left({\;}^{1}D_{2}\right)5s{\;}^{2}D_{5/2}—\left({\;}^{1}D_{2}\right)6p{\;}^{4}D_{3/2}^{{}^{\circ}}$
9945.22	1	10052.33	4*d* ^4^P_1/2_ — 4*f′* ${\left[2\right]}_{3/2}^{{}^{\circ}}$
9959.04	1	10038.38	4*d* ^4^D_3/2_ — 7*p* ${\;}^{4}D_{5/2}^{{}^{\circ}}$
9961.92 9962.13	$\begin{array}{l} \;\\ \; \end{array}\}\begin{array}{c} 20\\ 30 \end{array}$	10035.47 10035.26	$\begin{array}{l} \;\\ \;\\ \; \end{array}\}\;\left({\;}^{1}D_{2}\right)5s{\;}^{2}D_{3/2}—\left({\;}^{1}D_{2}\right)6p{\;}^{4}D_{3/2}^{{}^{\circ}}$
9963.89	1	10033.49	5*p″* ${\;}^{2}P_{3/2}^{{}^{\circ}}$ — 6*d* ^4^D_1/2_
10001.96	10	9995.30	4*d* ^4^F_5/2_ — 7*p* ${\;}^{4}D_{3/2}^{{}^{\circ}}$
10025.23	4*h*	9972.10	6*s* ^4^P_5/2_ — 6*f* ${\left[4\right]}_{7/2}^{{}^{\circ}}$
10045.71	3*h*	9951.77	6*s* ^4^P_5/2_ — 6*f* ${\left[3\right]}_{7/2,\;5/2}^{{}^{\circ}}$
10056.87	^15^	9940.73	4*d′* ^4^P_3/2_ — 5*f′* ${\left[3\right]}_{5/2}^{{}^{\circ}}$
10061.53 10061.68	$\begin{array}{l} \;\\ \; \end{array}\}\begin{array}{c} 25\\ 25 \end{array}$	9936.12 9935.97	$\begin{array}{l} \;\\ \;\\ \; \end{array}\}\;5p{\;}^{4}P_{3/2}^{{}^{\circ}}—4d\prime \prime {\;}^{2}D_{5/2}$
10079.75	10	9918.16	4*d* ^4^D_3/2_ — 7*p* ${\;}^{4}P_{5/2}^{{}^{\circ}}$
10085.99	35	9912.03	(^1^D_2_)5*s* ^2^D_3/2_ — 6*p* ${\;}^{4}D_{5/2}^{{}^{\circ}}$
10087.94	1	9910.11	4*d′* ^4^P_3/2_ — 5*f′* ${\left[2\right]}_{3/2}^{{}^{\circ}}$
10088.31	10	9909.75	4*d* ^4^D_3/2_ — 7*p* ${\;}^{4}P_{3/2}^{{}^{\circ}}$
10102.82	12	9895.51	5*s″* ^2^P_1/2_ — 5*d′* ${\;}^{4}D_{1/2}^{{}^{\circ}}$
10107.60 10107.86	$\begin{array}{l} \;\\ \; \end{array}\}\begin{array}{c} 100\\ 150 \end{array}$	9890.83 9890.58	$\begin{array}{l} \;\\ \;\\ \; \end{array}\}\;\left({\;}^{1}D_{2}\right)5s{\;}^{2}D_{3/2}—6p{\;}^{4}P_{1/2}^{{}^{\circ}}$
10113.74	2	9884.83	4*d* ^4^F_5/2_ — 8*p* ${\;}^{4}D_{5/2}^{{}^{\circ}}$
10119.19	3	9879.51	4*d* ^4^D_1/2_ — 6*p″* ${\;}^{2}P_{1/2}^{{}^{\circ}}$
10128.79	4	9870.14	4*d* ^4^F_5/2_ — 4*f′* ${\left[3\right]}_{5/2}^{{}^{\circ}}$
10133.00	5	9866.04	4*d* ^4^F_5/2_ — 8*p* ${\;}^{4}D_{7/2}^{{}^{\circ}}$
10140.08	3000	9859.15	5*s′* ^4^P_1/2_ — 5*p* ${\;}^{4}D_{3/2}^{{}^{\circ}}$
10174.75	12	9825.56	4*d* ^4^F_3/2_ — 8*p* ${\;}^{4}D_{3/2}^{{}^{\circ}}$
10175.47	20	9824.86	4*d* ^4^F_5/2_ — 4*f′* ${\left[4\right]}_{7/2}^{{}^{\circ}}$
10184.02 10184.49	$\begin{array}{l} \;\\ \; \end{array}\}\begin{array}{c} 300\\ 200 \end{array}$	9816.61 9816.16	$\begin{array}{l} \;\\ \;\\ \; \end{array}\}\;5p{\;}^{4}P_{3/2}^{{}^{\circ}}—4s4p^{6}{\;}^{2}S_{1/2}$
10197.61	18	9803.53	4*d* ^4^D_7/2_ — 6*p′* ${\;}^{2}D_{5/2}^{{}^{\circ}}$
10208.91	300	9792.68	4*d* ^4^F_7/2_ — 5*f* ${\left[5\right]}_{9/2}^{{}^{\circ}}$
10225.23	1	9777.05	4*d′* ^2^F_5/2_ — 7*p″* ${\;}^{2}P_{3/2}^{{}^{\circ}}$
10231.61	8	9770.95	4*d* ^4^D_5/2_ — 6*p′* ${\;}^{4}S_{3/2}^{{}^{\circ}}$
10232.39	15	9770.21	4*d* ^4^F_7/2_ — 5*f* ${\left[3\right]}_{7/2,\;5/2}^{{}^{\circ}}$
10237.14	75	9765.68	4*d* ^4^F_7/2_ — 5*f* ${\left[4\right]}_{7/2}^{{}^{\circ}}$
10237.74	6000	9765.10	5*s* ^4^P_3/2_ — 5*p* ${\;}^{4}P_{5/2}^{{}^{\circ}}$
10243.31	3	9759.79	4*d* ^4^D_1/2_ — 7*p* ${\;}^{4}D_{3/2}^{{}^{\circ}}$
10248.27	3	9755.07	4*d″* ^2^D_5/2_ — 5*f″* ${\left[3\right]}_{7/2}^{{}^{\circ}}$
10265.89	15*h*	9738.33	5*p* ${\;}^{4}D_{5/2}^{{}^{\circ}}$ — 6*s*′ ^2^P_3/2_
10293.23	1	9712.46	4*d′* ^4^P_5/2_ — 5*f′* ${\left[3\right]}_{5/2}^{{}^{\circ}}$
10293.65	50	9712.07	4*d′* ^4^P_5/2_ — 5*f′* ${\left[3\right]}_{7/2}^{{}^{\circ}}$
10299.62	1000	9706.44	5*s″* ^2^P_1/2_ — 5*p′* ${\;}^{2}D_{3/2}^{{}^{\circ}}$
10305.99	10	9700.44	4*d* ^4^F_3/2_ — 4*f′* ${\left[3\right]}_{5/2}^{{}^{\circ}}$
10310.62 10310.92	$\begin{array}{l} \;\\ \; \end{array}\}\begin{array}{c} 600s\\ 700 \end{array}$	9696.08 9695.80	$\begin{array}{l} \;\\ \;\\ \; \end{array}\}\;\left({\;}^{1}D_{2}\right)5s{\;}^{2}D_{5/2}—6p{\;}^{4}P_{3/2}^{{}^{\circ}}$
10312.90	40	9693.94	5*p′* ${\;}^{2}S_{1/2}^{{}^{\circ}}$ — 5*d* ^4^F_3/2_
10314.03	8	9692.87	4*d* ^4^D_5/2_ — 6*p′* ${\;}^{2}D_{3/2}^{{}^{\circ}}$
10320.09	15	967.18	4*d* ^4^F_3/2_ — 8*p* ${\;}^{4}P_{3/2}^{{}^{\circ}}$
10324.96	3	9682.61	4*d′* ^4^P_5/2_ — 5*f′* ${\left[2\right]}_{5/2}^{{}^{\circ}}$
10329.74 10330.17	$\begin{array}{l} \;\\ \; \end{array}\}\begin{array}{c} 80s\\ 100 \end{array}$	9678.13 9677.73	$\begin{array}{l} \;\\ \;\\ \; \end{array}\}\;\left({\;}^{1}D_{2}\right)5s{\;}^{2}D_{3/2}—6p{\;}^{4}P_{3/2}^{{}^{\circ}}$
10370.29	1	9640.29	4*d* ^4^F_3/2_ — 4*f′* ${\left[2\right]}_{3/2}^{{}^{\circ}}$
10374.37	12*cw*	9636.50	(^1^D_2_)5*s* ^2^D_3/2_ — 6*p* ${\;}^{4}P_{5/2}^{{}^{\circ}}$
10377.65	1500	9633.45	5*p* ${\;}^{4}P_{5/2}^{{}^{\circ}}$ — 4*d′* ^4^P_5/2_
10384.15	2	9627.42	4*d* ^4^F_3/2_ — 4*f′* ${\;}^{2}S_{1/2}^{{}^{\circ}}$
10388.40	1	9623.48	4*d* ${\;}^{2}D_{5/2}^{{}^{\circ}}$ — 7*s* ^4^P_5/2_
10390.74	175	9621.32	5*p* ${\;}^{4}P_{1/2}^{{}^{\circ}}$ — 6*s′* ^4^P_3/2_
10414.97 10415.10	$\begin{array}{l} \;\\ \; \end{array}\}\begin{array}{c} 8\\ 8 \end{array}$	$\begin{array}{l} 9598.93\\ 9598.81 \end{array}\}$	5*p′* ${\;}^{2}D_{3/2}^{{}^{\circ}}$ — 7*s* ^4^P_3/2_
10457.96	30000	9559.47	5*p* ^2^P_3/2_ — 5*p* ${\;}^{4}D_{3/2}^{{}^{\circ}}$
10483.35	8	9530.32	5*p* ${\;}^{4}D_{3/2}^{{}^{\circ}}$ — 6*s″* ^2^P_½_
10505.05	8	9516.62	4*d* ^2^D_3/2_ — 6*p′* ${\;}^{4}D_{1/2}^{{}^{\circ}}$
10507.91	12	9514.03	4*d″* ^4^D_1/2_ — 7*p* ${\;}^{4}P_{3/2}^{{}^{\circ}}$
10513.67	1	9508.82	4*d″* ^2^D_3/2_ — 5*f″* ${\left[3\right]}_{5/2}^{{}^{\circ}}$
$\begin{array}{l} \;10529.\;40\\ 10529.\;58 \end{array}\}$	3 3	9494.62 9494.45	$\begin{array}{l} \;\\ \;\\ \; \end{array}\}\;5p{\;}^{4}D_{5/2}^{{}^{\circ}}—4d\prime \prime {\;}^{2}D_{3/2}$
10554.72	1	9471.84	
10566.15	250	9461.59	4*d* ^4^P_1/2_ — 5*f* ${\left[1\right]}_{3/2}^{{}^{\circ}}$
10566.87	100	9460.95	4*d* ^4^P_1/2_ — 5*f* ${\left[1\right]}_{1/2}^{{}^{\circ}}$
10560.67	2*d*	9458.44	5*p′* ${\;}^{2}D_{3/2}^{{}^{\circ}}$ — 7*s* ^2^P_5/2_
10591.51	10*w*	9438.94	4*d* ^4^D_7/2_ — (^1^D_2_) 5*p* ${\;}^{2}D_{5/2}^{{}^{\circ}}$
10599.94	1	9431.43	6*s* ^4^P_5/2_ — 8*p* ${\;}^{4}D_{3/2}^{{}^{\circ}}$
10608.66	40	9423.68	5*p* ${\;}^{4}D_{7/2}^{{}^{\circ}}$ — 4*d″* ^2^D_5/2_
10616.31	1	9416.89	
10619.65	1	9413.93	4*d′* ^4^P_3/2_ — 7*p″* ${\;}^{2}P_{1/2}^{{}^{\circ}}$
10624.34	1	9409.77	5*p′* ${\;}^{4}D_{1/2}^{{}^{\circ}}$ — 7*s* ^4^P_3/2_
10629.45	18	9405.25	5*p* ${\;}^{4}P_{5/2}^{{}^{\circ}}$ — 4*d′* ^4^P_3/2_
10634.08	1	9401.15	4*d* ^4^D_3/2_ — 6*p′* ${\;}^{4}S_{3/2}^{{}^{\circ}}$
10638.82	15	9396.96	4*d′* ^2^F_7/2_ — 7*p′* ${\;}^{2}D_{5/2}^{{}^{\circ}}$
10660.80	3	9377.59	5*d* ${\;}^{2}P_{1/2}^{{}^{\circ}}$ — 4*d″* ^2^D_3/2_
10694.91	12	9347.68	4*d* ^4^D_7/2_ — 4*f* ${\left[5\right]}_{9/2}^{{}^{\circ}}$
10718.84	100	9326.81	4*d* ^4^D_7/2_ — 4*f* ${\left[2\right]}_{5/2}^{{}^{\circ}}$
10723.10	15	9323.11	4*d* ^4^D_3/2_—6*p′* ${\;}^{2}D_{3/2}^{{}^{\circ}}$
10742.14	1000	9306.58	4*d* ^4^D_7/2_—4*f* ${\left[3\right]}_{7/2}^{{}^{\circ}}$
10742.48	100	9306.29	$\{\begin{array}{c} 6s{\;}^{4}P_{5/2}—4f^{\prime }{\left[3\right]}_{5/2}^{{}^{\circ}}\\ 4d{\;}^{4}D_{7/2}—4f{\left[3\right]}_{5/2}^{{}^{\circ}} \end{array}$
10747.20	20	9302.20	6*s* ^4^P_5/2_—8*p* ${\;}^{4}D_{7/2}^{{}^{\circ}}$
10753.58	150	9296.68	5*p* ${\;}^{4}P_{3/2}^{{}^{\circ}}$—4*d′* ^4^P_5/2_
10754.13	200	9296.21	4*d* ^4^D_7/2_—4*f* ${\left[4\right]}_{7/2}^{{}^{\circ}}$
10755.92	3000	9294.66	4*d* ^4^D_7/2_—4*f* ${\left[4\right]}_{7/2}^{{}^{\circ}}$
10757.77	6	9293.06	6*s* ^4^P_5/2_—8*p* ${\;}^{4}P_{3/2}^{{}^{\circ}}$
10794.92	$\}{\;}_{10}^{5}$	9261.08	} 6*s* ^4^P_5/2_—4*f*′ ${\left[4\right]}_{7/2}^{{}^{\circ}}$
10795.10		9260.93	
10798.09	25	9258.36	5*p*′ ${\;}^{2}D_{5/2}^{{}^{\circ}}$—5*d* ^4^F_3/2_
10804.40	3	9252.95	6*s* ^4^P_5/2_— 8*p* ${\;}^{4}P_{5/2}^{{}^{\circ}}$
10810.05	300	9248.12	5*p* ${\;}^{4}D_{5/2}^{{}^{\circ}}$—4*d″* ^2^D_5/2_
10811.47	1*w*	9246.90	6*s* ^4^P_5/2_—4*f*′ ${\left[2\right]}_{5/2}^{{}^{\circ}}$
10815.65	6	9243.33	5*p′* ${\;}^{2}D_{5/2}^{{}^{\circ}}$—5*d* ^4^F_5/2_
10840.05	500*d*	9222.52	4*d* ^4^D_5/2_—(^1^D_2_) 5*p* ${\;}^{2}D_{5/2}^{{}^{\circ}}$
10843.86	2*w*	9219.28	4*d* ^4^F_5/2_—5*f* ${\left[1\right]}_{3/2}^{{}^{\circ}}$
10846.12	10	9217.36	4*d* ^4^D_3/2_—6*p′* ${\;}^{2}D_{5/2}^{{}^{\circ}}$
10869.70	100	9197.37	4*d* ^4^F_5/2_—5f ${\left[2\right]}_{5/2}^{{}^{\circ}}$
10871.62	90	9195.74	5*p′* ${\;}^{2}S_{1/2}^{{}^{\circ}}$—5*d* ^4^D_1/2_
10891.43	250	9179.02	4*d* ^4^F_5/2_—5*f* ${\left[3\right]}_{7/2,\;5/2}^{{}^{\circ}}$
10892.79	100	9177.87	4*d*^4^D_5/2_—(^1^D_2_)5*p* ${\;}^{2}D_{3/2}^{{}^{\circ}}$
10896.79	200	9174.50	4*d* ^4^F_5/2_— 5*f* ${\left[4\right]}_{7/2}^{{}^{\circ}}$
10908.62	60*d*	9164.55	4*d* ^4^D_5/2_—4*f* ${\left[1\right]}_{3/2}^{{}^{\circ}}$
10947.50	1	9132.00	4*d*′ ^2^P_1/2_—6f ${\left[1\right]}_{3/2}^{{}^{\circ}}$
10947.89	1	9131.68	4*d*′ ^2^P_1/2_—6*f* ${\left[1\right]}_{1/2}^{{}^{\circ}}$
10960.83	2 *w*	9120.90	4*d* ^4^D_1/2_—6*p*′ ${\;}^{4}D_{1/2}^{{}^{\circ}}$
10961.30	3	9120.51	4*d*′ ^2^P_1/2_— 6*f* ${\left[2\right]}_{3/2}^{{}^{\circ}}$
10973.48	500	9110.38	4*d* ^4^D_5/2_—4*f* ${\left[2\right]}_{5/2}^{{}^{\circ}}$
10979.00	300	9105.80	5*s*″ ^2^P_1/2_—5*p*′ ${\;}^{2}S_{1/2}^{{}^{\circ}}$
10982.30	100	9103.07	4*d* ^4^D_5/2_—4*f* ${\left[2\right]}_{3/2}^{{}^{\circ}}$
10989.51	1	9097.10	5*p′* ${\;}^{4}S_{3/2}^{{}^{\circ}}$—7*s* ^4^P_3/2_
10991.12	2	9095.76	4*d*′ ^2^F_5/2_—7*p′* ${\;}^{2}D_{3/2}^{{}^{\circ}}$
10997.85	600	9090.20	4*d* ^4^D_5/2_—4*f* ${\left[3\right]}_{7/2}^{{}^{\circ}}$
10998.28	400	9089.84	4*d* ^4^D_5/2_—4*f* ${\left[3\right]}_{5/2}^{{}^{\circ}}$
11007.42	10	9082.29	4*d* ^4^D_3/2_—6*p′* ${\;}^{2}S_{1/2}^{{}^{\circ}}$
11010.45	600	9079.79	4*d* ^4^D_5/2_—4*f* ${\left[4\right]}_{7/2}^{{}^{\circ}}$
11012.31	5	9078.26	5*p′* ${\;}^{2}D_{3/2}^{{}^{\circ}}$—5*d* ^4^F_5/2_
11013.22	20	9077.51	5*p*′ ${\;}^{2}D_{3/2}^{{}^{\circ}}$—5*d* ^4^P_1/2_
11018.55	1	9073.12	4*d*″ ^2^D_5/2_—5*f*′ ${\left[3\right]}_{5/2}^{{}^{\circ}}$
11024.25	10	9068.43	5*p* ${\;}^{4}P_{3/2}^{{}^{\circ}}$—4*d*′ ^4^P_3/2_
11039.80	60	9055.66	5*p* ${\;}^{2}S_{1/2}^{{}^{\circ}}$—5*d* ^4^D_3/2_
11045.74	800*d*	9050.79	5*p* ${\;}^{4}P_{5/2}^{{}^{\circ}}$—4*d*′ ^2^F_5/2_
11047.21	10	9049.58	$\{\begin{array}{c} 4d{\;}^{4}F_{3/2}—5f{\left[1\right]}_{3/2}^{{}^{\circ}}\\ 4d^{\prime \prime }{\;}^{2}D_{5/2}—5f^{\prime }{\left[4\right]}_{7/2}^{{}^{\circ}} \end{array}$
11047.62	17	9049.25	4*d* ^4^F_9/2_—7*p* ${\;}^{4}D_{7/2}^{{}^{\circ}}$
11074.08	40	9027.62	4*d* ^4^F_3/2_—5*f* ${\left[2\right]}_{5/2}^{{}^{\circ}}$
11093.51	$\}\begin{array}{c} 250\\ 100 \end{array}$	9011.81	} 5*p* ${\;}^{4}P_{1/2}^{{}^{\circ}}$—4*s*4*p*^6 2^S_1/2_
11094.24		9011.22	
11096.59	20	9009.31	4*d* ^4^F_3/2_—5*f* ${\left[3\right]}_{5/2}^{{}^{\circ}}$
11100.65	1	9006.02	6*s* ^4^P_3/2_—8*p* ${\;}^{4}D_{3/2}^{{}^{\circ}}$
11101.36	1	9005.44	4*d* ^4^D_1/2_—6*p′* ${\;}^{4}S_{3/2}^{{}^{\circ}}$
11161.72	1*w*	8956.74	5*p′* ${\;}^{4}S_{3/2}^{{}^{\circ}}$—7*s* ^4^P_5/2_
11194.99	100	8930.12	5*s*′ ^4^P_1/2_—5*p* ${\;}^{4}P_{1/2}^{{}^{\circ}}$
11197.14	3	8928.41	5*p*′ ${\;}^{2}D_{5/2}^{{}^{\circ}}$—5*d* ^4^F_7/2_
11225.08	250	8906.18	5*p* ${\;}^{4}P_{5/2}^{{}^{\circ}}$*—*4*d′* ^2^P_3/2_
11247.50	2	8888.43	5*p*′ ${\;}^{4}D_{1/2}^{{}^{\circ}}$—5*d* ^4^P_1/2_
11292.69	2	8852.86	4*d* ^4^D_3/2_—(^1^D_2_)5*p* ${\;}^{2}D_{5/2}^{{}^{\circ}}$
11316.93	10*w* (8)	8833.90	*5p* ${\;}^{4}D_{3/2}^{{}^{\circ}}$—6s′ ^4^P_1/2_
11325.97	1 (−)	8826.85	
11350.04	65 (27)	8808.13	4*d* ^4^D_3/2_—(^1^D_2_)5*p* ${\;}^{2}D_{3/2}^{{}^{\circ}}$
11367.25	4*w* (7)	$\{\begin{array}{c} 8794.\;79\\ 8794.\;24 \end{array}$	4*d* ^4^D_3/2_—4*f* ${\left[1\right]}_{3/2}^{{}^{\circ}}$
11367.97	4*w*		4*d* ^4^D_3/2_—4*f* ${\left[1\right]}_{1/2}^{{}^{\circ}}$
11437.61	9 (11)	8740.69	4*d* ^4^D_3/2_—4*f* ${\left[2\right]}_{5/2}^{{}^{\circ}}$
11447.27	18 (36)	8733.32	4*d* ^4^D_3/2_—4*f* ${\left[2\right]}_{3/2}^{{}^{\circ}}$
11464.58	35 (100)	8720.13	4*d* ^4^D_3/2_—4*f* ${\left[3\right]}_{5/2}^{{}^{\circ}}$
11501.31	1 (*m*)	8692.28	5*p* ${\;}^{4}D_{3/2}^{{}^{\circ}}$—6s′ ^2^P_3/2_
11508.77	30 (85)	8686.65	4*d* ^4^D_1/2_—6*p*′ ${\;}^{2}S_{1/2}^{{}^{\circ}}$
11583.71	10 (8)	8630.45	5*s*′ ^2^P_3/2_—5*p* ${\;}^{4}P_{1/2}^{{}^{\circ}}$
11597.64	1 (4)	8620.08	5*p′* ${\;}^{2}D_{5/2}^{{}^{\circ}}$—5*d* ^4^D_3/2_
11631.33	2 (2)	8595.12	5p*′* ${\;}^{2}D_{3/2}^{{}^{\circ}}$—5*d* ^4^D_1/2_
11636.08	1 (−)	8591.61	5*p*′ ${\;}^{4}S_{3/2}^{{}^{\circ}}$—5*d* ^4^F_3/2_
11640.94	1 (10)	8588.02	4*d* ^4^F_7/2_—7*p* ${\;}^{4}D_{5/2}^{{}^{\circ}}$
11666.20	20*d* (169)	8569.43	5*p* ${\;}^{4}P_{3/2}^{{}^{\circ}}$—4*d*′ ^2^P_3/2_
11742.85	400 (219)	8513.49	5*s*′ ^2^P_3/2_ 5*p* ${\;}^{4}D_{5/2}^{{}^{\circ}}$
11810.29	1 (1)	8464.88	4*d′* ^2^F_5/2_—6*f* ${\left[3\right]}_{7/2,\;5/2}^{{}^{\circ}}$
11819.40	2 (−)	8458.35	
11833.14	1 (−)	8448.53	5*p* ${\;}^{4}D_{3/2}^{{}^{\circ}}$—4*d″* ^2^D_3/2_
11870.77	4 (5)	8421.75	5*p*′ ${\;}^{2}D_{5/2}^{{}^{\circ}}$—5*d* ^4^D_5/2_
11883.95	4 (6)	8412.41	4*d* ^4^D_1/2_—(^1^D_2_)5*p* ${\;}^{2}D_{3/2}^{{}^{\circ}}$
11902.79	5 (19)	8399.09	4*d* ^4^D_1/2_—4*f* ${\left[1\right]}_{3/2}^{{}^{\circ}}$
11903.61	8 (16)	8398.51	4*d* ^4^D_1/2_—4*f* ${\left[1\right]}_{1/2}^{{}^{\circ}}$
11928.11	8 (13)	8381.26	4*d′* ^2^P_1/2_—4*f*′ ${\left[2\right]}_{3/2}^{{}^{\circ}}$
11990.57	15 (38)	8337.61	4*d* ^4^D_1/2_—4*f* ${\left[2\right]}_{3/2}^{{}^{\circ}}$
12088.15	2 (−)	8270.30	
12285.57	2 (*m*)	8137.40	4*d*′ ^2^F_7/2_—4*f*′ ${\left[4\right]}_{9/2}^{{}^{\circ}}$
12303.89	35 (275)	8125.29	5*s*′ ^4^P_1/2_— 5*p* ${\;}^{4}P_{3/2}^{{}^{\circ}}$
12354.43	1 (40)	8092.05	5*p* ${\;}^{4}P_{3/2}^{{}^{\circ}}$ 4*d′* ^2^P_1/2_
12368.95	1 *w*(170)	8082.55	5*p* ${\;}^{4}D_{3/2}^{{}^{\circ}}$—4*s*4*p*^6 2^S_1/2_
12809.50	1 (400)	7804.57	4*d* ^4^F_9/2_—4*f* ${\left[5\right]}_{11/2}^{{}^{\circ}}$

a λ4000.599 If this line itself is not of molecular origin, it is probably the blended hfs components of shorter wavelength for this transition, the long wavelength component having been lost in the molecular background.

b λλ4446.08, 4654.18, 6531.39. There are Cl lines of almost these same wavelengths.

c λ5905.66 This line appears to be the long wavelength hfs component of this transition, the other components having been obscured by the wing of the adjacent line.

d λ6151.10 Blended with ghost of adjacent strong line.

e λ7617.82 The transition $5p\prime {\;}^{2}D_{3/2}^{{}^{\circ}}—7d^{4}D_{3/2}$ probably contributes to this component.

**Table A3:** Lines of *Br I* observed radiometrically

Computed wavelength	Intensity	Computed wave number	Classification
*Å*		*K*	
11806.31	1	8467.73	4*d* ^4^F_7/2_—7*p* ${\;}^{4}P_{5/2}^{{}^{\circ}}$
12094.54	3	8265.93	5*p*′ ${\;}^{4}D_{1/2}^{{}^{\circ}}$—5*d* ^4^D_3/2_
12108.09	} 14	$\{\begin{array}{l} 8256.\;68\\ 8256.\;63 \end{array}$	5*p*′ ${\;}^{2}D_{5/2}^{{}^{\circ}}$—5*d* ^4^D_7/2_
12108.16			5*p*′ ${\;}^{2}D_{3/2}^{{}^{\circ}}$—5*d* ^4^D_5/2_
12303.93	275	8125.26	5*s*′ ^4^P_1/2_—5*p* ${\;}^{4}P_{3/2}^{{}^{\circ}}$
12354.56	40	8091.96	5*p* ${\;}^{4}P_{3/2}^{{}^{\circ}}$—4*d′*′ ^2^P_1/2_
12368.99	170	8082.52	5*p* ${\;}^{4}D_{3/2}^{{}^{\circ}}$—4*s*4*p*^6 2^S_1/2_
12513.40	20	7989.25	5*p* ${\;}^{4}P_{5/2}^{{}^{\circ}}$—6*s* ^4^P_3/2_
12647.55	10	7904.51	*4d*′ ^2^P_3/2_—4*f*′ ${\left[2\right]}_{5/2}^{{}^{\circ}}$
12755.94	60	7837.34	5*p* ${\;}^{4}D_{7/2}^{{}^{\circ}}$—4*d′*′ ^2^F_7/2_
12809.50	400	7804.57	4*d* ^4^F_9/2_—4*f* ${\left[5\right]}_{11/2}^{{}^{\circ}}$
12859.77	30	7774.06	4*d′* ^2^F_5/2_—4*f*′ ${\left[4\right]}_{7/2}^{{}^{\circ}}$
12861.74	30	7772.87	5*s*″ ^2^P_1/2_—5*p* ${\;}^{4}D_{3/2}^{{}^{\circ}}$
12897.12	48	7751.55	4*d* ^4^F_9/2_—4*f* ${\left[4\right]}_{9/2}^{{}^{\circ}}$
13048.21	38	7661.79	5*p* ${\;}^{4}D_{5/2}^{{}^{\circ}}$—4*d*′′ ^2^F_7/2_
13064.15	110	7652.44	5*p* ${\;}^{4}P_{3/2}^{{}^{\circ}}$—6*s* ^4^P_3/2_
13217.17	1700	7563.85	5*p* ${\;}^{4}P_{5/2}^{{}^{\circ}}$—6*s* ^4^P_5/2_
13328.04	10	7500.93	5*p*′ ${\;}^{2}S_{1/2}^{{}^{\circ}}$—6*s*′ ^4^P_1/2_
13349.73	36	7488.74	5*s*′ ^2^P_3/2_—5*p* ${\;}^{4}P_{5/2}^{{}^{\circ}}$
13392.33	20	7464.92	4*d*′ ^4^P_3/2_—4*f*′ ${\left[3\right]}_{5/2}^{{}^{\circ}}$
13484.87	9	7413.69	5*p*′ ${\;}^{4}D_{1/2}^{{}^{\circ}}$—6*s*″ ^2^P_1/2_
13584.48	30	7359.33	5*p*′ ${\;}^{2}S_{1/2}^{{}^{\circ}}$—6*s*′ ^2^P_3/2_
13674.13	300	7311.08	4*d* ^4^F_7/2_—4*f* ${\left[5\right]}_{9/2}^{{}^{\circ}}$
13808.19	} 23	$\{\begin{array}{l} \;7240.\;10\\ 7239.\;96 \end{array}$	6*p* ${\;}^{4}P_{1/2}^{{}^{\circ}}$—10*s* ^4^P_3/2_
13808.45			*4d′*′^4^P_5/2_—4*f*′ ${\left[3\right]}_{7/2}^{{}^{\circ}}$
13821.95	} 9	$\{\begin{array}{c} 7232.\;89\\ 7232.\;83\\ 7232.\;57 \end{array}$	4*d* ^4^D_1/2_—(^1^D_2_)5*p* ${\;}^{2}P_{3/2}^{{}^{\circ}}$
13822.07			6*p* ${\;}^{4}D_{7/2}^{{}^{\circ}}$—10*s* ^4^P_5/2_
13822.56			4*d*′ ^4^P_5/2_—8*p* ${\;}^{4}D_{7/2}^{{}^{\circ}}$
13833.14	750	7227.04	5*p* ${\;}^{4}P_{3/2}^{{}^{\circ}}$—6*s* ^4^P_5/2_
14078.51	10	7101.08	5*p*′ ${\;}^{4}S_{3/2}^{{}^{\circ}}$—6*s*″ ^2^P_1/2_
14290.98	} 68	$\{\begin{array}{c} 6995.\;51\\ 6994.\;91 \end{array}$	4*d* ^4^P_1/2_—4*f* ${\left[1\right]}_{3/2}^{{}^{\circ}}$
14292.20			4*d* ^4^P_1/2_—4*f* ${\left[1\right]}_{1/2}^{{}^{\circ}}$
14354.57	1800	6964.52	5*p* ${\;}^{4}D_{5/2}^{{}^{\circ}}$—6*s* ^4^P_3/2_
14439.14	344	6923.73	5*p*′ ${\;}^{2}D_{5/2}^{{}^{\circ}}$—6*s*′ ^2^P_3/2_
14488.21	105	6900.28	5*p*′ ${\;}^{2}D_{3/2}^{{}^{\circ}}$—6*s*′ ^4^P_1/2_
14599.73	338	6847.57	*5p* ${\;}^{4}P_{1/2}^{{}^{\circ}}$—6*s* ^4^P_3/2_
14607.80	21	6843.79	5*s*″ ^2^P_1/2_—5*p* ${\;}^{4}P_{1/2}^{{}^{\circ}}$
14888.70	1250	6714.67	5*p* ${\;}^{4}D_{7/2}^{{}^{\circ}}$—6*s* ^4^P_5/2_
14896.37	55	6711.21	5*p*′ ${\;}^{4}D_{1/2}^{{}^{\circ}}$—6*s*′ ^4^P_1/2_
14923.43	12	6699.04	4*d* ^4^F_5/2_—4*f* ${\left[2\right]}_{5/2}^{{}^{\circ}}$
14968.61	} 85	$\{\begin{array}{c} 6678.\;82\\ 6678.\;50 \end{array}$	4*d* ^4^F_5/2_—4*f* ${\left[3\right]}_{7/2}^{{}^{\circ}}$
14969.33			4*d* ^4^F_5/2_—4*f* ${\left[3\right]}_{5/2}^{{}^{\circ}}$
14991.94	89	6668.43	4*d* ^4^F_5/2_—4*f* ${\left[4\right]}_{7/2}^{{}^{\circ}}$
15003.82	35	6663.15	5*p* ${\;}^{4}P_{3/2}^{{}^{\circ}}$—4*d* ^4^F_5/2_
15052.95	10	6641.40	4*d* ^4^F_3/2_—(^1^D_2_)5*p* ${\;}^{2}D_{5/2}^{{}^{\circ}}$
15146.09	30	6600.56	4*d*″ ^2^D_5/2_—4*f*′ ${\left[3\right]}_{7/2}^{{}^{\circ}}$
15185.29	} 188	$\{\begin{array}{c} 6583.\;52\\ 6583.\;46\\ 6582.\;86 \end{array}$	5*p*″ ${\;}^{2}P_{3/2}^{{}^{\circ}}$—6*s*″ ^2^P_1/2_
15185.43			4*d* ^4^F_3/2_—4*f* ${\left[1\right]}_{1/2}^{{}^{\circ}}$
15186.82			4*d* ^4^F_3/2_—4*f* ${\left[1\right]}_{1/2}^{{}^{\circ}}$
15258.39	10	6551.98	4*d*″ ^2^D_5/2_—4*f*′ ${\left[4\right]}_{7/2}^{{}^{\circ}}$
15311.39	63	6529.30	4*d* ^4^F_3/2_—4*f* ${\left[2\right]}_{5/2}^{{}^{\circ}}$
15359.71	22	6508.76	4*d* ^4^F_3/2_—4*f* ${\left[3\right]}_{5/2}^{{}^{\circ}}$
15422.36	28	6482.32	4*d* ^4^D_7/2_—6*p* ${\;}^{4}P_{5/2}^{{}^{\circ}}$
15539.25	3	6433.56	5*p*′ ${\;}^{2}D_{5/2}^{{}^{\circ}}$—4*d*″ ^2^D_5/2_
15570.03	219	6420.84	5*p* ${\;}^{4}P_{3/2}^{{}^{\circ}}$—4*d* ^4^P_1/2_
15593.01	37	6411.38	5*p*″ ${\;}^{2}P_{1/2}^{{}^{\circ}}$—6*s*″ ^2^P_1/2_
15599.33	18	6408.78	5*p* ${\;}^{4}P_{5/2}^{{}^{\circ}}$—4*d* ^4^F_7/2_
15624.15	} 24	$\{\begin{array}{c} 6398.\;60\\ 6398.\;47 \end{array}$	5*p*′ ${\;}^{4}S_{3/2}^{{}^{\circ}}$—6*s*′ ^4^P_1/2_
15624.47			6*p* ${\;}^{4}D_{7/2}^{{}^{\circ}}$—9*s* ^4^P_5/2_
15804.98	7	6325.39	4*d* ^4^D_5/2_—6*p* ${\;}^{4}P_{3/2}^{{}^{\circ}}$
15890.32	2	6291.42	4*d*″ ^2^D_3/2_—4*f*′ ${\left[2\right]}_{5/2}^{{}^{\circ}}$
15948.40	20	6268.51	5*p*′ ${\;}^{2}D_{3/2}^{{}^{\circ}}$—4*d*″ ^2^D_5/2_
15955.01	8	6265.91	4*d* ^4^D_5/2_—6*p* ${\;}^{4}P_{5/2}^{{}^{\circ}}$
15977.73	47	6257.00	5*p*′ ${\;}^{4}S_{3/2}^{{}^{\circ}}$—6*s*′ ^2^P_3/2_
16253.66	90	6150.78	5*p* ${\;}^{4}D_{7/2}^{{}^{\circ}}$—4*d* ^4^F_5/2_
16584.66	250	6028.02	5*p* ${\;}^{4}P_{1/2}^{{}^{\circ}}$—4*d* ^4^F_3/2_
16625.37	10	6013.26	5*p*′ ${\;}^{4}S_{3/2}^{{}^{\circ}}$—4*d*″ ^2^D_3/2_
16657.89	10	6001.52	5*p*′ ${\;}^{2}S_{1/2}^{{}^{\circ}}$—4*d*′ ^4^P_3/2_
16731.19	1800	5975.23	5*p*′ ${\;}^{4}D_{5/2}^{{}^{\circ}}$—4*d* ^4^F_5/2_
17254.05	105	5794.16	5*p*′ ${\;}^{2}D_{5/2}^{{}^{\circ}}$—4*d*′ ^4^P_5/2_
17335.81	25	5766.83	5*p*′ ${\;}^{4}S_{3/2}^{{}^{\circ}}$—4*d″* ^2^D_5/2_
17801.50	330	5615.97	5*p* ${\;}^{4}P_{1/2}^{{}^{\circ}}$—4*d* ^4^P_1/2_
17982.00	40	5559.60	5*p* ${\;}^{4}D_{7/2}^{{}^{\circ}}$—4*d* ^4^F_7/2_
18191.08	60	5495.70	5*p*″ ${\;}^{2}P_{3/2}^{{}^{\circ}}$—4*d*″ ^2^D_3/2_
18212.72	10	5489.17	4*d′* ^2^F_7/2_—6*p*′ ${\;}^{2}D_{5/2}^{{}^{\circ}}$
18510.48	180	5400.87	5*p*′ ${\;}^{2}D_{3/2}^{{}^{\circ}}$—4*d′* ^4^P_3/2_
18568.31	500	5384.05	5*p* ${\;}^{4}D_{5/2}^{{}^{\circ}}$*—4d* ^4^F_7/2_
18779.29	313	5323.56	5*p*″ ${\;}^{2}P_{1/2}^{{}^{\circ}}$—4*d*″ ^2^D_3/2_
19045.07	547	5249.27	5*p*″ ${\;}^{2}P_{3/2}^{{}^{\circ}}$—4*d″* ^2^D_5/2_
19181.99	} 266	{5211.80 5211.48	5*p*′ ${\;}^{4}D_{1/2}^{{}^{\circ}}$—4*d′* ^4^P_3/2_
	
19183.17			5*p*′ ${\;}^{2}D_{5/2}^{{}^{\circ}}$—4*d*′ ^2^F_5/2_
19317.02	120	5175.37	4*d* ^4^F_9/2_—6*p* ${\;}^{4}D_{7/2}^{{}^{\circ}}$
19497.62	313	5127.43	5*p*′ ${\;}^{4}S_{3/2}^{{}^{\circ}}$—4*d*′ ^4^P_5/2_
19606.57	263	5098.94	5*p* ${\;}^{4}D_{3/2}^{{}^{\circ}}$—4*d* ^4^F_3/2_
19733.62	3450	5066.11	5*p* ${\;}^{4}D_{7/2}^{{}^{\circ}}$—4*d* ^4^F_9/2_
19810.58	452	5046.43	5*p*′ ${\;}^{2}D_{3/2}^{{}^{\circ}}$—4*d*′ ^2^F_5/2_
19894.71	68	5025.09	5*p*′ ${\;}^{2}S_{1/2}^{{}^{\circ}}$—4*d^r^* ^2^P_1/2_
19925.72	138	5017.27	5*p* ${\;}^{4}P_{3/2}^{{}^{\circ}}$—4*d* ^4^D_1/2_
20073.55	7	4980.32	4*d*′ ^2^F_7/2_—4*f* ${\left[4\right]}_{9/2}^{{}^{\circ}}$
20162.50	138	4958.35	5*p* ${\;}^{4}P_{5/2}^{{}^{\circ}}$—4*d* ^4^D_3/2_
20248.38	8	4937.32	(^1^D_2_)5*p* ${\;}^{2}F_{5/2}^{{}^{\circ}}$—(^1^D_2_)4*d* ^2^G_7/2_
20281.73	500	4929.20	5*p* ${\;}^{4}D_{3/2}^{{}^{\circ}}$—4*d* ^4^F_5/2_
20405.97	7	4899.19	5*p*′ ${\;}^{4}S_{3/2}^{{}^{\circ}}$—4*d*′ ^4^P_3/2_
20624.67	547	4847.24	5*p*′ ${\;}^{2}D_{5/2}^{{}^{\circ}}$—4*d*′ ^2^F_7/2_
21093.83	43	4739.43	4*d* ^4^F_7/2_—6*p* ${\;}^{4}D_{5/2}^{{}^{\circ}}$
21213.11	14	4712.78	5*p*′ ${\;}^{4}D_{1/2}^{{}^{\circ}}$—4*d*′ ^2^P_3/2_
21285.74	17	4696.70	(^1^D_2_)5*p* ${\;}^{2}F_{7/2}^{{}^{\circ}}$—(^1^D_2_)4*d* ^2^G_9/2_
21330.29	37	4686.89	5*p* ${\;}^{4}D_{3/2}^{{}^{\circ}}$—4*d* ^4^P_1/2_
21631.91	359	4621.54	5*p* ${\;}^{4}P_{3/2}^{{}^{\circ}}$—4*d* ^4^D_3/2_
21743.98	14	4597.72	6*s* ^4^P_3/2_—(^1^D_2_)5*p* ${\;}^{2}P_{3/2}^{{}^{\circ}}$
21787.24	469	4588.59	5*p* ${\;}^{4}P_{5/2}^{{}^{\circ}}$—4*d* ^4^D_5/2_
21801.59	6	4585.57	5*p*′ ${\;}^{2}S_{1/2}^{{}^{\circ}}$—6*s* ^4^P_3/2_
22720.19	60	4400.17	5*p*′ ${\;}^{4}S_{3/2}^{{}^{\circ}}$—4*d*′ ^2^P_3/2_
22865.65	950	4372.18	5*p* ${\;}^{4}P_{5/2}^{{}^{\circ}}$—4*d* ^4^D_7/2_
23404.15	12	4271.58	4*d* ^4^F_5/2_—6*p* ${\;}^{4}D_{3/2}^{{}^{\circ}}$
23513.15	206	4251.78	5*p* ${\;}^{4}P_{3/2}^{{}^{\circ}}$—4*d* ^4^D_5/2_
23732.96	40	4212.40	5*p* ${\;}^{4}P_{1/2}^{{}^{\circ}}$—4*d* ^4^D_1/2_
24099.97	2	4148.25	4*d* ^4^F_5/2_—6*p* ${\;}^{4}D_{5/2}^{{}^{\circ}}$
